# Dry Eye Subtype Classification Using Videokeratography and Deep Learning

**DOI:** 10.3390/diagnostics14010052

**Published:** 2023-12-26

**Authors:** Norihiko Yokoi, Natsuki Kusada, Hiroaki Kato, Yuki Furusawa, Chie Sotozono, Georgi As. Georgiev

**Affiliations:** 1 Department of Ophthalmology, Kyoto Prefectural University of Medicine, Kyoto 602-0841, Japan; nkusada9@koto.kpu-m.ac.jp (N.K.); hiro-kat@koto.kpu-m.ac.jp (H.K.); yukif@koto.kpu-m.ac.jp (Y.F.); csotozon@koto.kpu-m.ac.jp (C.S.); 2 Department of Optics and Spectroscopy, Faculty of Physics, St. Kliment Ohridski University of Sofia, 1164 Sofia, Bulgaria; ggeorg@phys.uni-sofia.bg

**Keywords:** dry eye, subtype classification, videokeratography, deep learning, artificial intelligence, tear-film-oriented diagnosis

## Abstract

We previously reported on ‘Tear Film Oriented Diagnosis’ (TFOD), a method for the dry eye (DE) subtype classification using fluorescein staining and an examination of fluorescein breakup patterns via slit-lamp biomicroscopy. Here, we report ‘AI-supported TFOD’, a novel non-invasive method for DE subtype classification using videokeratography (VK) and “Blur Value” (BV), a new VK indicator of the extent of blur in Meyer-ring images and deep learning (DL). This study involved 243 eyes of 243 DE cases (23 males and 220 females; mean age: 64.4 ± 13.9 (SD) years)—i.e., 31 severe aqueous-deficient DE (sADDE) cases, 73 mild-to-moderate ADDE (m/mADDE) cases, 84 decreased wettability DE (DWDE) cases, and 55 increased evaporation DE (IEDE) cases diagnosed via the fluorescein-supported TFOD pathway. For DL, a 3D convolutional neural network classification model was used (i.e., the original image and BV data of eyes kept open for 7 s were randomly divided into training data (146 cases) and the test data (97 cases), with the training data increased via data augmentation and corresponding to 2628 cases). Overall, the DE classification accuracy was 78.40%, and the accuracies for the subtypes sADDE, m/mADDE, DWDE, and IEDE were 92.3%, 79.3%, 75.8%, and 72.7%, respectively. ‘AI-supported TFOD’ may become a useful tool for DE subtype classification.

## 1. Introduction

Due to the worldwide increase in elderly populations, the use of visual display terminals, contact lens wearers, etc., dry eye (DE), which is one of the most common ocular surface (OS) disorders (OSDs) frequently encountered in the clinical setting, is increasing [1]. Moreover, DE reportedly has a significant impact on the economy as a result of reduced work efficiency due to DE-related symptoms such as ocular discomfort and/or visual disturbance [2]. According to the Asia Dry Eye Society, DE is defined as “a multifactorial disease characterized by unstable tear film causing a variety of symptoms and/or visual impairment, potentially accompanied by ocular surface damage” [3]. Similarly, the Dry Eye Workshop II (DEWS II) report defined DE as “a multifactorial disease of the ocular surface characterized by a loss of homeostasis of the tear film and accompanied by ocular symptoms, in which tear film instability and hyperosmolarity, OS inflammation and damage, and neurosensory abnormalities play etiological roles” [4]. Based on those definitions, tear film (TF) instability is an essential abnormality for the pathophysiology of DE, and proper evaluation of TF instability is vital for a correct diagnosis of the disease.

Recently, a new diagnostic pathway for DE, termed ‘TF oriented diagnosis’ (TFOD), has advanced in Japan [5,6,7], in which the differential diagnosis of DE includes (1) severe aqueous deficient DE (ADDE) (sADDE), (2) mild-to-moderate ADDE (m/mADDE), (3) decreased wettability dry eye (DWDE), and (4) increased evaporation DE (IEDE). Via the TFOD pathway, a proper diagnosis of those DE subtypes can be achieved based on fluorescein-stained aqueous TF dynamics and fluorescein breakup (BU) patterns (BUPs) (FBUPs). In addition, based on FBUPs, not only the DE subtype but also the insufficient component of the OS responsible for the TF BU in each DE subtype can be suggested, and the most effective topical eye therapy currently available in each country to treat TF instability can be proposed. As FBUPs, eight representative FBUPs can be differentiated, in which area break (AB), partial AB (pAB), line break (LB), spot break (SB), dimple break (DB), random break (RB), LB with rapid expansion (RE), and RB with RE, respectively, correspond to sADDE (AB and pAB), m/mADDE (LB), and DWDE (SB, DB, LB with RE, and RB with RE, precisely). Although RB with RE corresponds to IEDE with DWDE, DWDE is thought to be the predominant pathophysiology of RB with RE [5,6,7]. In addition, the respective insufficient components for sADDE, m/mADDE, DWDE, and IEDE are severely diminished aqueous tears, mildly to moderately diminished aqueous tears, membrane-associated mucins, especially the longest, MUC16, which can maintain corneal surface wettability [8,9], and TF lipid layer or secretory mucins, which are thought to suppress the evaporation of the aqueous tears in the TF aqueous layer [10,11]. It has been reported that among those DE subtypes, DWDE presenting the BUs SB, DB, or RE might be involved in short BU time type DE, which is characterized by relatively severe symptoms despite normal tear volume and minimal involvement of corneal epithelial damage (CED) [3,5,12,13]. Furthermore, DWDE is often overlooked and is not included in the DE classification proposed in the DEWSII report [4], although it is included in that of the Asia Dry Eye Society [14]. Thus, to appropriately treat DWDE, a proper diagnosis is important.

When implementing TFOD based on the classification of FBUPs, the examination of tears by slit-lamp microscopy after staining the tears with sodium fluorescein via a fluorescein test strip, etc., is essential. However, fluorescein staining is a somewhat invasive procedure, and the results can differ (i.e., be non-reproducible, inaccurate, or inconsistent) due to the method being used to apply the strip to the eye, which can vary among examiners [5]. To overcome those intrinsic fluorescein staining problems, various more advanced non-invasive methods have been developed for the evaluation of TF instability in DE, including videointerferometry [7,15], videothermography [16], wavefront analysis [17,18], videokeratoscopy [19], and videokeratography (VK) [20,21,22]. However, to the best of our knowledge, no non-invasive methods have been developed to simultaneously assess both TF stability and CED in any type of DE and indicate a subtype classification. However, due to our recent advancements in VK involving the use of a “Blur Value” (BV), a new indicator for the assessment of the extent of blur in Meyer-ring (MR) images, we are now able to successfully differentiate sADDE, m/mADDE, DWDE, and IEDE non-invasively [21,22].

There have been numerous attempts to diagnose and assess DE via the use of artificial intelligence (AI), in which machine learning, especially deep learning (DL), is applied for the evaluation of DE and other OSDs. For example, for the screening of characteristic abnormalities in DE and other OSDs, DL has been adopted for the examination of DE aspects such as tear meniscus height [23], fluorescein-stained corneal photographs [24], interferometry [25], in vivo laser confocal microscopy [26], and meibography [27,28], to detect and/or assess those abnormalities. However, to date, there have been no published studies reporting a successful use of AI for the classification of the DE subtype. Thus, if AI could be successfully adopted into the TFOD pathway in combination with TF-oriented therapy (TFOT), it might be an ideal application in the clinical setting, as it is theorized that in the fluorescein-staining-supported TFOD/TFOT pathway [5,6,7], AI-supported non-invasive TFOD would be useful.

Hence, the aim of this present study was to adopt our newly developed BV-supported VK system to AI using DL in order to classify the DE subtype [21,22].

## 2. Methods

### 2.1. Subjects

This retrospective study involved 243 eyes that were followed at the Dry Eye Outpatient Clinic of the Department of Ophthalmology, Kyoto Prefectural University of Medicine, Kyoto, Japan, between 4 January 2019 and 8 October 2021. Details of the patient data are shown in Table 1.

All patients were diagnosed as DE based on the current Japanese diagnostic criteria [29], i.e., exhibiting the DE-related symptoms of eye discomfort and/or visual disturbance and a fluorescein BU time of ≤5 s, and the eyes deemed eligible for inclusion were those with more severe symptoms. In cases in which the severity of the symptoms was the same in both eyes, right-eye data were used. Prior to enrollment in the study, confirmation was obtained from all patients that no eye drop medications had been used for at least 1 h prior to the initial examination.

Patients with any of the following conditions were excluded from the study: (1) patients with an eyelid disease such as blepharoptosis, lagophthalmos, blepharospasm, entropion, or ectropion, (2) patients with severe conjunctivochalasis, and (3) patients with any history of undergoing eye surgery, such as for the puncta, an OSD other than DE, the eyelid, and glaucoma. All patients deemed inappropriate for involvement in this study were excluded via a consensus by three ophthalmologists (N.Y., N.K., and H.K.).

### 2.2. Study Protocol

On the day of examination, FBUPs were assessed via the following 4 steps [5,6,7]:

(1) Two drops of saline were instilled onto a fluorescein test strip (Ayumi Pharmaceutical Corporation, Tokyo, Japan) that was then vigorously shaken to minimize, as much as possible, the amount of aqueous fluid on the strip. Using this procedure, no significant difference in meniscus height, implying no significant tear volume increase, was noted between fluorescein staining and without fluorescein staining (unpublished data);
(2) The test strip was then gently placed a bit temporal to the center of the patient’s lower lid margin, with the patient then being verbally instructed to gently blink several times to mix the fluorescein with the aqueous tears;
(3) The patient was then instructed to briskly open the eye 3 consecutive times after gently closing the eye, followed by keeping the eye open until fluorescein BU appears for as long as 10 s at each blink. Those procedures were observed 3 times with a slit-lamp biomicroscope using the appropriate filters for observing the upward movement of the fluorescein-stained aqueous tears and FBUPs and were video-recorded;
(4) The FBUPs and corresponding DE subtypes were then classified.

The eyes that repeatedly presented typical FBUPs, including AB, partial AB, LB, SB, DB, RB, LB with RE, and RB with RE at each of the 3 successive blinks, were enrolled in this study. They were, respectively, categorized as either ADDE (i.e., sADDE (when AB or partial AB was observed) or m/mADDE (when LB was observed)), DWDE (when SB, DB, LB with RE, or RB with RE was observed), or IEDE (when RB was observed). Table 2 shows the relationship between DE subtypes and FBUPs. As a data sample, eight typical example images (150 original images acquired by VK) of 8 types of BUPs including DB, LB with RE, RB with RE and SB for DWDE, RB for IEDE, LB for mild to moderate ADDE, and AB and partial AB for severe ADDE are available in the Supplementary files.

More than 10 min after the classification of the FBUPs and corresponding DE subtype, a time-dependent change in precorneal TF behavior when the eye is kept open was acquired through the use of a VK instrument (RET-700; Rexxam Co., Ltd., Osaka, Japan), in which two data sets, including the original VK data and the VK data with BV [21,22], the new indicator that enables assessment of the corneal-surface abnormalities from the point of the extent of the disturbance of the MRs, were obtained using the software incorporated in the instrument [21,22]. Those two data sets during 7 s of eye opening were used for DL.

### 2.3. Clinical Assessment

**Figure 1:**
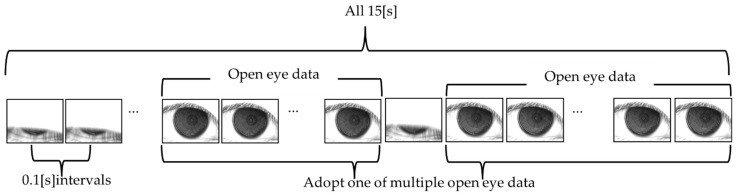
Schema showing an outline of the open-eye data sets acquired from video data.

As shown in Figure 1, the original video data of the subject’s eye were acquired via the VK instrument for 15 [s] (150 [frame]) at 0.1 [s] intervals. The resolution of the video data is 640 × 480 (pixel), and each pixel is an 8-bit grayscale. The VK data with BV was obtained using the original video data. Then, the video data obtained from 243 eyes in total were divided into independent open-eye data sets comprising the original VK data and the VK data with BV, and a single data set was used for the analysis. If the eye was kept open only once or was opened for the entire 15 s, the data were selected as they were.

The video data of all 243 eyes was cropped in the range of 200 (pixel) in the upper, lower, right, and left directions from the center of the MRs, with a resolution of 400 × 400 (pixel), in order to analyze only the data within which MRs are included. To keep the data size consistent, the time for data acquisition was adjusted to 7 (s) (70 (frames)) from the start of the eye opening (data for time over 7 (s) was deleted). If the eye could not be kept open for as long as 7 (s), 0 data points (black data) were added to the missing frames. For example, if an eye was kept open for as long as only 5 s, with data of 0–2 s being missed, 0 data points for 2 s (20 frames) were added as the missing data for the 5 s open eye data.

To prepare the data for DL, video data of all 243 eyes were randomly divided into a ratio of training data 6 (146 eyes)/test data 4 (97 eyes) using the stratified hold-out method for learning model evaluation. The BUP data were also divided into a ratio of 6:4. As for the training data and the test data, in order to reduce the training and inference time and the number of parameters, for all 97 video data, 400 × 400 (pixel) video data were cropped in the range of 150 (pixel) in the upper, lower, right, and left directions from the center of the video data, with a resolution of 300 × 300 (pixel), and its resolution was further lowered by half, 150 × 150 (pixel).

To improve generalization performance, data augmentation was performed on the training data so that the amount of data was increased for all 146 video data points. Data augmentation was performed as follows (1)–(3):

(1) Two Rotations: +5 [deg.] and −5 [deg.];
(2) Two Rescalings: 3 [%] enlarging and 3 [%] reducing;
(3) Horizontal flipping.

Increased the total data by a factor of 18, bringing the total data count to 2628. The breakdown of the number of test data and training data is shown in Table 3.

In creating the rotated and scaled images, sub-pixel interpolation was performed by the bicubic interpolation method [30]. The resolution was reduced to 150 × 150 (pixel) in the same way as the test data for 2628 increased video data. The reason for cropping 640 × 480 (pixel) video data at 400 × 400 (pixel) and then cropping it again at 300 × 300 (pixel) was to prevent the edges of the video data from being lost when the image is reduced or rotated in augmentation.

**Figure 2:**
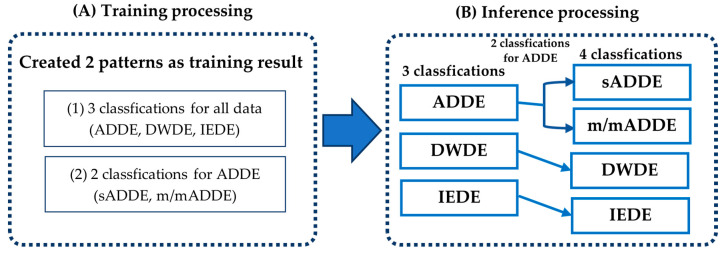
Schema showing the outlines of the (**A**) training processing and the (**B**) inference processing.

A subtype classification of DE was conducted in a standard way in training and inference processing, as shown in Figure 2, using the video data created by the above-mentioned method. First, the training processing was performed using the training data, and the trained models of 3 classifications and 2 classifications were prepared as follows:

(1) Three Classifications: (sADDE, m/mADDE), DWDE, and IEDE data were used as inputs for the trained model data;
(2) Two Classifications: sADDE and m/mADDE data were used as inputs for the trained model data.

Second, inference processing was carried out by inputting the test data into the newly created trained model. It was first classified into the ADDE, DWDE, or IEDE subtypes described in (1) above, and then classified further into either sADDE or m/mADDE, as described in (2) above, to differentiate between sADDE and m/mADDE when the data are classified as ADDE. Based on the above results, the probability of 4 classifications was calculated from the classification probabilities of 3 classifications and 2 classifications.

**Figure 3:**
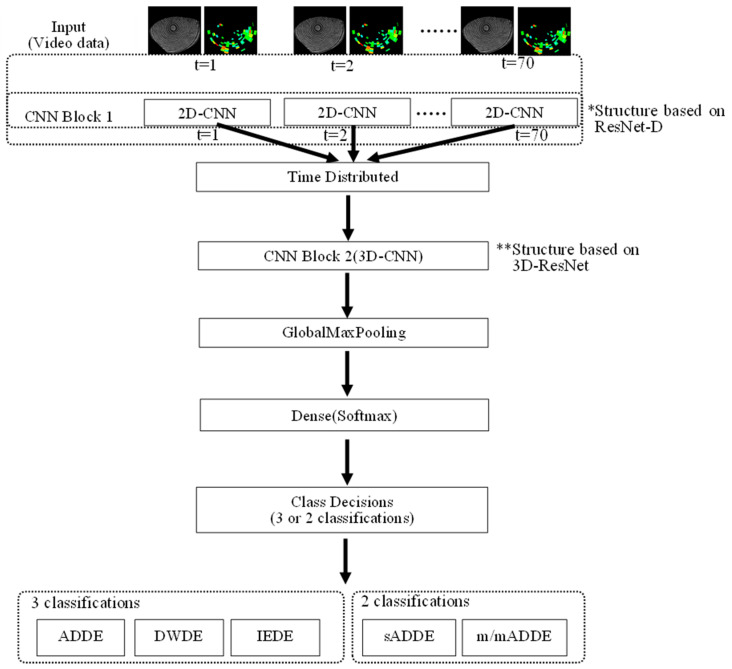
Schema showing the network structure of DL, i.e., input layer, CNN1 Block1 (2D-CNN) based on ResNet-D [31], time-distributed layer, CNN Block2 (3D-CNN) based on 3D-ResNet, Global Max Pooling layer, and Dense layer that outputs probabilities for each subtype.

The network structure of the DL model is shown in Figure 3, with further details shown in Table 4. In this analysis, a model of DL with a structure consisting of 2-dimensional (D) and 3-D convolutional deep neural networks (2D-CNN and 3D-CNN, respectively) was created. First, the input dimension was set to 5, including 3638 of training data, original video data with the resolution of 150 × 150 [pixel], 2 channels of the BV video data (8-bit for each pixel), and 70 [frames], and the features were extracted from CNN1 Block1 (2D-CNN) based on ResNet-D with a 2-D convolution layer [31], 2 layers of the 2-D convolution layer, a 2-D max pooling layer, and a 2-D average pooling layer. At the end of CNN Block 1, training stabilization was performed by disabling the neurons with a constant probability in the dropout layer. In this method, the probability is set to 25%. Second, a 3-D (vertical, horizontal, and temporal) structure was created with a time-distributed layer for the obtained features. The features were obtained 3-dimensionally with a 3-D convolution layer and 2-D average pooling layer by entering the result into CNN Block2 (3D-CNN) based on 3D-ResNet [32]. For both CNN1 Block 1 and CNN1 Block 2, a batch-normalized layer was placed after the convolution layer to stabilize the training by batch normalization. ReLU (Rectified Linear Units) was used for the activation function. Then, the features thus far obtained were made to be one-dimensional by a Global Max Pooling layer, and the probability that 3 or 2 classifications became the largest in the Dense layer with Softmax function as activation function was classified as the subtype. The mini-batch size for training was 64, an Adam optimizer was used as the optimization algorithm, the learning rate was set to 0.001, and categorical cross entropy was used as the loss function. The number of epochs was set to 60. In addition, the trained models of both 3 classifications and 2 classifications with the same structure as that in Figure 3 were used.

## 3. Results

**Figure 4:**
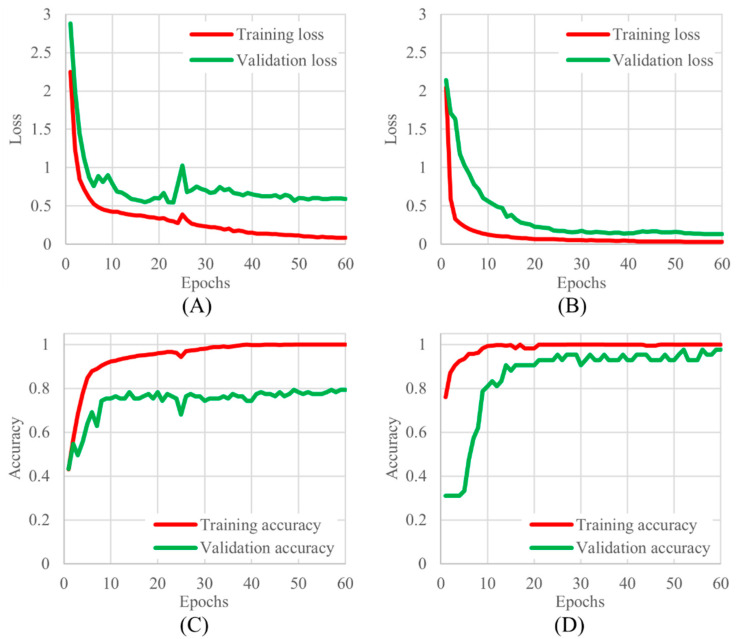
The learning curves for 3 and 2 classifications, (**A**) 3 classifications of training and validation loss, (**B**) 2 classifications of training and validation loss, (**C**) 3 classifications of training and validation accuracy, and (**D**) 2 classifications of training and validation accuracy.

The learning curves for 3-class and 2-class classifications are shown in Figure 4. Based on the learning curves, the 60th epoch, where the changes in loss and accuracy converged, was adopted as the trained model.

**Figure 5:**
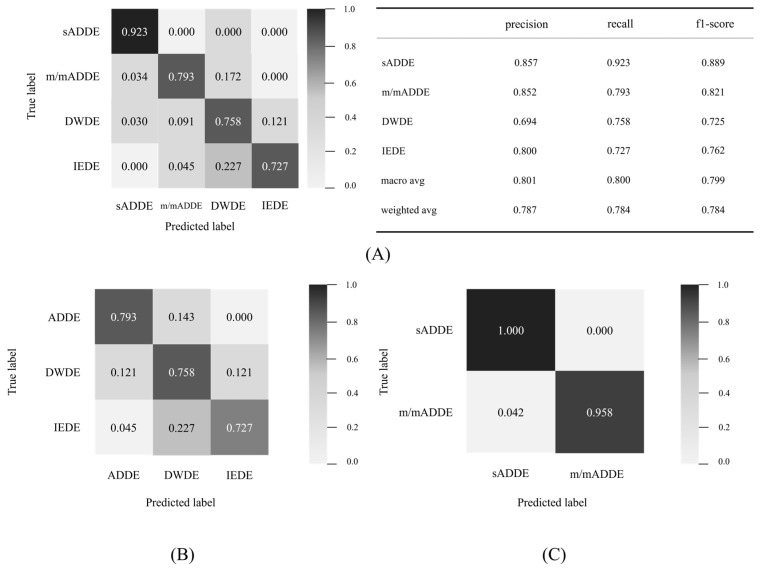
Detail of the confusion matrix and 4 classification results, (**A**) detail of confusion matrices of 4 classifications and matrices for evaluation, (**B**) confusion matrix of 3 classifications, and (**C**) confusion matrix of 2 classifications.

As shown in Figure 2, the inference processing was performed by entering the test data into the newly created trained model data to calculate the possibility of four classes for all 97 data points, and the class of the maximum probability was classified as a subtype. A confusion matrix was then created from the result (Figure 5). The following are shown in Figure 5, respectively: (A) the table for the confusion matrix of the four classifications and the precision of each subtype, and the table for recall and f1-score (harmonic average of precision and recall), their simple average (macro avg), and weighted average (weighted avg) of the number of data; (B) the result of three classifications; and (C) the result of two classifications. It was found that the accuracy of the final four classifications was 0.784.

For each subtype, sADDE was less prone to being misinterpreted than the other subtypes, with recall exceeding 0.9 and a precision of 0.857, which were both higher than those in the other subtypes. m/mADDE, DWDE, and IEDE were prone to misinterpret each other with lower recall and precision compared to sADDE, but the recall was above 0.7 in all cases. However, DWDE had lower precision, and it was more frequently prone to being misinterpreted as other types, such as m/mADDE and IEDE. m/mADDE and IEDE had precision equal to or greater than 0.8 and were less prone to being misinterpreted. m/mADDEs and IEDEs had a precision of 0.8 or higher and were less misinterpreted as other types than DWDEs. m/mADDE, DWDE, and IEDE had lower BVs, and they were prone to misinterpret each other due to the presence of hard-to-distinguish data.

**Figure 6:**
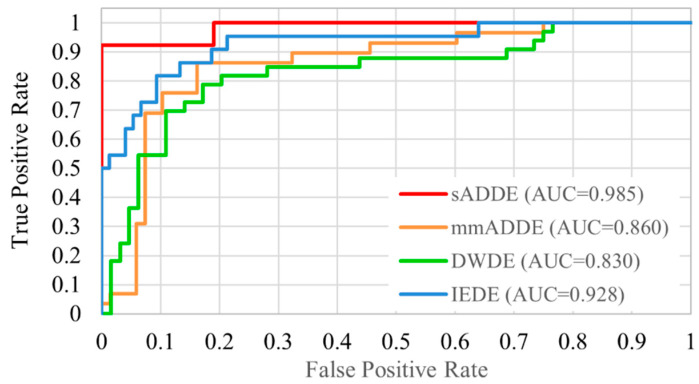
Receiver operating characteristic curve of 4 classifications.

The receiver operating characteristic analysis curves for each subtype are shown in Figure 6. The receiver operating characteristic curve was plotted graphically by calculating the true-positive rate and false-positive rate. Since this study employed multiple classification methods, it was calculated as the sum of the rate of true positive rate as a correct subtype and the rate of false positive rate as an incorrect subtype (1 minus true positive rate). The classification accuracy for each subtype was relatively good, as our findings showed that the area under the curve of sADDE, IEDE, m/mADDE, and DWDE was 0.985 (very high), 0.928, 0.860, and 0.830, respectively, with all being over 0.8.

**Figure 7:**
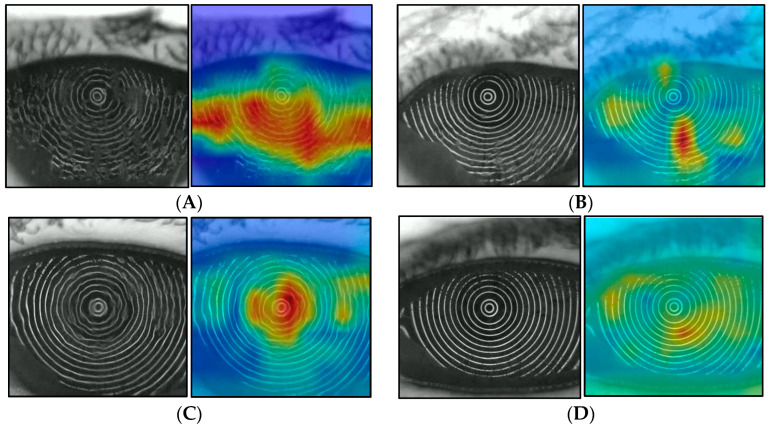
Heat map with Grad-CAM++ (left: original image; right: heat map), (**A**) example of sADDE (5.0 s after opening eye), (**B**) example of m/mADDE (5.0 s after opening eye), (**C**) example of DWDE (immediately after opening eye), and (**D**) example of IEDE (5.0 s after opening eye).

A heat map that can visually confirm the judgmental basis of the CNN model was created by the class activation map (CAM). Grad-CAM++ was used for the algorithm of the heat map preparation [33], and the gradient was calculated for the weight of CNN Block1, as shown in Figure 3. This heat map is called a Saliency Map [32], which emphasizes the pixels that were used as decision-making evidence during DL, with blue being weak and green to yellow to red indicating increasingly strong features. Figure 7 shows one example for sADDE, m/mADDE, DWDE, and IEDE each. The BUP in the example in Figure 7 shows AB for sADDE, LB for m/mADDE, SB for DWDE, and RB for IEDE. As an overall trend, the areas with strong disturbance or blur of the MRs were shown in yellow to red, and the areas with weak disturbance or blur, no disturbance or blur, or no MR were shown in green to blue. In the case of sADDE (AB), the heat map shows a wide area of red, with no upward movement of the aqueous layer and greater disturbance of the MR pattern over the entire corneal surface. In the case of m/mADDE (LB), the heat map shows a long red area extending vertically and disturbance of the lines. In the case of DWDE (SB), the heat map shows a circular red-to-yellow area near the center of the cornea. IEDE (RB) was a relatively mild case, and even in this case, there are few red areas; the yellow-to-light-blue areas were generally faint on the heat map, and the disturbance of the ring pattern was relatively small.

## 4. Discussion

For the diagnosis of DE in this study, FBUPs were used to classify three DE subtypes, including ADDE, DWDE, and IEDE, with ADDE further classified into sADDE and m/mADDE [5,6,7]. In addition, for the recruitment of typical DE patients, representative cases presenting typical FBUPs, with those FBUPs appearing repeatedly in three successive blink times, were enrolled. Thus, we deemed it reasonable to consider that the same BUPs as those of FBUPs must be reflected upon the distortion of the MRs when the eyes were evaluated via VK at more than 10 min after the classification of FBUPs and DE subtypes based on the FBUPs. Moreover, the DE subtype classified by FBUPs could be appropriately used as the ground truth, i.e., the correct answer for DL. In fact, our findings demonstrated that the accuracy of DL for calculating the overall DE classification was 78.4% and that the accuracies for DE subtypes sADDE, m/mADDE, DWED, and IEDE were 92.3%, 79.3%, 75.8%, and 72.7%, respectively. Moreover, it was demonstrated that a higher accuracy of 97.2% was found in the two classifications for ADDE, in which differential diagnosis between sADDE and m/mADDE was achievable with very high accuracy. This was also found to be true for the greater accuracy of sADDE in the four classifications. However, that is what we expected, considering that the subtype sADDE presents a remarkably greater MR disturbance, which was not detected in the other subtypes. In addition, although the accuracy of the three classifications was 79.4%, it was just slightly higher than that of the final four classifications, and there was only one incorrect interpretation. Therefore, the performance of the algorithm for the four classifications is deemed to be determined by the three classifications. Considering that subtype sADDE had the lowest number of test data (i.e., 13), while DWDE had the highest number (i.e., 33), and that the weighted average of the f1-score was the same as that of accuracy and there was no event in which only accuracy was higher, this suggests that the bias in the amount of data per subtype had almost no effect.

A heat map can tell how and from what aspect AI evaluates the disturbance of the MR within the corneal surface in DE, and in the case of sADDE corresponding to AB, the heat map showed red in a wider area with no time-dependent change. That finding was interpreted as precorneal TF comprising the lipid layer and aqueous layer presenting no upward movement after the eye was opened and only the resultant CED due to the lack of TF being reflected upon the distortion of the MRs [5,6,7]. In contrast, in m/mADDE corresponding to LB, the heat map showed a long red area extending vertically while accompanying the time-dependent enhancement of disturbance within the lower cornea, which may reflect the counteraction between the meniscus-induced thinning and upward movement of the aqueous layer simultaneously occurring within the lower part of the cornea in m/mADDE after the eye is opened [5,6,7,11,34].

In the case of DWDE, in which a representative BUP is SB, the heat map showed a spot-like smaller area with red to yellow appearing typically at the central part of the cornea immediately after eye opening, which is recognized as remarkable spot-like disturbances with time-dependent lessening when the eye is kept open [5,6,7,21].

Viewing the time-dependent distortional changes of the corneal surface, which are expected to be derived from TF instability and/or BU and CED [22], we considered that the use of the CAM heat map would help to visualize the basis for AI judgment via DL to some extent and that the combination of the CAM heat map with the subtype classification results via AI would be of great value for ophthalmologists to diagnose DE subtype via the TFOD pathway [5,6,7]. In addition, considering that VK is non-invasive with no aid of fluorescein, it is possible that even medical staff members could play some roles in DE diagnosis in the examination room before ophthalmologists examine the patient’s eyes using fluorescein in the consultation room with reference to the AI judgment, and this could become a possible realization of non-invasive TFOD by VK that we previously reported or AI-supported TFOD [21,22].

This study adopted the original VK data and BV data obtained from the patients’ eyes for DL. Compared to fluorescein, which can assess not only the dynamics of fluorescein-stained aqueous TF and fluorescein BU but also fluorescein-stained areas of CED over the entire corneal surface within the palpebral zone, the information obtainable through VK is somewhat limited, as it can only detect the disturbance of the MR associated with TF instability and CED in DE. In sADDE, MR disturbance is derived chiefly from more severe CED, and in DWDE and IEDE, the disturbance comes only from TF instability because of its sufficiency of tear volume and minimal involvement of CED, and such abnormalities are only detected by the MR disturbance [22]. Therefore, there are some intrinsic limitations for VK to detect corneal surface abnormalities in DE, even with the aid of AI. Considering those limitations in VK applied to the present study, the classification of DE via VK seems to be satisfactory.

As for the feasibility of the present AI-supported TFOD using VK being applied in the clinical setting, there are some important points that need to be considered. First, the TFOD pathway needs to be adopted in VK in the same manner in which it is conducted in fluorescein-supported TFOD using slit-lamp biomicroscopy. Second, verbal instruction to the patient of the required blink is essential, i.e., “gently close your eye”, “now rapid open your eye”, and “now keep your eye open” [5], as it greatly enhances the detection of the breakup pattern. Third, it is important that each patient be instructed to not use any eye drops for at least one hour prior to the test, as was done in this present study, as it makes for an accurate diagnosis of the DE subtype without any interference by the eye drops that the patient routinely uses. If those specific rules are strictly kept, the diagnosis of the DE subtype can effectively be performed with AI-supported TFOD using VK. However, in each examination before and after treatment, AI-supported TFOD using VK should be conducted at a similar time of day, i.e., either in the morning or in the afternoon, in consideration of the patient’s daily activities.

The limitation of this AI-supported TFOD using VK is that there is a hurdle to overcome for the differentiation of mild DE subtypes, including mild ADDE with mild CED, IEDE, and DWDE presenting RE of the BU [6,7], as those DE subtypes may show an MR disturbance that is too minor for AI to differentiate. However, mild ADDE is likely to manifest LB and mild CED, especially at the lower region of the cornea, which would be differentially diagnosed with IDDE presenting RB using the semantic segmentation method, which can together be detected by the CAM heat map [35]. In DWDE, BUPs other than SB, such as DB, LB with RE, and RB with RE, tend to take longer to appear and are followed by TF BU, leading to an MR disturbance after the eye is kept open, which sometimes takes more than 7 s to occur [7]. That is because non-invasive BU, i.e., full-thickness TF BU, takes a longer time to appear compared to fluorescein BU, merely corresponding to a thinning of the aqueous layer [11]. In this study, the AI algorithm was made by the images until 7 s after eye opening, and that might have been attributed to this limitation. However, semantic segmentation with the CAM heat map may be useful to detect the RE of the BU and diagnose DWDE properly. In addition, the possibility of AI-supported TFOD discovered through this study should be applied to the real world of DE to test its effectiveness in diagnosing the disease in a large number of DE patients in future studies. Moreover, the number of patients and eyes (243 eyes out of 243 DE cases) used in the current study is comparable with the sample size (350 eyes out of 178 subjects) used in a recently published report [25], which attempted to implement the classification of TF patterns suggested by us for specular microscopy images.

In conclusion, the findings in this study indicated the possibility that DL via VK with BV may be useful to DE diagnosis as a new non-invasive tool for DE classification. In future studies, that possibility would be enhanced by the combination of this method with semantic segmentation. Since some clue to the diagnosis of the DE subtype in the examination room could be possible via this present method, it will be of great value in the realization of shifting the diagnosis of DE from the consultation room to the examination room and before the accurate diagnosis using fluorescein in the consultation room.

## Figures and Tables

**Table 1:** Details of the patient data.

Data Conditions		Data Number
Number of eyes	All numbers	243
	Left	107
	Right	136
Sex	Males	23
	Females	220
Age	Mean	66.4
	SD	13.9

**Table 2:** Relationship between DE subtypes and FBUPs.

DE Subtype		FBUP
ADDE	sADDE	AB, partial AB
	m/mADDE	LB
DWDE		SB, DB, LB with RE, RB with RE
IEDE		RB

**Table 3:** Breakdown of the amount of training data and test data.

DE Subtypes	Training Data	Training Data (Augmented)	Test Data	FBUP	Training Data	Training Data (Augmented)	Test Data
sADDE	18	324	13	AB	7	126	5
				Partial AB	11	198	8
m/mADDE	44	792	29	LB	44	792	29
DWDE	51	918	33	SB	18	324	12
				DB	13	234	8
				LB with RE	10	180	6
				RB with RE	10	180	7
IEDE	33	594	22	RB	33	594	22
Total	146	2628	97	Total	146	2628	97

DE: dry eye; FBUP: fluorescein breakup pattern; sADDE: severe aqueous-deficient dry eye; AB: area break; m/mADDE: mild-to-moderate aqueous-deficient dry eye; LB: line break; SB: spot break; DB: dimple break; DWDE: decreased wettability dry eye; RE: rapid expansion; RB: random break; and IEDE: increased evaporation dry eye.

**Table 4:** Details of the DL model.

Layers	Details	Activation	Output Dimension
Input (video data)	Input	-	70 × 150 × 150 × 2
CNN Block1 (2D-CNN)	Conv2D	ReLU	75 × 75 × 16
	Conv2D	ReLU	75 × 75 × 16
	Batch Normalization	-	75 × 75 × 16
	Max Pooling 2D	-	75 × 75 × 16
	ResNet-D	ReLU	38 × 38 × 32
	Batch Normalization	-	38 × 38 × 32
	ResNet-D	ReLU	19 × 19 × 64
	Batch Normalization	-	19 × 19 × 64
	Dropout	ReLU	19 × 19 × 64
Time distribution	Time Distribution	-	70 × 19 × 19 × 64
CNN Block2 (3D-CNN)	3D-ResNet	ReLU	35 × 10 × 10 × 128
	Batch Normalization	-	35 × 10 × 10 × 128
	3D-ResNet	ReLU	18 × 5 × 5 × 256
	Batch Normalization	-	18 × 5 × 5 × 256
Global Max Pooling 3D	Global Max Pooling 3D	-	256
Prediction	Dense	Softmax	3 or 2

## Data Availability

The data that support the findings in this study are available on reasonable request from the corresponding author.

## Supplementary Materials

The following supporting information can be downloaded at https://www.mdpi.com/article/10.3390/diagnostics14010052/s1. A total of 8 typical example images (150 original images acquired by VK) of 8 types of BUPs can be downloaded, including dimple break, line break with rapid expansion, random break with rapid expansion, and spot break for DWDE, as well as random break for IEDE, line break for mild-to-moderate ADDE, and area break and partial area break for severe ADDE.

## Abbreviations

AB	area break
ADDE	aqueous-deficient dry eye
AI	artificial intelligence
BV	blur value
DB	dimple break
BU	breakup
BUP	BU pattern
CAM	class activation map
CED	corneal epithelial damage
CNN	convolutional neural network
D	dimension
DE	dry eye
DEWSII	Dry Eye Workshop II
DL	deep learning
DWDE	decreased wettability DE
FBUP	fluorescein BUP
IEDE	increased evaporation DE
LB	line break
m/mADDE	mild-to-moderate ADDE
MR	Meyer-ring
OS	ocular surface
OSDs	OS disorders
pAB	partial AB
RB	random break
RE	rapid expansion
sADDE	severe ADDE
SB	spot break
SD	standard deviation
TF	tear film
TFOD	TF-oriented diagnosis
TFOT	TF-oriented therapy
VK	videokeratography
