# Supplementary figures and images for: Dry Eye Subtype Classification Using Videokeratography and Deep Learning (part 2 of 2)
Source: Diagnostics (Basel). 2023 Dec 26;14(1):52. doi: 10.3390/diagnostics14010052 (PMC10802766; doi:10.3390/diagnostics14010052)

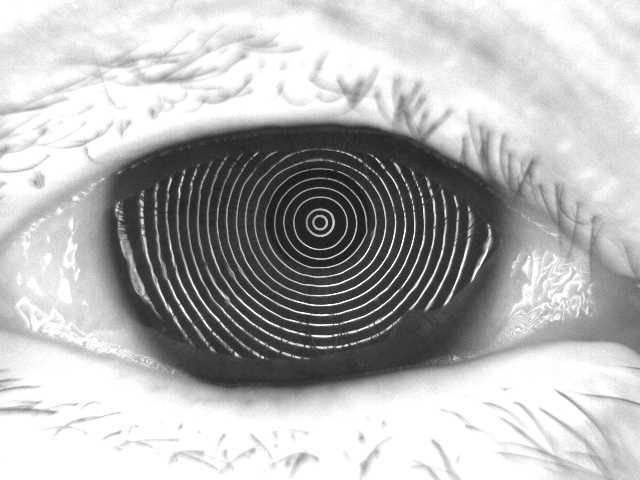

Supplement: Supplementary file 1 [file diagnostics-14-00052-s001.zip › Decreased wettability DE (DWDE)/Dimple break/0100.jpeg]

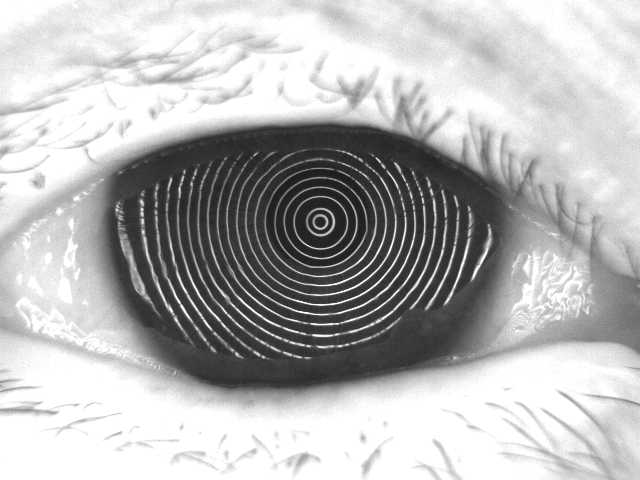

Supplement: Supplementary file 1 [file diagnostics-14-00052-s001.zip › Decreased wettability DE (DWDE)/Dimple break/0101.jpeg]

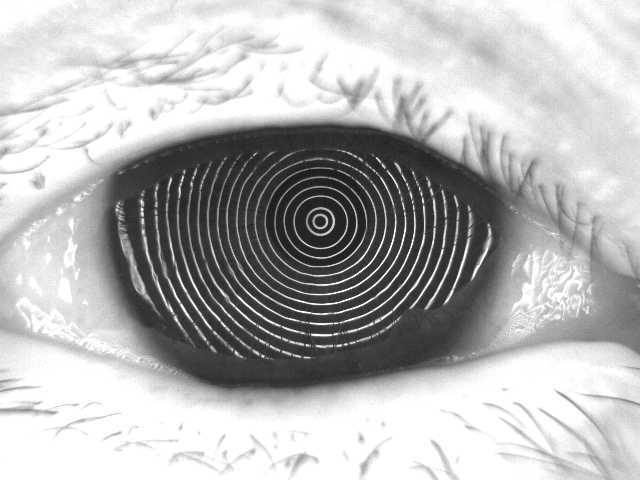

Supplement: Supplementary file 1 [file diagnostics-14-00052-s001.zip › Decreased wettability DE (DWDE)/Dimple break/0102.jpeg]

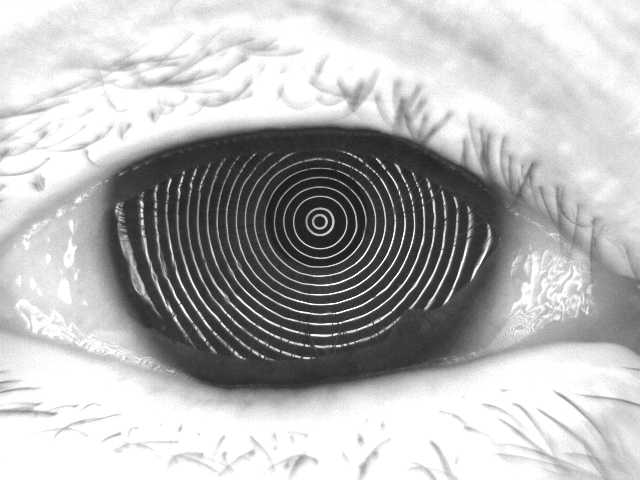

Supplement: Supplementary file 1 [file diagnostics-14-00052-s001.zip › Decreased wettability DE (DWDE)/Dimple break/0103.jpeg]

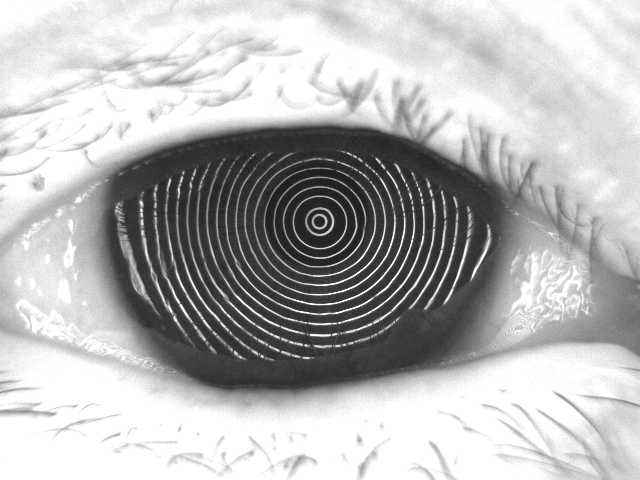

Supplement: Supplementary file 1 [file diagnostics-14-00052-s001.zip › Decreased wettability DE (DWDE)/Dimple break/0104.jpeg]

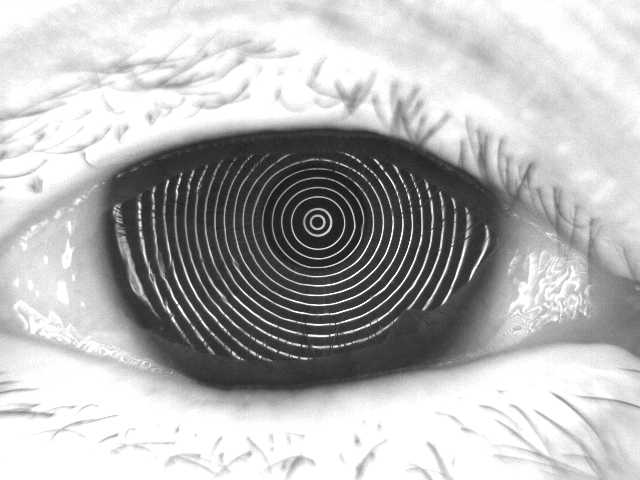

Supplement: Supplementary file 1 [file diagnostics-14-00052-s001.zip › Decreased wettability DE (DWDE)/Dimple break/0105.jpeg]

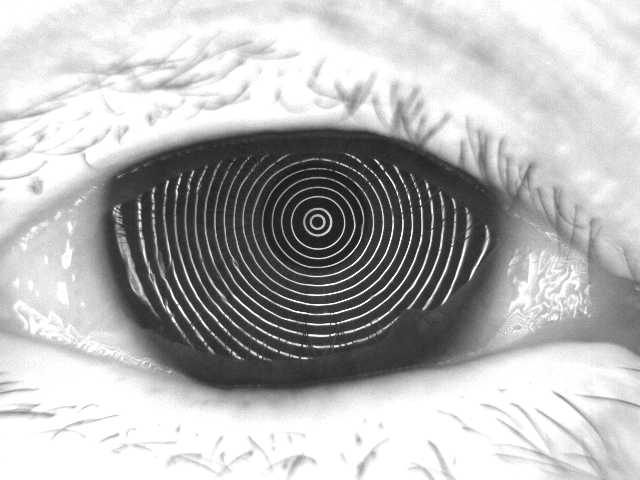

Supplement: Supplementary file 1 [file diagnostics-14-00052-s001.zip › Decreased wettability DE (DWDE)/Dimple break/0106.jpeg]

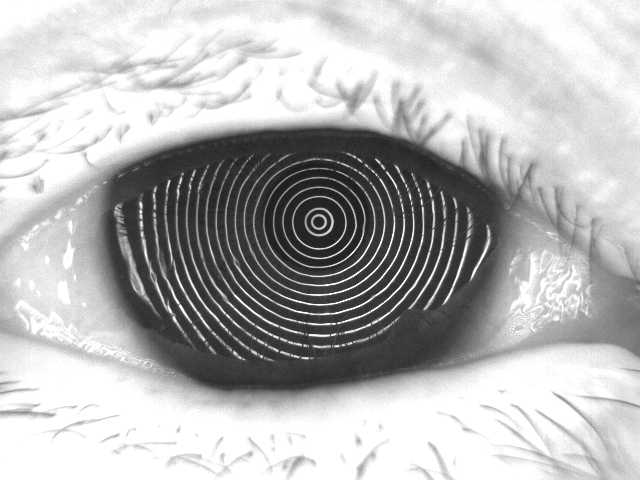

Supplement: Supplementary file 1 [file diagnostics-14-00052-s001.zip › Decreased wettability DE (DWDE)/Dimple break/0107.jpeg]

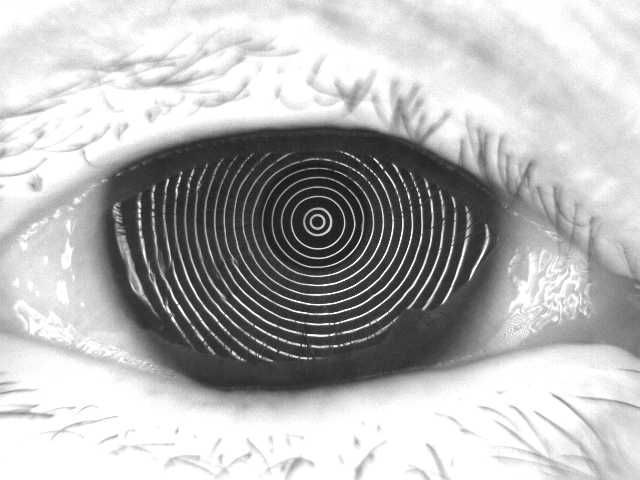

Supplement: Supplementary file 1 [file diagnostics-14-00052-s001.zip › Decreased wettability DE (DWDE)/Dimple break/0108.jpeg]

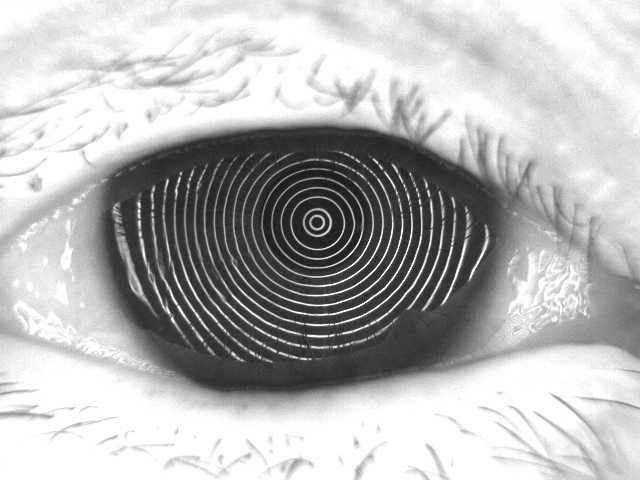

Supplement: Supplementary file 1 [file diagnostics-14-00052-s001.zip › Decreased wettability DE (DWDE)/Dimple break/0109.jpeg]

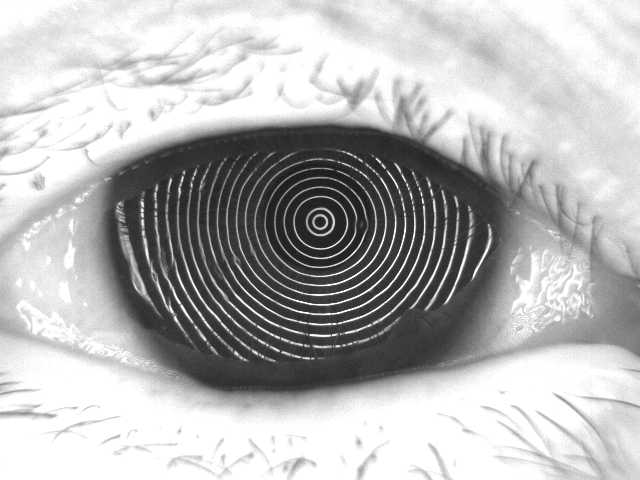

Supplement: Supplementary file 1 [file diagnostics-14-00052-s001.zip › Decreased wettability DE (DWDE)/Dimple break/0110.jpeg]

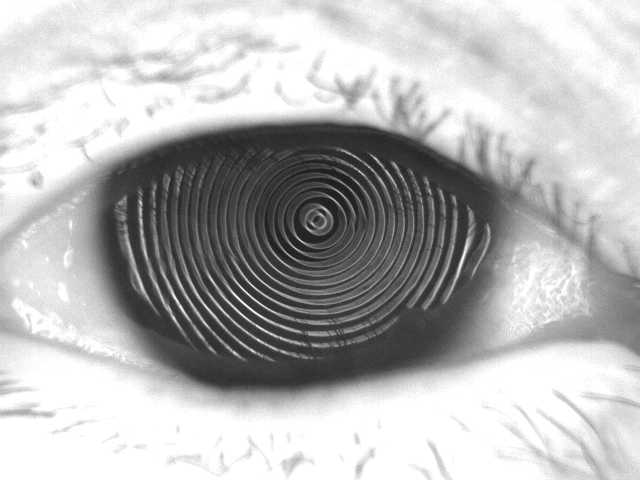

Supplement: Supplementary file 1 [file diagnostics-14-00052-s001.zip › Decreased wettability DE (DWDE)/Dimple break/0111.jpeg]

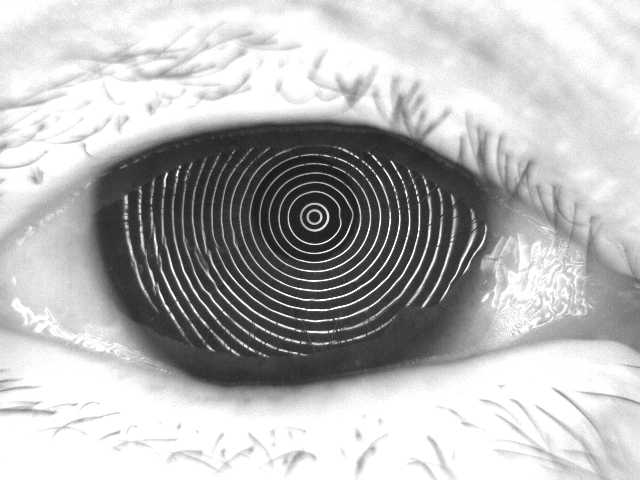

Supplement: Supplementary file 1 [file diagnostics-14-00052-s001.zip › Decreased wettability DE (DWDE)/Dimple break/0112.jpeg]

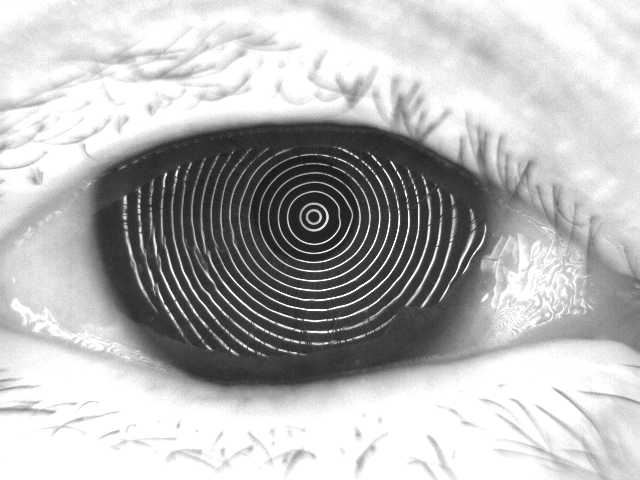

Supplement: Supplementary file 1 [file diagnostics-14-00052-s001.zip › Decreased wettability DE (DWDE)/Dimple break/0113.jpeg]

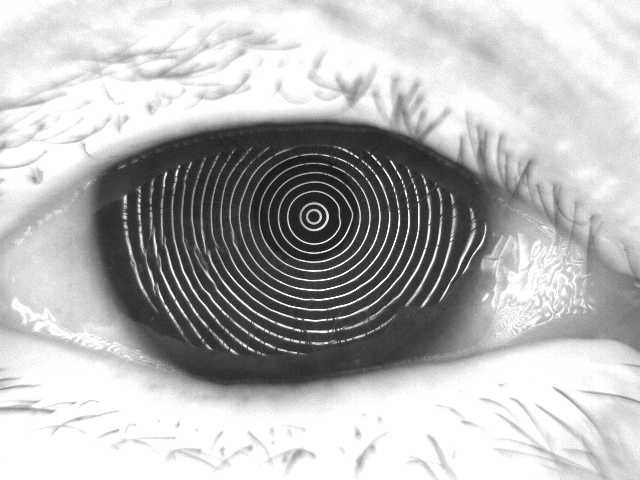

Supplement: Supplementary file 1 [file diagnostics-14-00052-s001.zip › Decreased wettability DE (DWDE)/Dimple break/0114.jpeg]

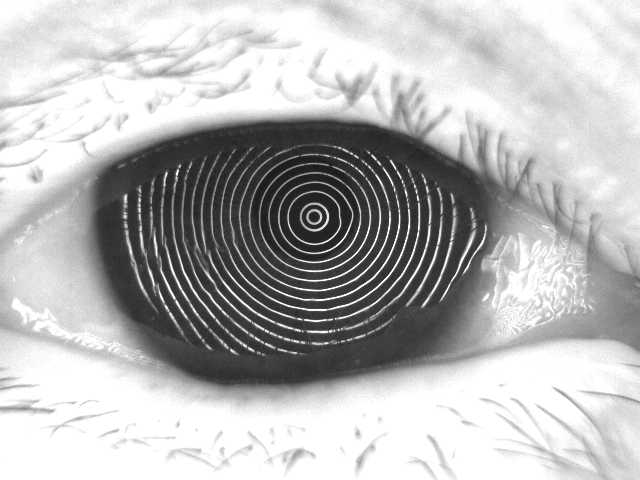

Supplement: Supplementary file 1 [file diagnostics-14-00052-s001.zip › Decreased wettability DE (DWDE)/Dimple break/0115.jpeg]

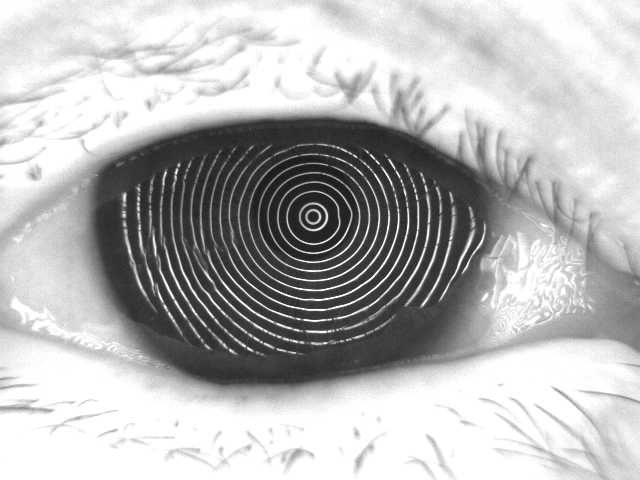

Supplement: Supplementary file 1 [file diagnostics-14-00052-s001.zip › Decreased wettability DE (DWDE)/Dimple break/0116.jpeg]

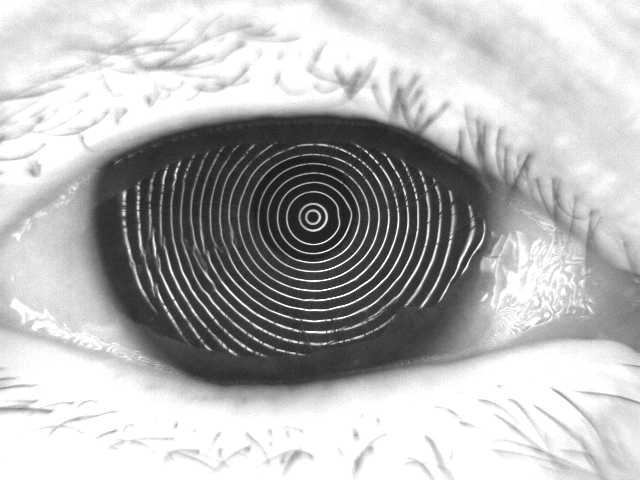

Supplement: Supplementary file 1 [file diagnostics-14-00052-s001.zip › Decreased wettability DE (DWDE)/Dimple break/0117.jpeg]

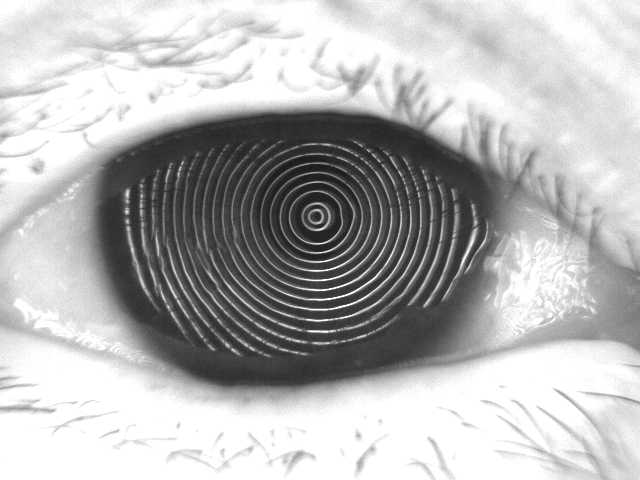

Supplement: Supplementary file 1 [file diagnostics-14-00052-s001.zip › Decreased wettability DE (DWDE)/Dimple break/0118.jpeg]

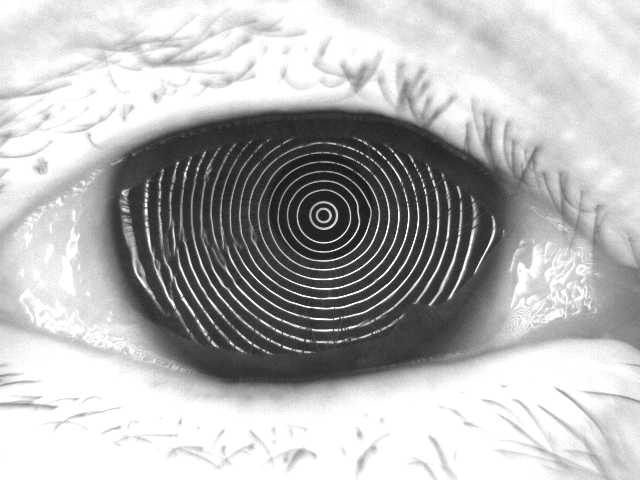

Supplement: Supplementary file 1 [file diagnostics-14-00052-s001.zip › Decreased wettability DE (DWDE)/Dimple break/0119.jpeg]

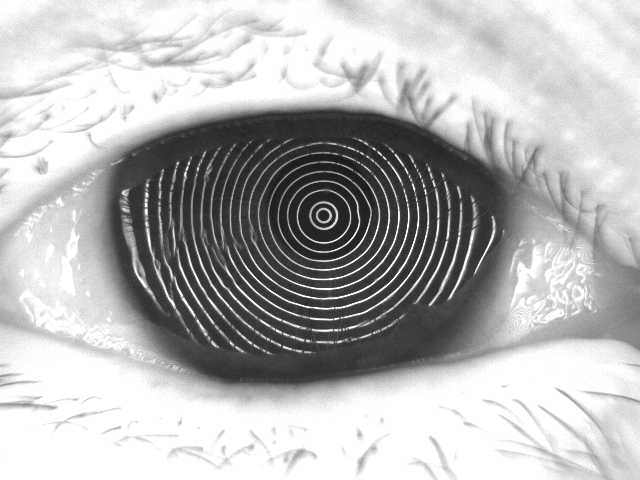

Supplement: Supplementary file 1 [file diagnostics-14-00052-s001.zip › Decreased wettability DE (DWDE)/Dimple break/0120.jpeg]

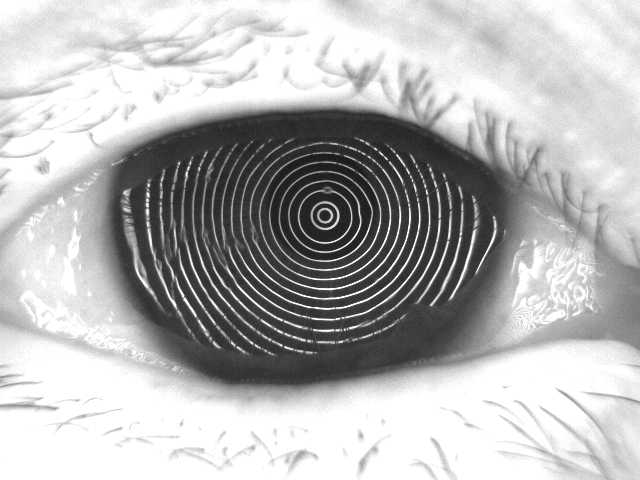

Supplement: Supplementary file 1 [file diagnostics-14-00052-s001.zip › Decreased wettability DE (DWDE)/Dimple break/0121.jpeg]

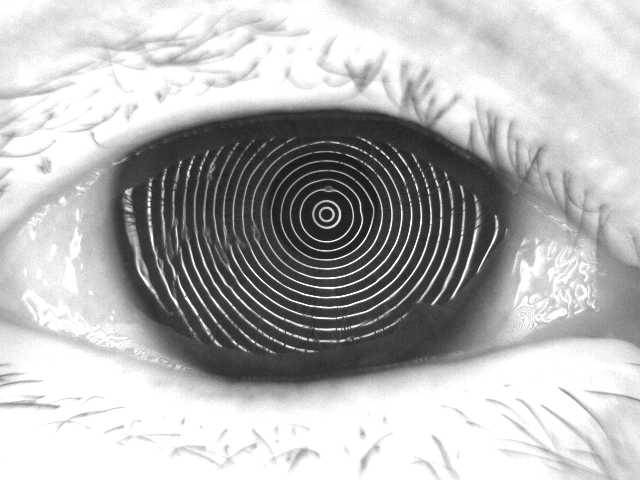

Supplement: Supplementary file 1 [file diagnostics-14-00052-s001.zip › Decreased wettability DE (DWDE)/Dimple break/0122.jpeg]

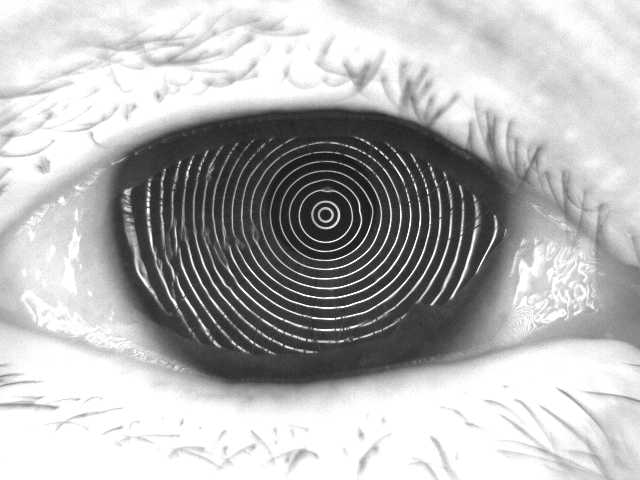

Supplement: Supplementary file 1 [file diagnostics-14-00052-s001.zip › Decreased wettability DE (DWDE)/Dimple break/0123.jpeg]

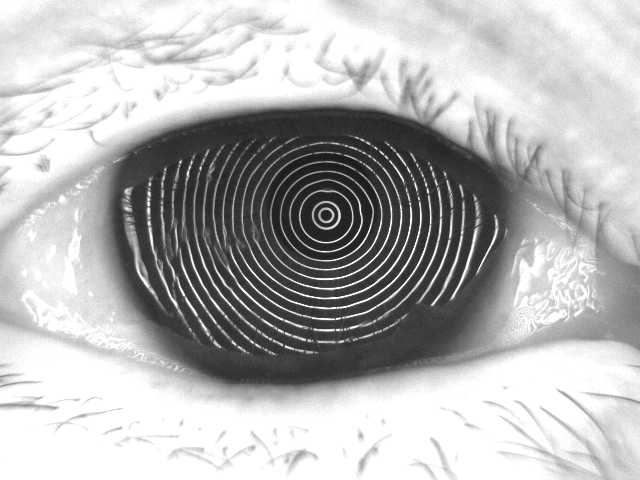

Supplement: Supplementary file 1 [file diagnostics-14-00052-s001.zip › Decreased wettability DE (DWDE)/Dimple break/0124.jpeg]

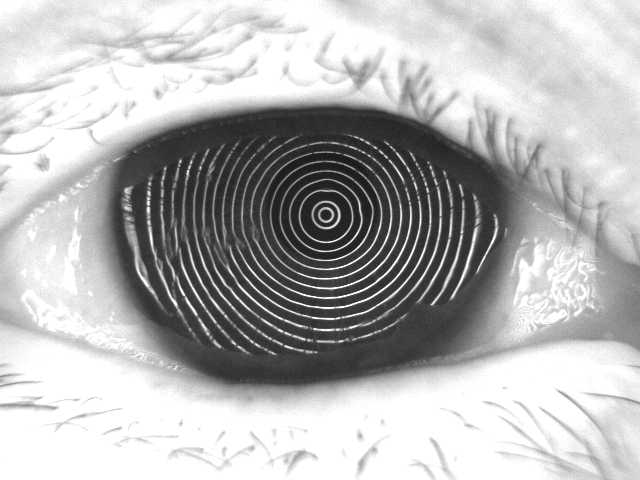

Supplement: Supplementary file 1 [file diagnostics-14-00052-s001.zip › Decreased wettability DE (DWDE)/Dimple break/0125.jpeg]

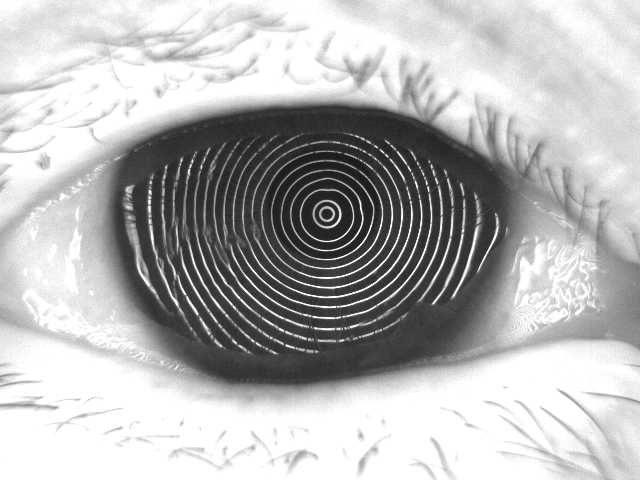

Supplement: Supplementary file 1 [file diagnostics-14-00052-s001.zip › Decreased wettability DE (DWDE)/Dimple break/0126.jpeg]

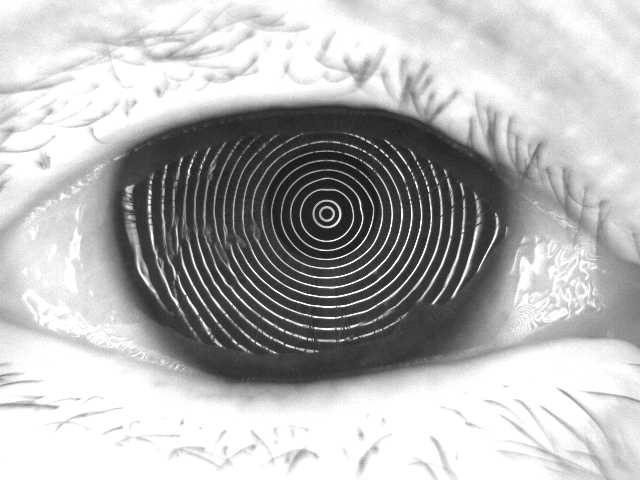

Supplement: Supplementary file 1 [file diagnostics-14-00052-s001.zip › Decreased wettability DE (DWDE)/Dimple break/0127.jpeg]

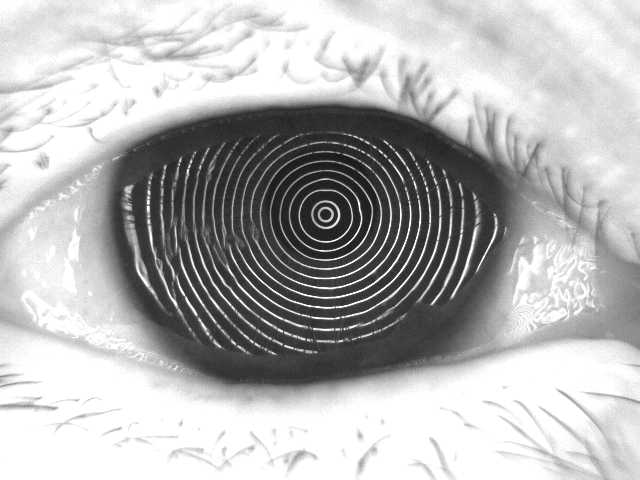

Supplement: Supplementary file 1 [file diagnostics-14-00052-s001.zip › Decreased wettability DE (DWDE)/Dimple break/0128.jpeg]

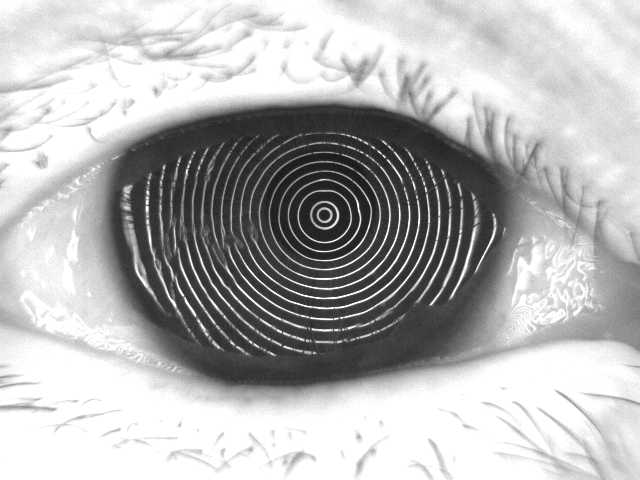

Supplement: Supplementary file 1 [file diagnostics-14-00052-s001.zip › Decreased wettability DE (DWDE)/Dimple break/0129.jpeg]

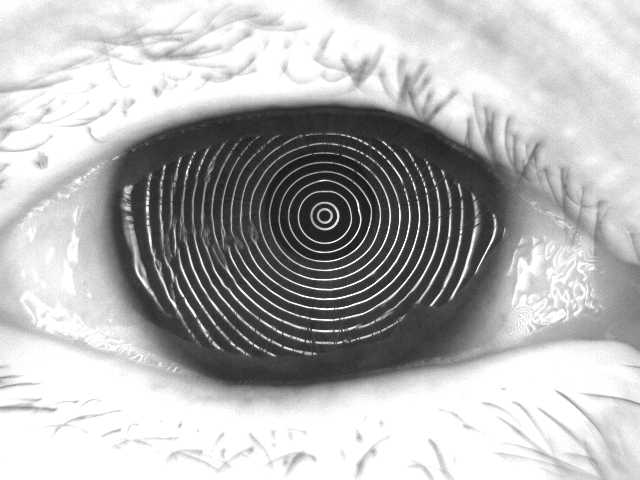

Supplement: Supplementary file 1 [file diagnostics-14-00052-s001.zip › Decreased wettability DE (DWDE)/Dimple break/0130.jpeg]

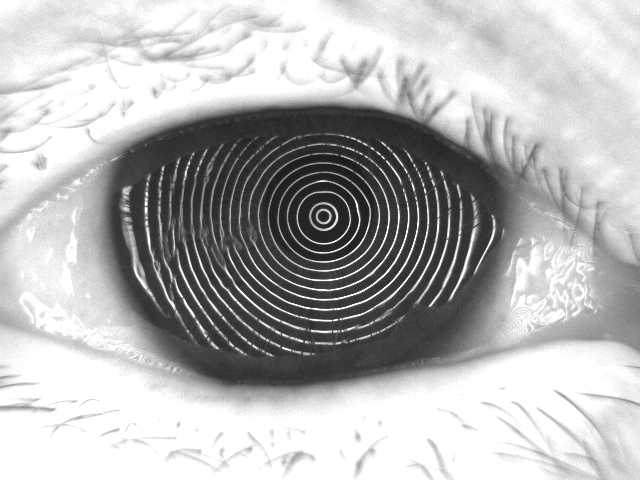

Supplement: Supplementary file 1 [file diagnostics-14-00052-s001.zip › Decreased wettability DE (DWDE)/Dimple break/0131.jpeg]

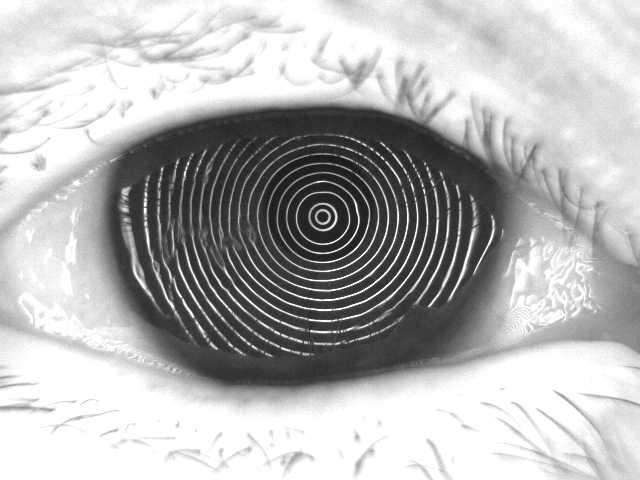

Supplement: Supplementary file 1 [file diagnostics-14-00052-s001.zip › Decreased wettability DE (DWDE)/Dimple break/0132.jpeg]

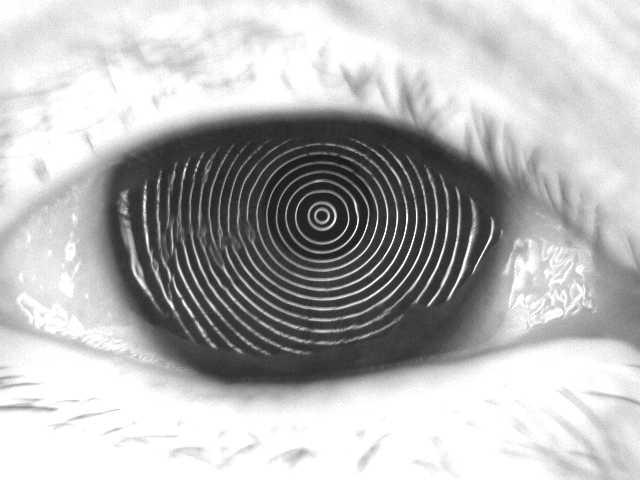

Supplement: Supplementary file 1 [file diagnostics-14-00052-s001.zip › Decreased wettability DE (DWDE)/Dimple break/0133.jpeg]

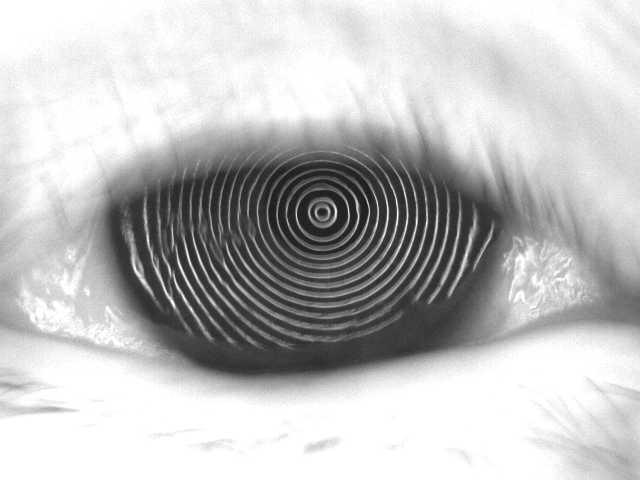

Supplement: Supplementary file 1 [file diagnostics-14-00052-s001.zip › Decreased wettability DE (DWDE)/Dimple break/0134.jpeg]

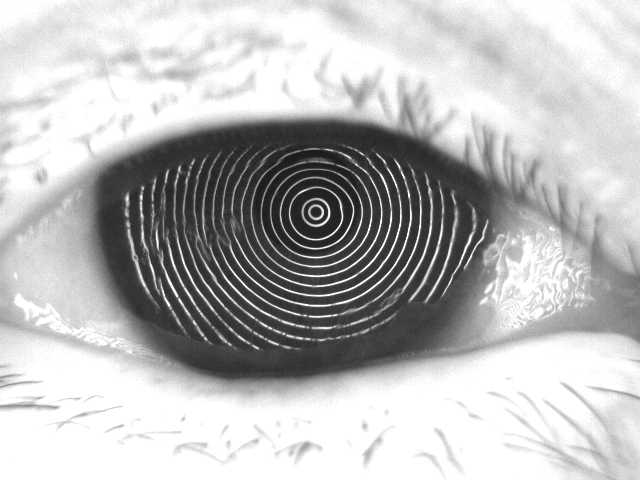

Supplement: Supplementary file 1 [file diagnostics-14-00052-s001.zip › Decreased wettability DE (DWDE)/Dimple break/0135.jpeg]

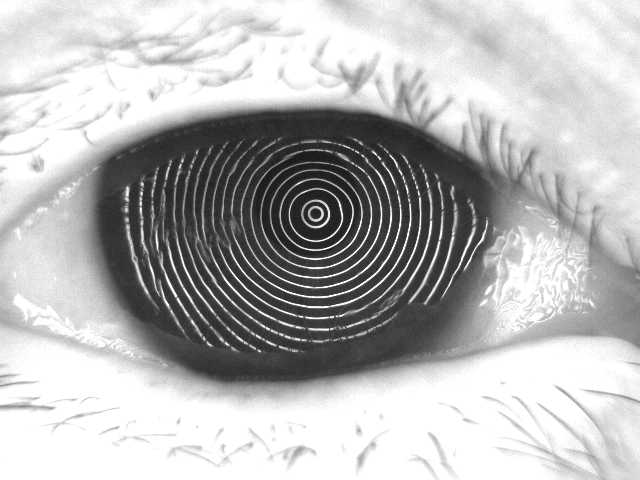

Supplement: Supplementary file 1 [file diagnostics-14-00052-s001.zip › Decreased wettability DE (DWDE)/Dimple break/0136.jpeg]

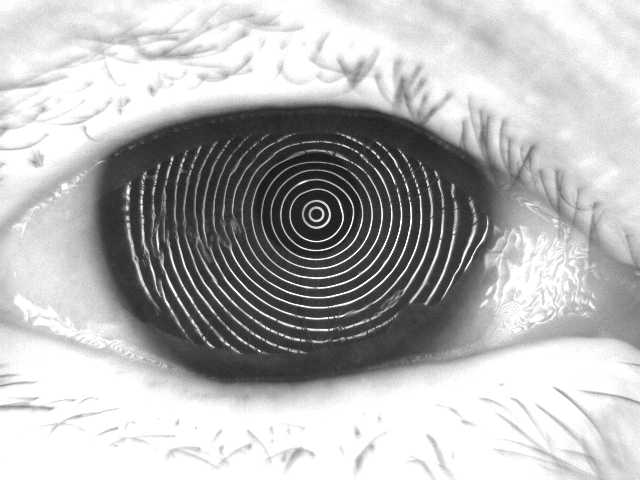

Supplement: Supplementary file 1 [file diagnostics-14-00052-s001.zip › Decreased wettability DE (DWDE)/Dimple break/0137.jpeg]

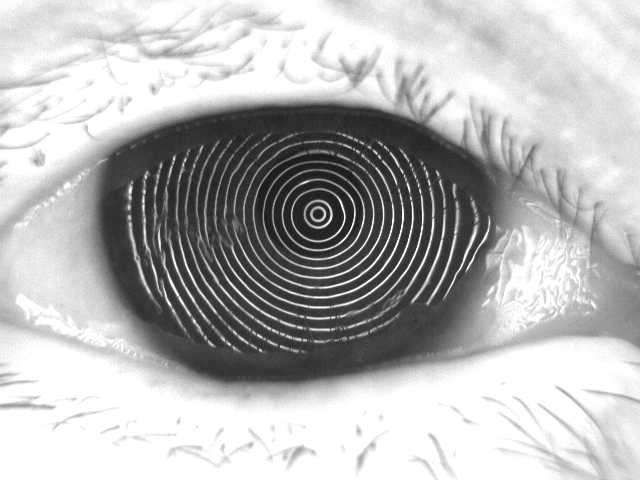

Supplement: Supplementary file 1 [file diagnostics-14-00052-s001.zip › Decreased wettability DE (DWDE)/Dimple break/0138.jpeg]

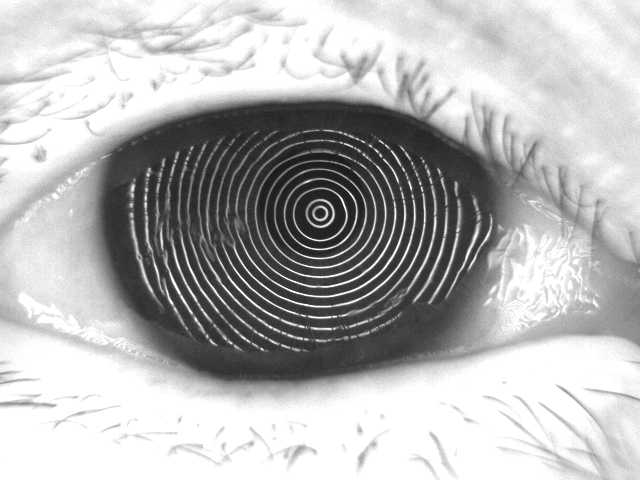

Supplement: Supplementary file 1 [file diagnostics-14-00052-s001.zip › Decreased wettability DE (DWDE)/Dimple break/0139.jpeg]

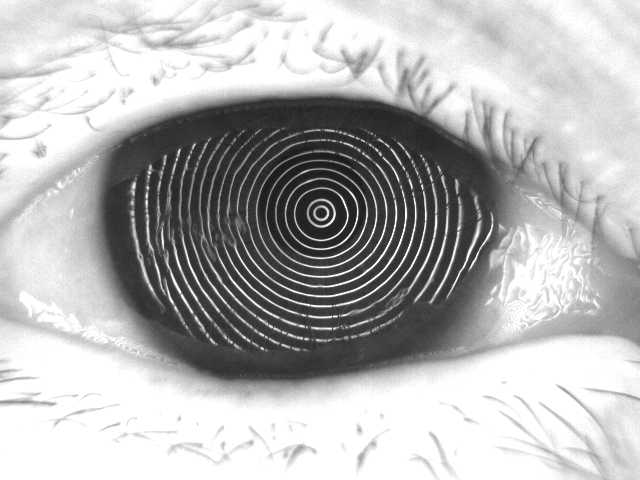

Supplement: Supplementary file 1 [file diagnostics-14-00052-s001.zip › Decreased wettability DE (DWDE)/Dimple break/0140.jpeg]

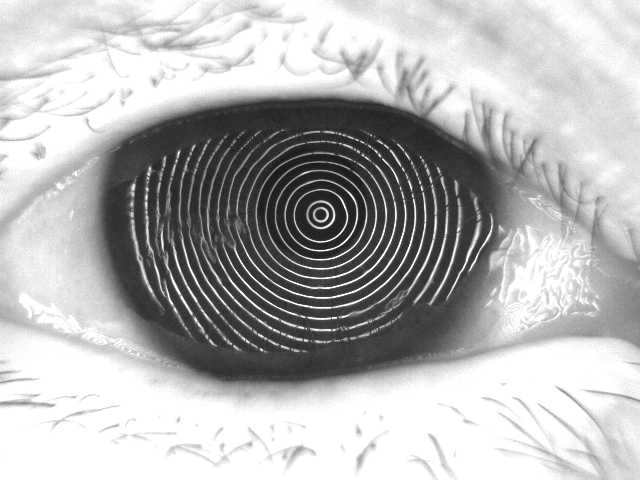

Supplement: Supplementary file 1 [file diagnostics-14-00052-s001.zip › Decreased wettability DE (DWDE)/Dimple break/0141.jpeg]

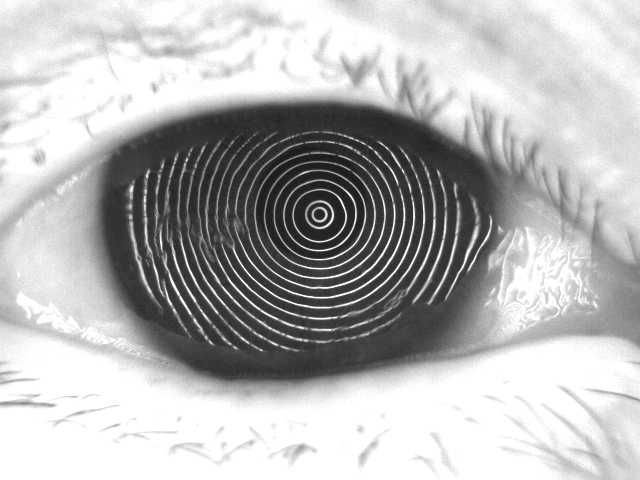

Supplement: Supplementary file 1 [file diagnostics-14-00052-s001.zip › Decreased wettability DE (DWDE)/Dimple break/0142.jpeg]

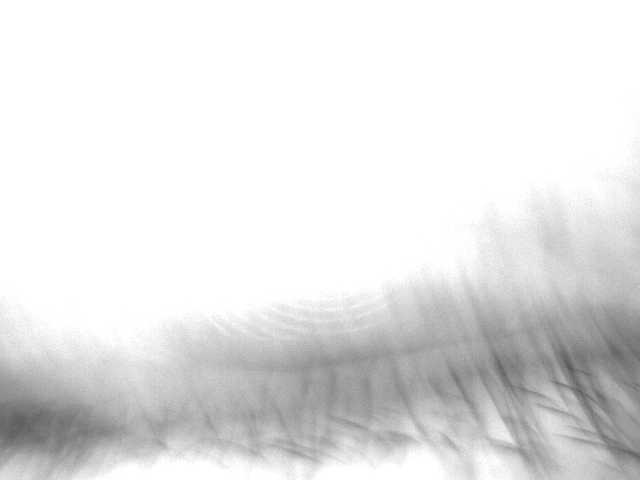

Supplement: Supplementary file 1 [file diagnostics-14-00052-s001.zip › Decreased wettability DE (DWDE)/Dimple break/0143.jpeg]

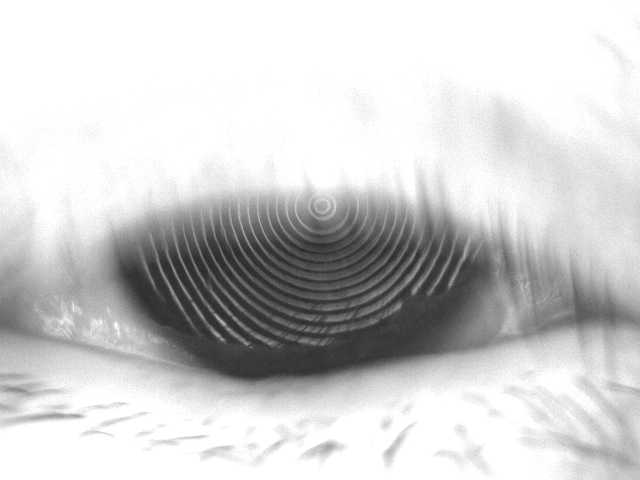

Supplement: Supplementary file 1 [file diagnostics-14-00052-s001.zip › Decreased wettability DE (DWDE)/Dimple break/0144.jpeg]

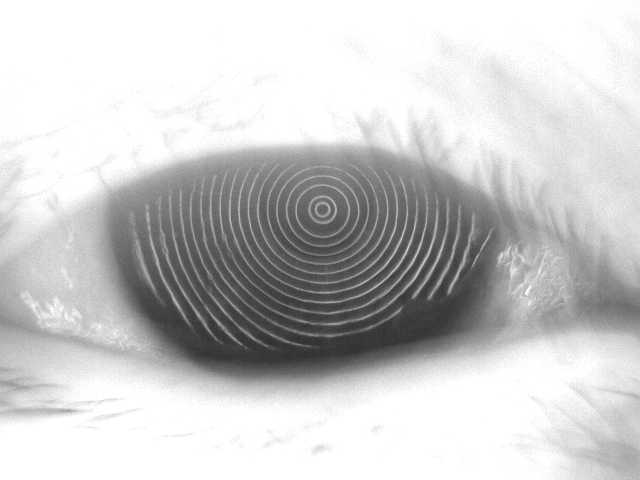

Supplement: Supplementary file 1 [file diagnostics-14-00052-s001.zip › Decreased wettability DE (DWDE)/Dimple break/0145.jpeg]

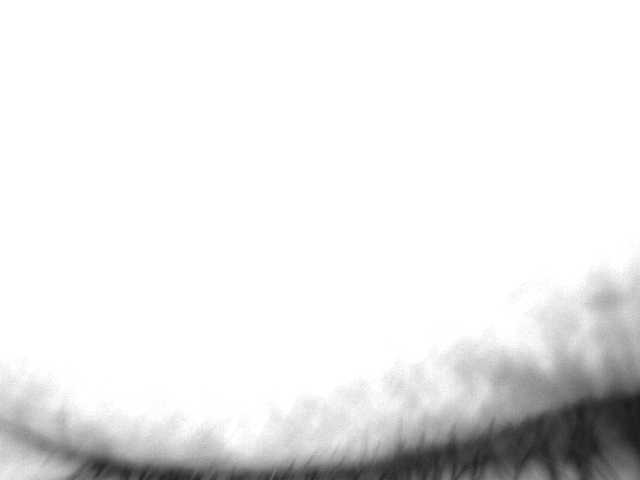

Supplement: Supplementary file 1 [file diagnostics-14-00052-s001.zip › Decreased wettability DE (DWDE)/Dimple break/0146.jpeg]

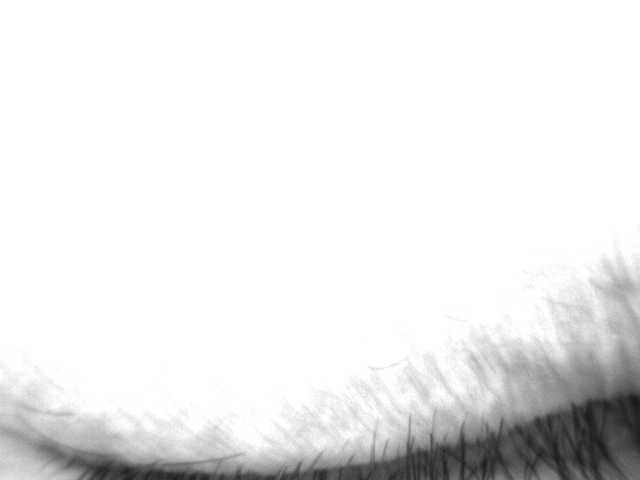

Supplement: Supplementary file 1 [file diagnostics-14-00052-s001.zip › Decreased wettability DE (DWDE)/Dimple break/0147.jpeg]

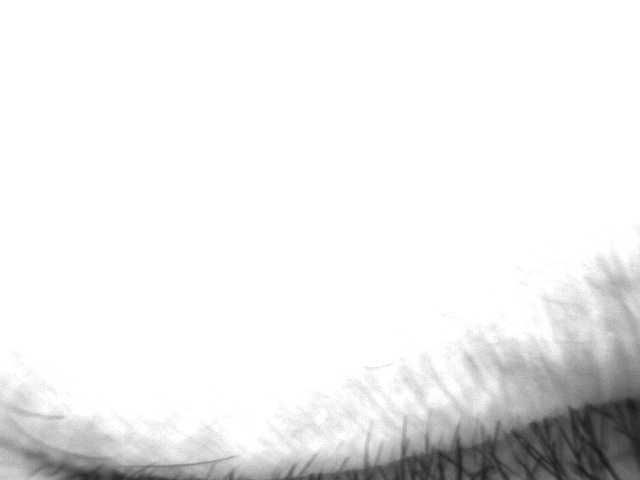

Supplement: Supplementary file 1 [file diagnostics-14-00052-s001.zip › Decreased wettability DE (DWDE)/Dimple break/0148.jpeg]

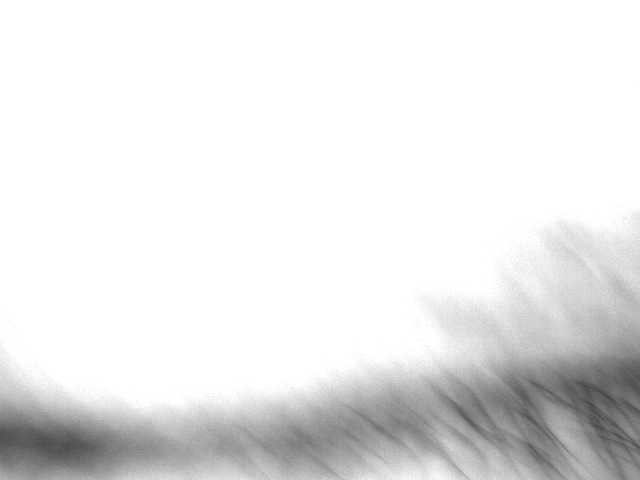

Supplement: Supplementary file 1 [file diagnostics-14-00052-s001.zip › Decreased wettability DE (DWDE)/Dimple break/0149.jpeg]

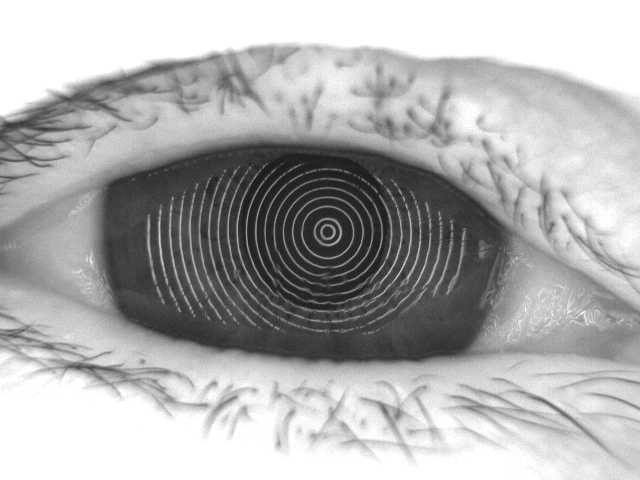

Supplement: Supplementary file 1 [file diagnostics-14-00052-s001.zip › Decreased wettability DE (DWDE)/Line break with rapid expansion/0000.jpeg]

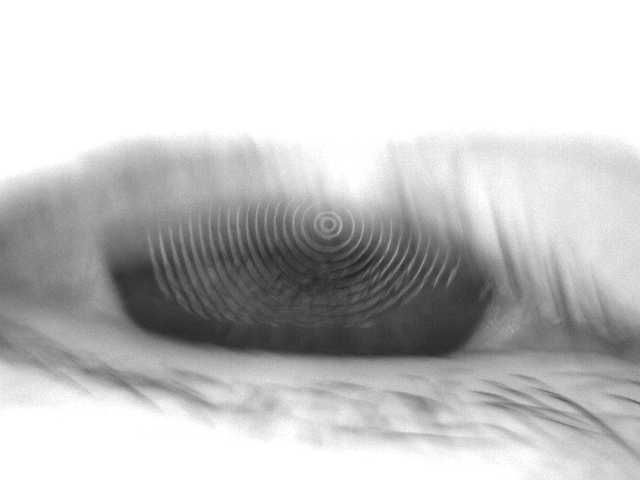

Supplement: Supplementary file 1 [file diagnostics-14-00052-s001.zip › Decreased wettability DE (DWDE)/Line break with rapid expansion/0001.jpeg]

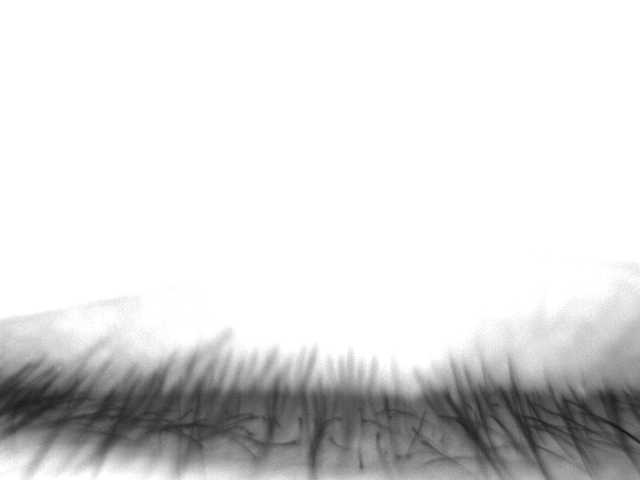

Supplement: Supplementary file 1 [file diagnostics-14-00052-s001.zip › Decreased wettability DE (DWDE)/Line break with rapid expansion/0002.jpeg]

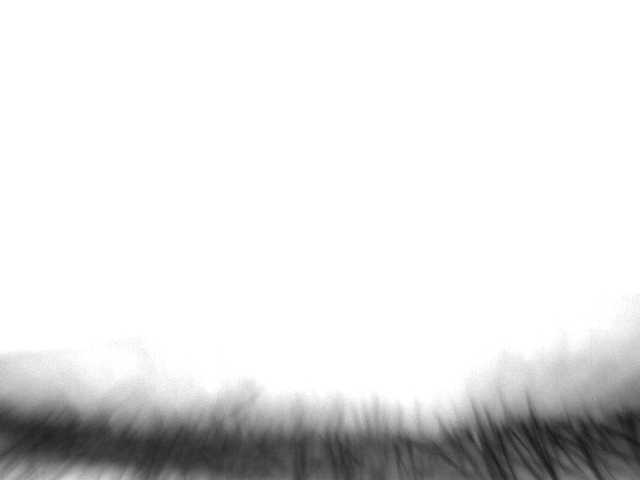

Supplement: Supplementary file 1 [file diagnostics-14-00052-s001.zip › Decreased wettability DE (DWDE)/Line break with rapid expansion/0003.jpeg]

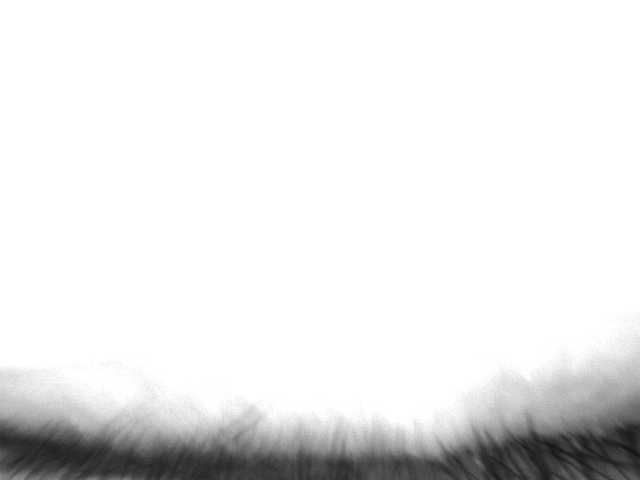

Supplement: Supplementary file 1 [file diagnostics-14-00052-s001.zip › Decreased wettability DE (DWDE)/Line break with rapid expansion/0004.jpeg]

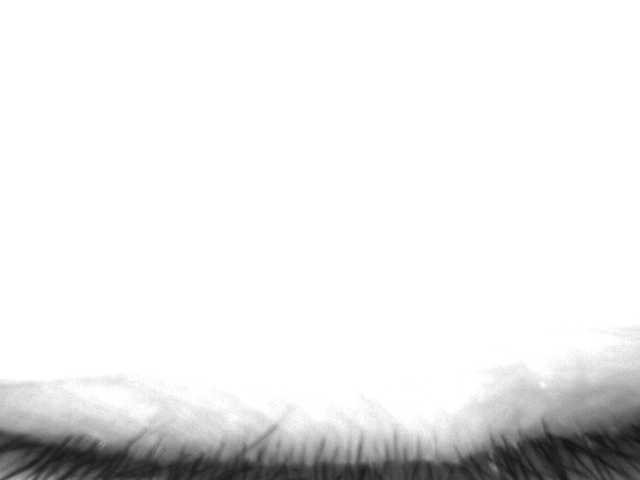

Supplement: Supplementary file 1 [file diagnostics-14-00052-s001.zip › Decreased wettability DE (DWDE)/Line break with rapid expansion/0005.jpeg]

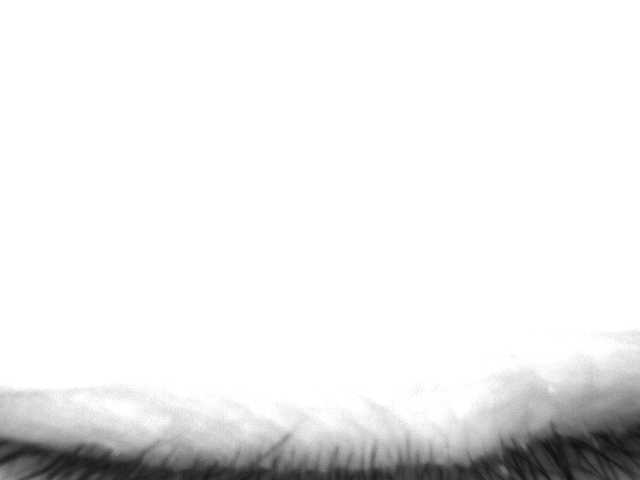

Supplement: Supplementary file 1 [file diagnostics-14-00052-s001.zip › Decreased wettability DE (DWDE)/Line break with rapid expansion/0006.jpeg]

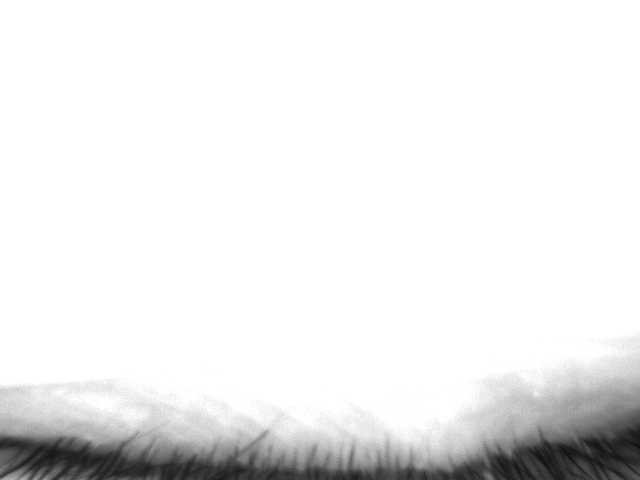

Supplement: Supplementary file 1 [file diagnostics-14-00052-s001.zip › Decreased wettability DE (DWDE)/Line break with rapid expansion/0007.jpeg]

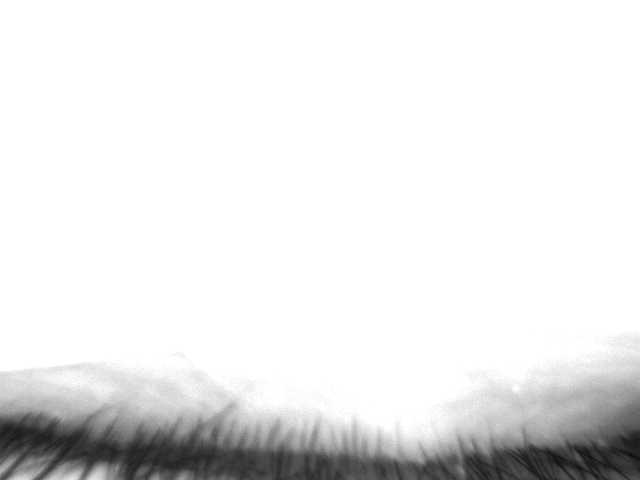

Supplement: Supplementary file 1 [file diagnostics-14-00052-s001.zip › Decreased wettability DE (DWDE)/Line break with rapid expansion/0008.jpeg]

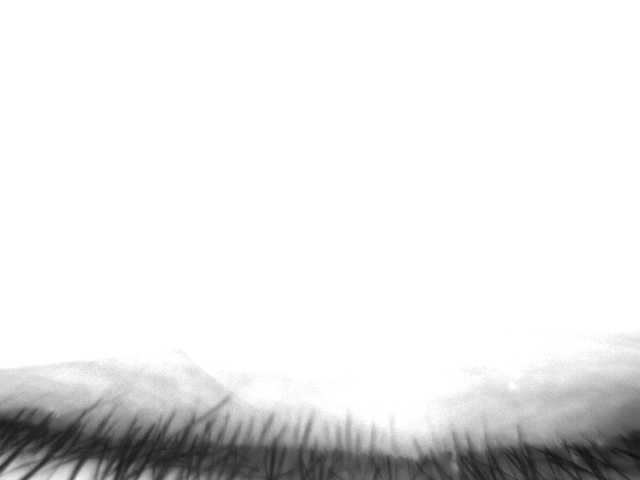

Supplement: Supplementary file 1 [file diagnostics-14-00052-s001.zip › Decreased wettability DE (DWDE)/Line break with rapid expansion/0009.jpeg]

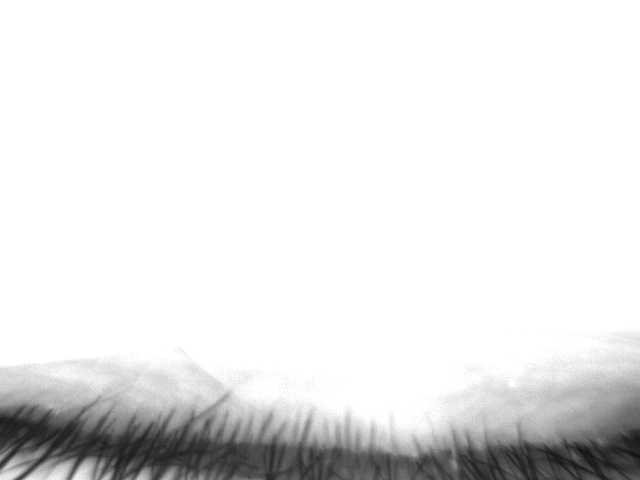

Supplement: Supplementary file 1 [file diagnostics-14-00052-s001.zip › Decreased wettability DE (DWDE)/Line break with rapid expansion/0010.jpeg]

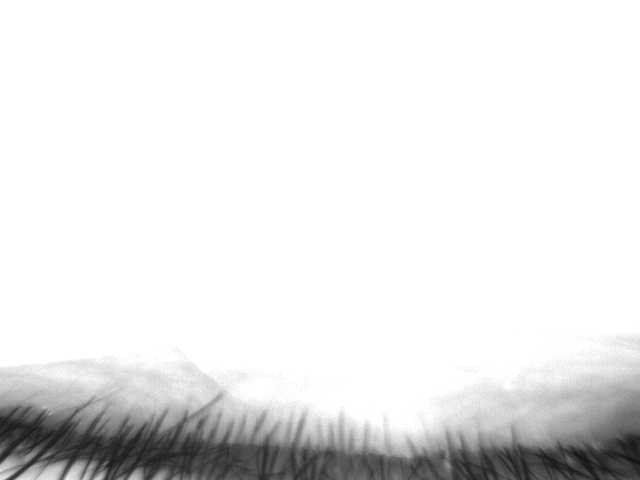

Supplement: Supplementary file 1 [file diagnostics-14-00052-s001.zip › Decreased wettability DE (DWDE)/Line break with rapid expansion/0011.jpeg]

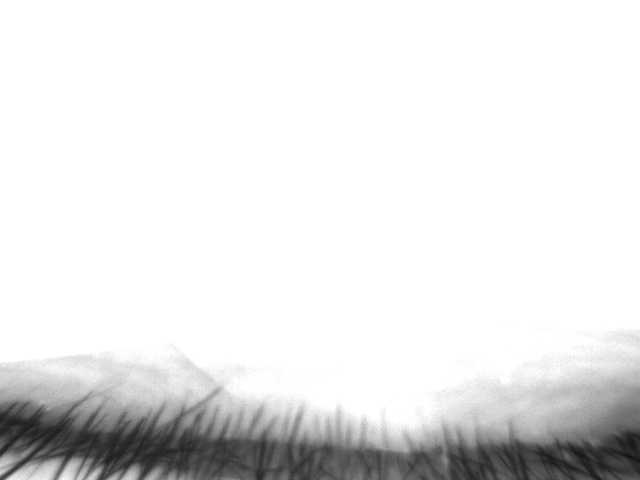

Supplement: Supplementary file 1 [file diagnostics-14-00052-s001.zip › Decreased wettability DE (DWDE)/Line break with rapid expansion/0012.jpeg]

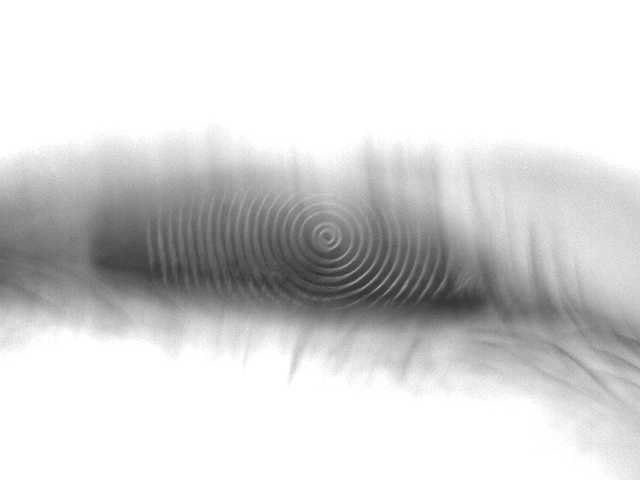

Supplement: Supplementary file 1 [file diagnostics-14-00052-s001.zip › Decreased wettability DE (DWDE)/Line break with rapid expansion/0013.jpeg]

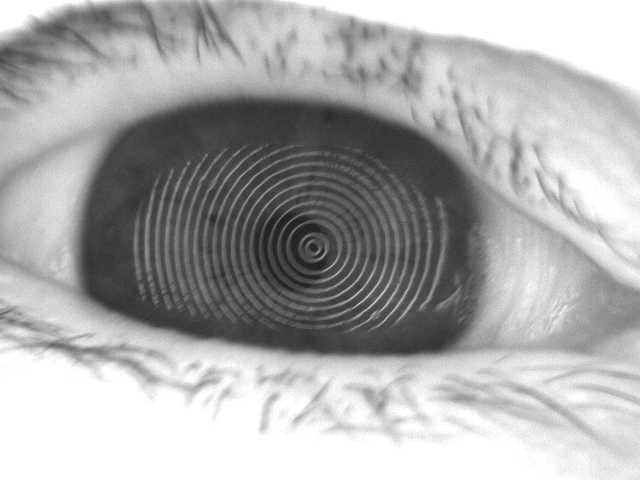

Supplement: Supplementary file 1 [file diagnostics-14-00052-s001.zip › Decreased wettability DE (DWDE)/Line break with rapid expansion/0014.jpeg]

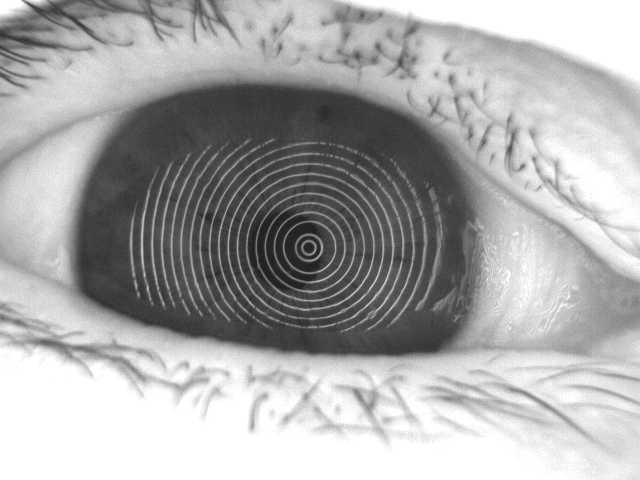

Supplement: Supplementary file 1 [file diagnostics-14-00052-s001.zip › Decreased wettability DE (DWDE)/Line break with rapid expansion/0015.jpeg]

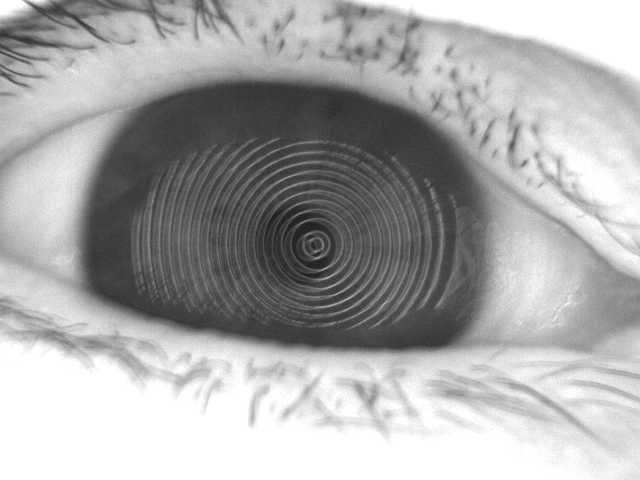

Supplement: Supplementary file 1 [file diagnostics-14-00052-s001.zip › Decreased wettability DE (DWDE)/Line break with rapid expansion/0016.jpeg]

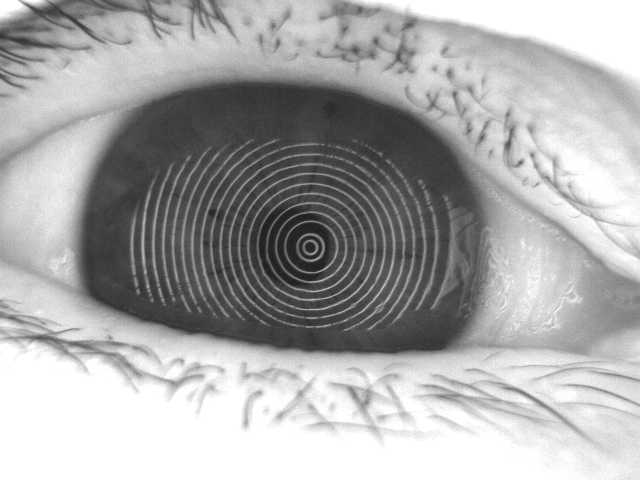

Supplement: Supplementary file 1 [file diagnostics-14-00052-s001.zip › Decreased wettability DE (DWDE)/Line break with rapid expansion/0017.jpeg]

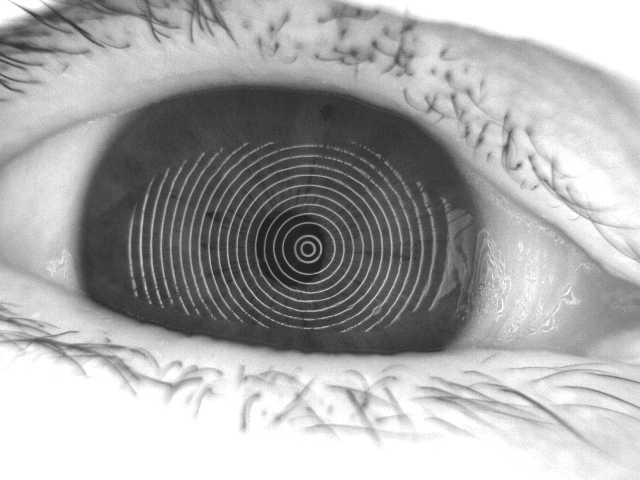

Supplement: Supplementary file 1 [file diagnostics-14-00052-s001.zip › Decreased wettability DE (DWDE)/Line break with rapid expansion/0018.jpeg]

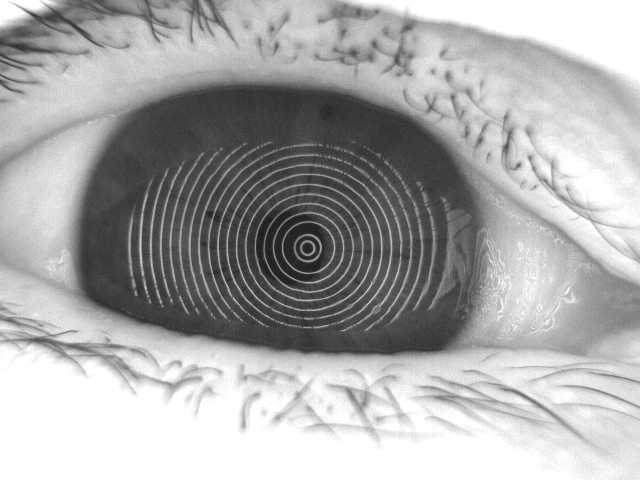

Supplement: Supplementary file 1 [file diagnostics-14-00052-s001.zip › Decreased wettability DE (DWDE)/Line break with rapid expansion/0019.jpeg]

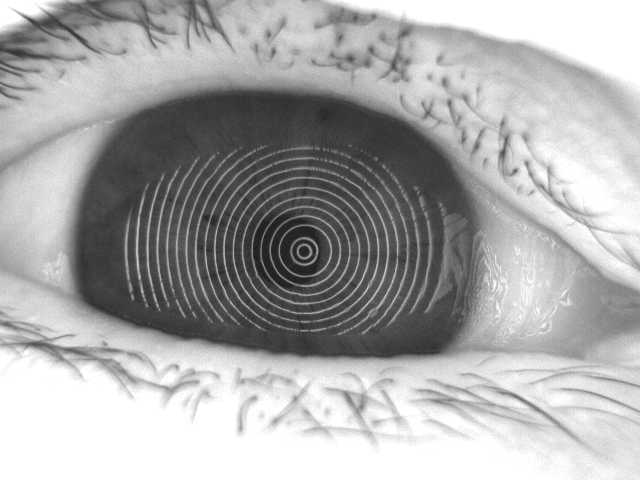

Supplement: Supplementary file 1 [file diagnostics-14-00052-s001.zip › Decreased wettability DE (DWDE)/Line break with rapid expansion/0020.jpeg]

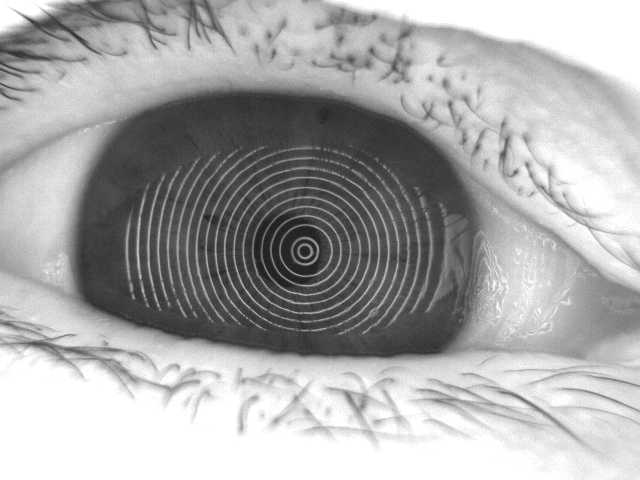

Supplement: Supplementary file 1 [file diagnostics-14-00052-s001.zip › Decreased wettability DE (DWDE)/Line break with rapid expansion/0021.jpeg]

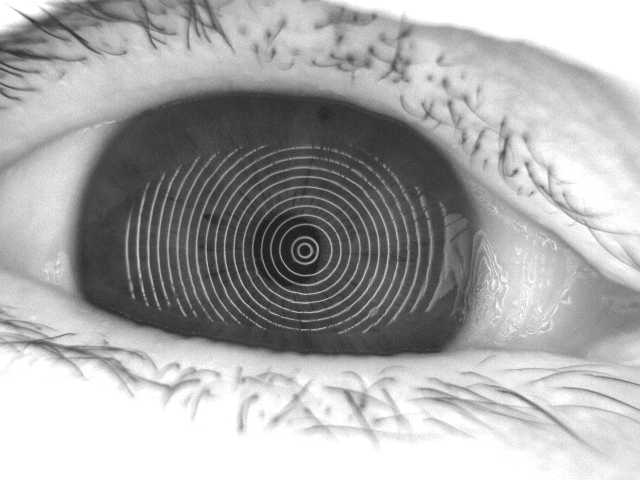

Supplement: Supplementary file 1 [file diagnostics-14-00052-s001.zip › Decreased wettability DE (DWDE)/Line break with rapid expansion/0022.jpeg]

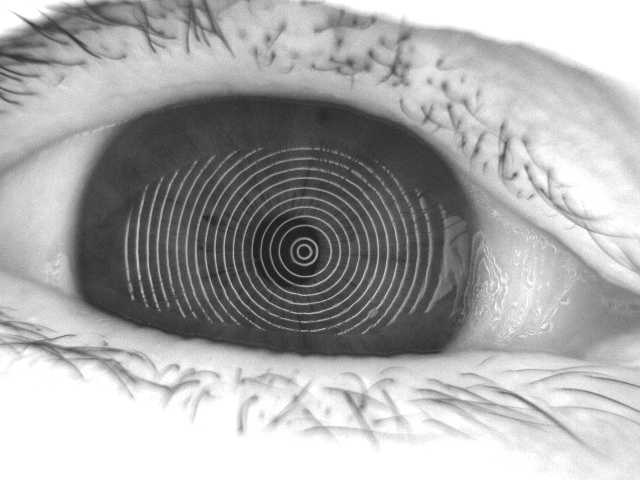

Supplement: Supplementary file 1 [file diagnostics-14-00052-s001.zip › Decreased wettability DE (DWDE)/Line break with rapid expansion/0023.jpeg]

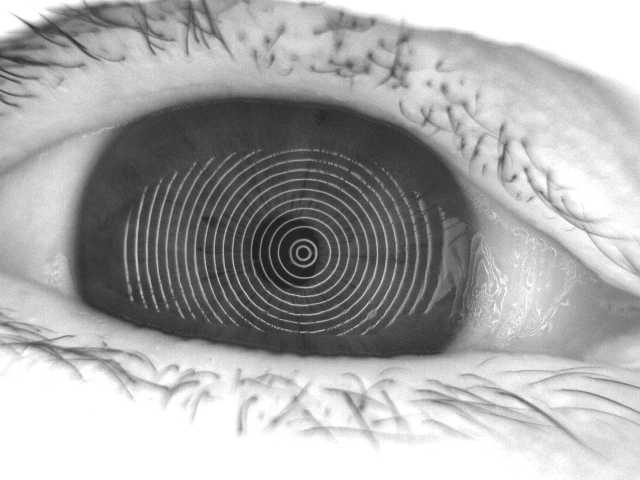

Supplement: Supplementary file 1 [file diagnostics-14-00052-s001.zip › Decreased wettability DE (DWDE)/Line break with rapid expansion/0024.jpeg]

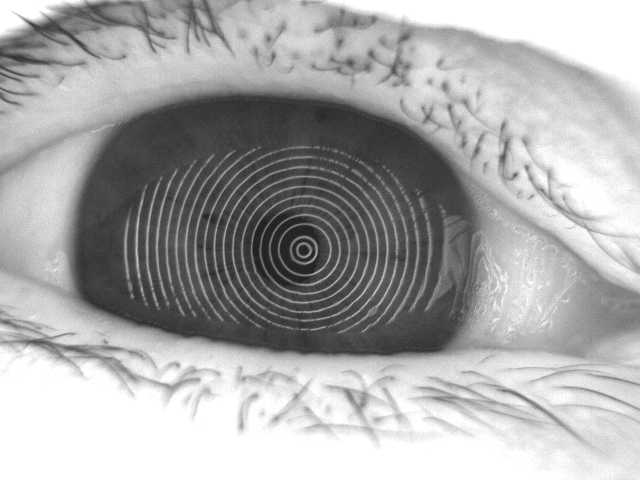

Supplement: Supplementary file 1 [file diagnostics-14-00052-s001.zip › Decreased wettability DE (DWDE)/Line break with rapid expansion/0025.jpeg]

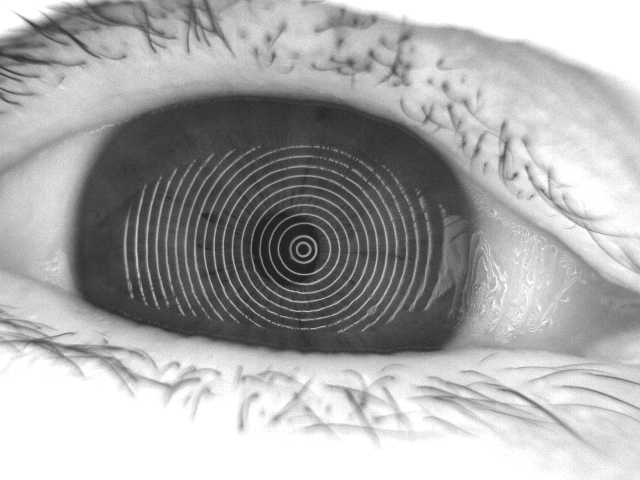

Supplement: Supplementary file 1 [file diagnostics-14-00052-s001.zip › Decreased wettability DE (DWDE)/Line break with rapid expansion/0026.jpeg]

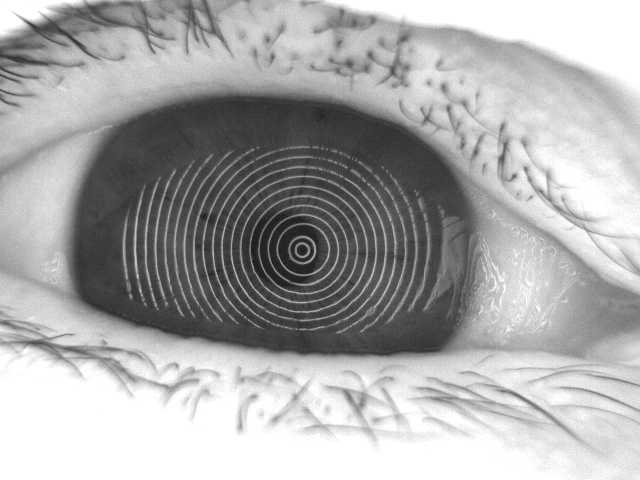

Supplement: Supplementary file 1 [file diagnostics-14-00052-s001.zip › Decreased wettability DE (DWDE)/Line break with rapid expansion/0027.jpeg]

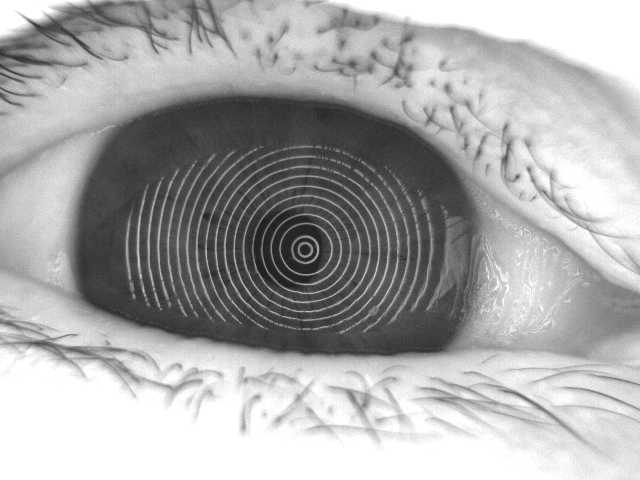

Supplement: Supplementary file 1 [file diagnostics-14-00052-s001.zip › Decreased wettability DE (DWDE)/Line break with rapid expansion/0028.jpeg]

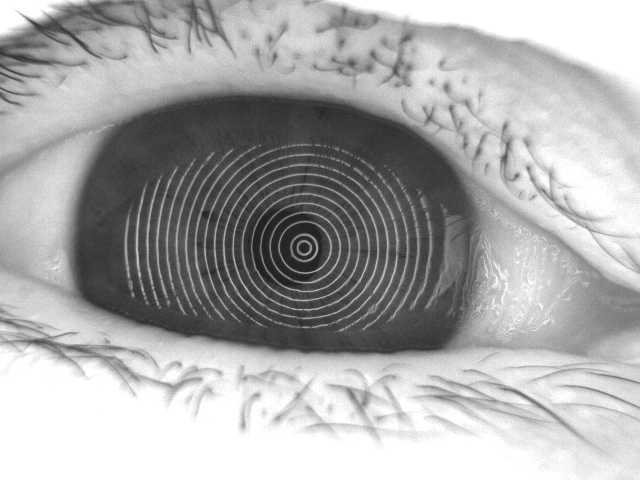

Supplement: Supplementary file 1 [file diagnostics-14-00052-s001.zip › Decreased wettability DE (DWDE)/Line break with rapid expansion/0029.jpeg]

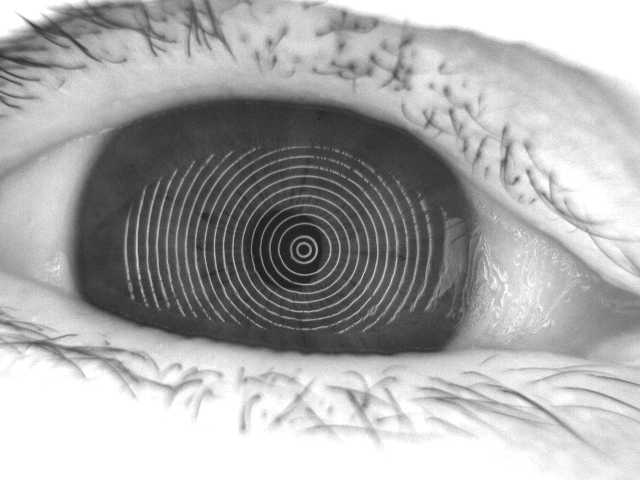

Supplement: Supplementary file 1 [file diagnostics-14-00052-s001.zip › Decreased wettability DE (DWDE)/Line break with rapid expansion/0030.jpeg]

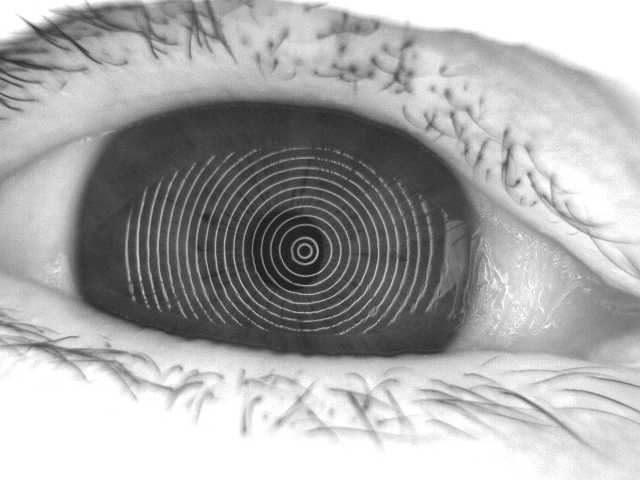

Supplement: Supplementary file 1 [file diagnostics-14-00052-s001.zip › Decreased wettability DE (DWDE)/Line break with rapid expansion/0031.jpeg]

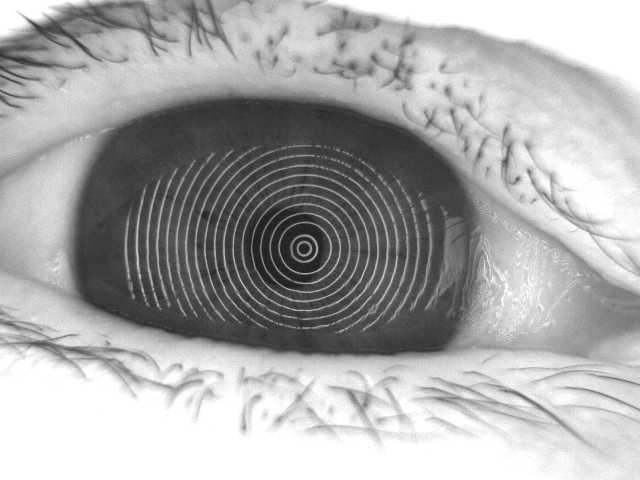

Supplement: Supplementary file 1 [file diagnostics-14-00052-s001.zip › Decreased wettability DE (DWDE)/Line break with rapid expansion/0032.jpeg]

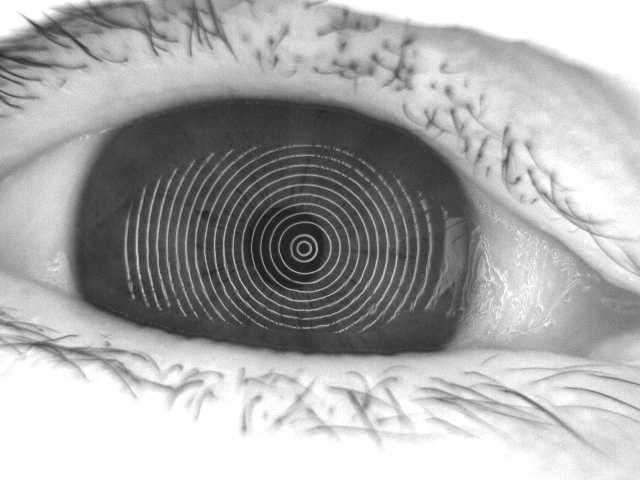

Supplement: Supplementary file 1 [file diagnostics-14-00052-s001.zip › Decreased wettability DE (DWDE)/Line break with rapid expansion/0033.jpeg]

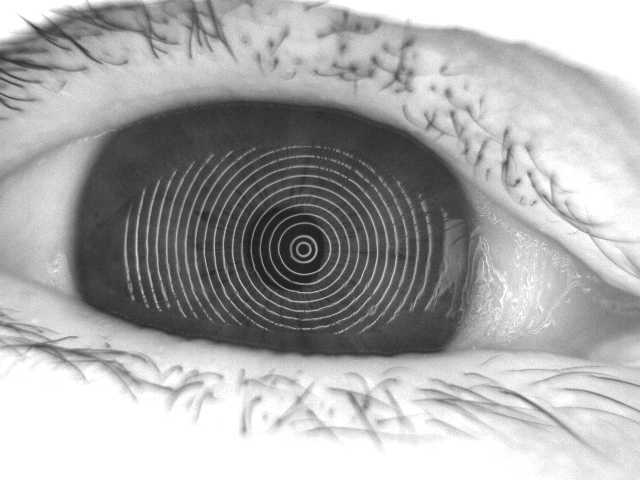

Supplement: Supplementary file 1 [file diagnostics-14-00052-s001.zip › Decreased wettability DE (DWDE)/Line break with rapid expansion/0034.jpeg]

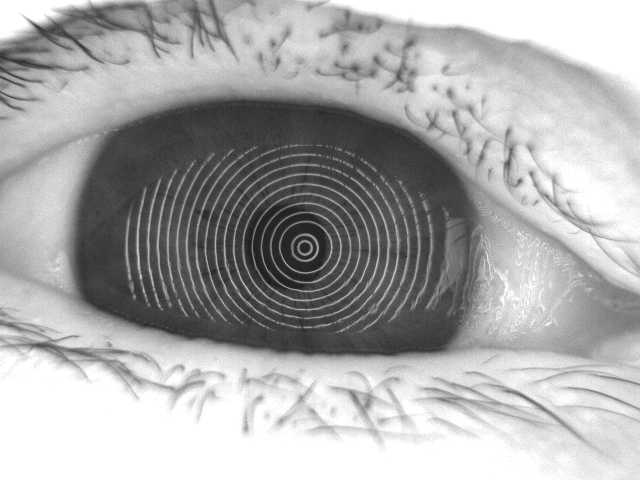

Supplement: Supplementary file 1 [file diagnostics-14-00052-s001.zip › Decreased wettability DE (DWDE)/Line break with rapid expansion/0035.jpeg]

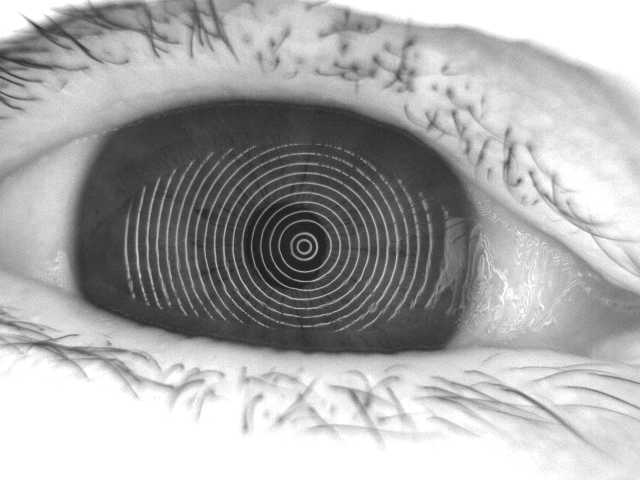

Supplement: Supplementary file 1 [file diagnostics-14-00052-s001.zip › Decreased wettability DE (DWDE)/Line break with rapid expansion/0036.jpeg]

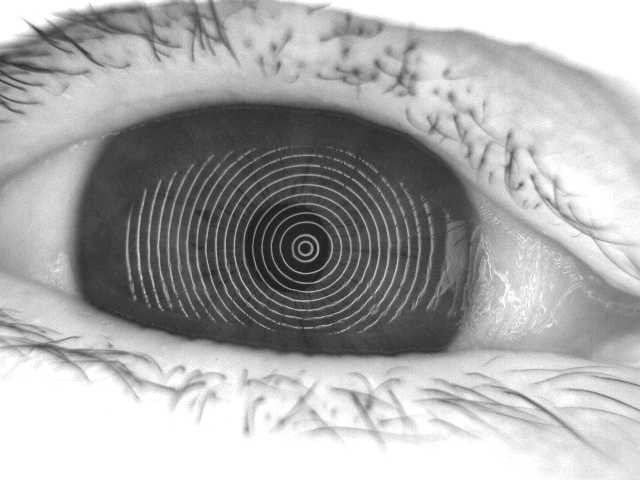

Supplement: Supplementary file 1 [file diagnostics-14-00052-s001.zip › Decreased wettability DE (DWDE)/Line break with rapid expansion/0037.jpeg]

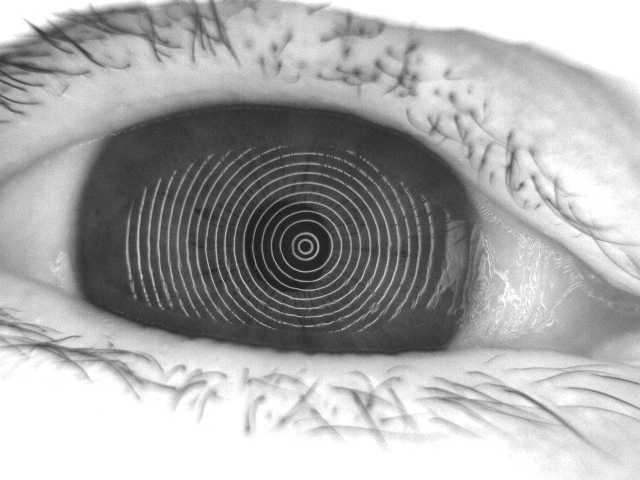

Supplement: Supplementary file 1 [file diagnostics-14-00052-s001.zip › Decreased wettability DE (DWDE)/Line break with rapid expansion/0038.jpeg]

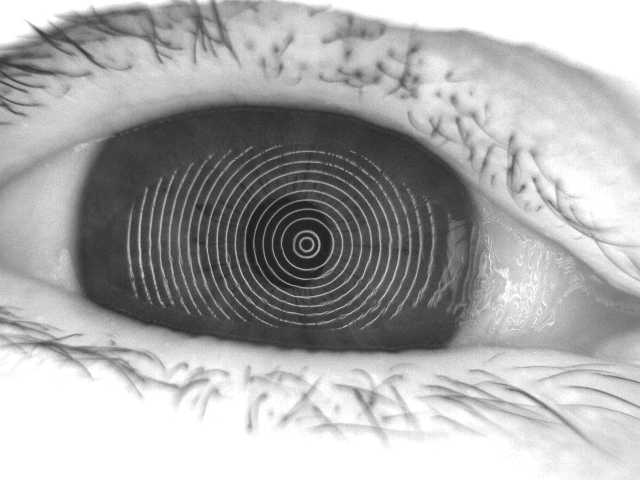

Supplement: Supplementary file 1 [file diagnostics-14-00052-s001.zip › Decreased wettability DE (DWDE)/Line break with rapid expansion/0039.jpeg]

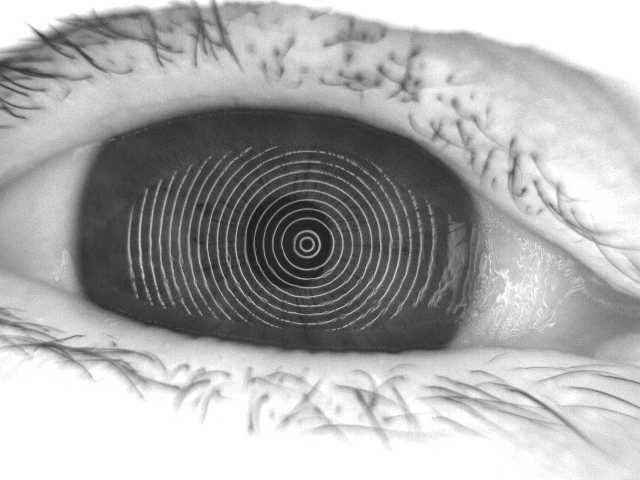

Supplement: Supplementary file 1 [file diagnostics-14-00052-s001.zip › Decreased wettability DE (DWDE)/Line break with rapid expansion/0040.jpeg]

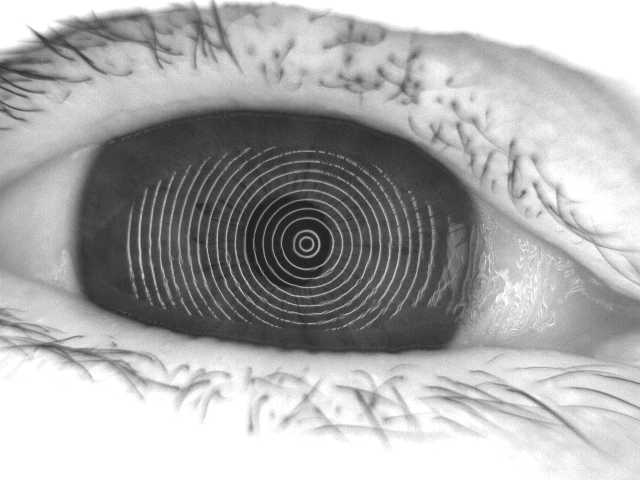

Supplement: Supplementary file 1 [file diagnostics-14-00052-s001.zip › Decreased wettability DE (DWDE)/Line break with rapid expansion/0041.jpeg]

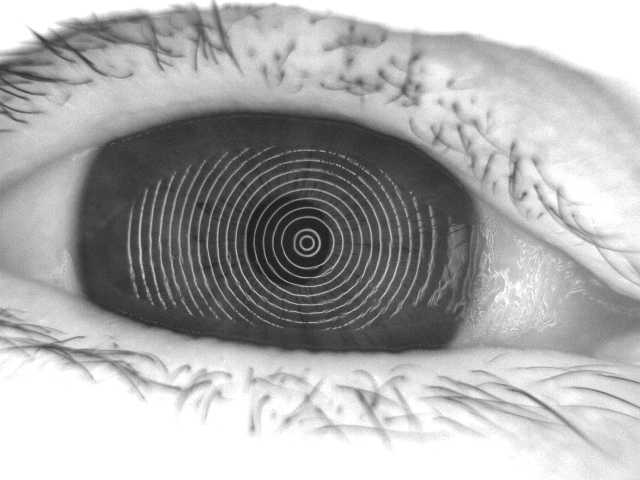

Supplement: Supplementary file 1 [file diagnostics-14-00052-s001.zip › Decreased wettability DE (DWDE)/Line break with rapid expansion/0042.jpeg]

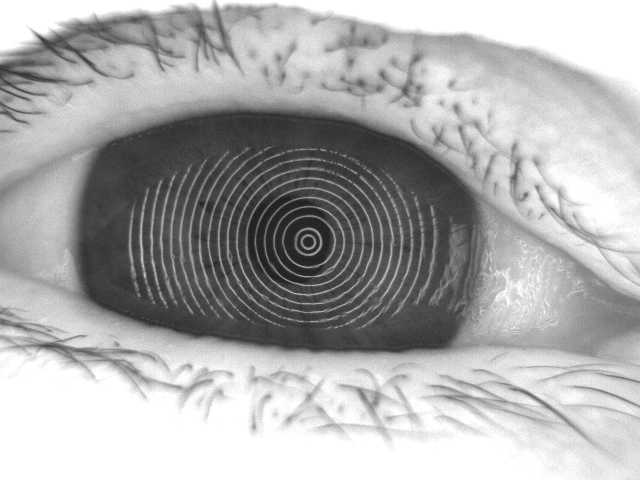

Supplement: Supplementary file 1 [file diagnostics-14-00052-s001.zip › Decreased wettability DE (DWDE)/Line break with rapid expansion/0043.jpeg]

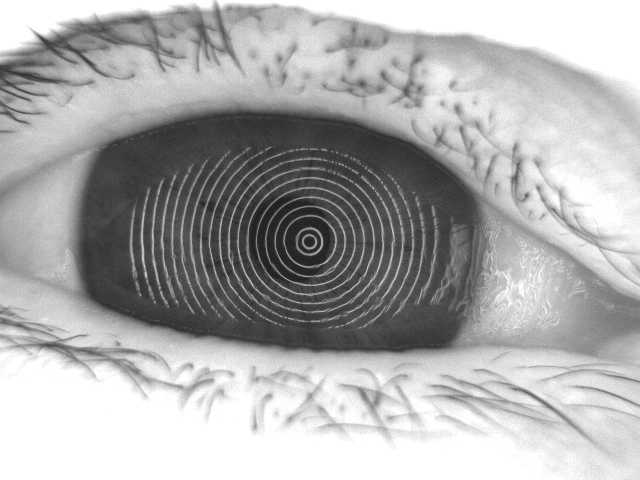

Supplement: Supplementary file 1 [file diagnostics-14-00052-s001.zip › Decreased wettability DE (DWDE)/Line break with rapid expansion/0044.jpeg]

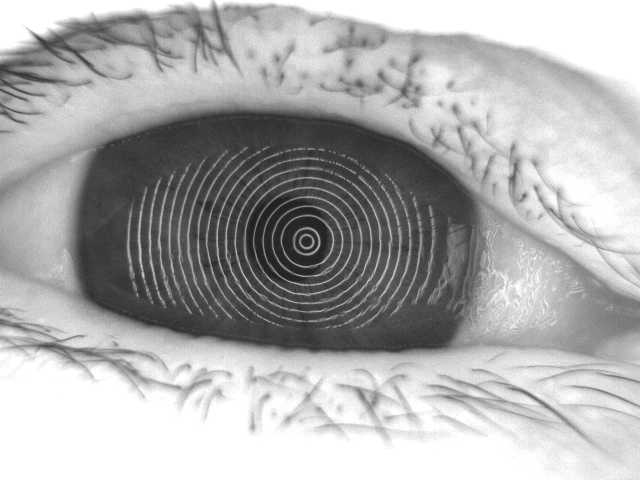

Supplement: Supplementary file 1 [file diagnostics-14-00052-s001.zip › Decreased wettability DE (DWDE)/Line break with rapid expansion/0045.jpeg]

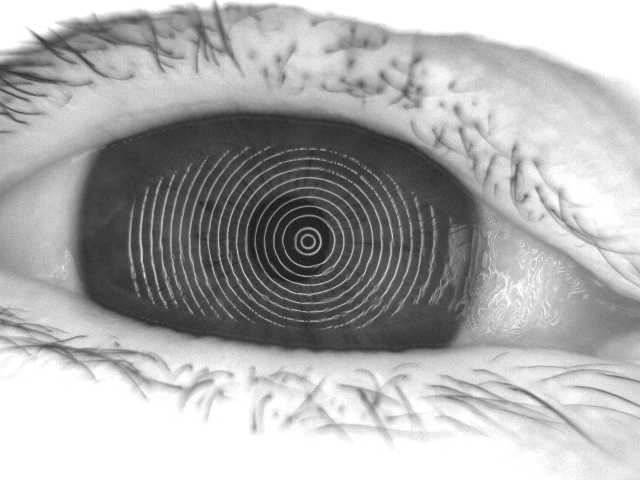

Supplement: Supplementary file 1 [file diagnostics-14-00052-s001.zip › Decreased wettability DE (DWDE)/Line break with rapid expansion/0046.jpeg]

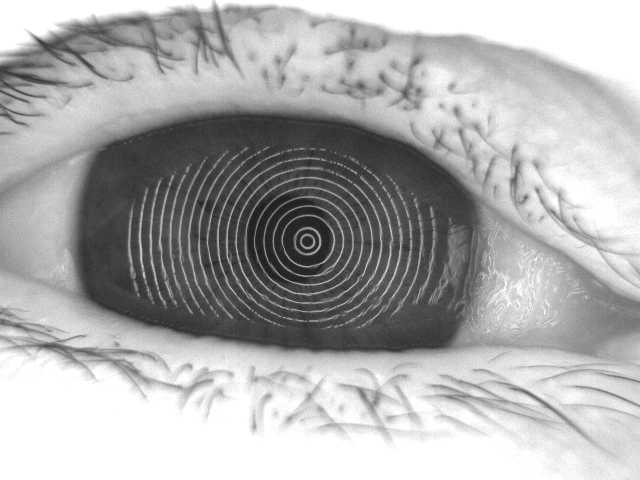

Supplement: Supplementary file 1 [file diagnostics-14-00052-s001.zip › Decreased wettability DE (DWDE)/Line break with rapid expansion/0047.jpeg]

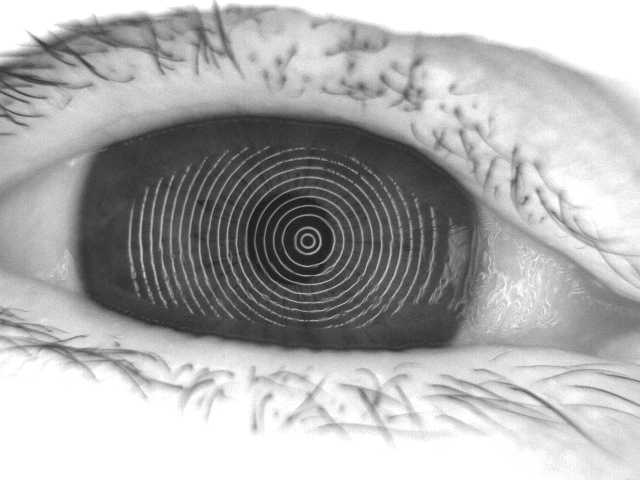

Supplement: Supplementary file 1 [file diagnostics-14-00052-s001.zip › Decreased wettability DE (DWDE)/Line break with rapid expansion/0048.jpeg]

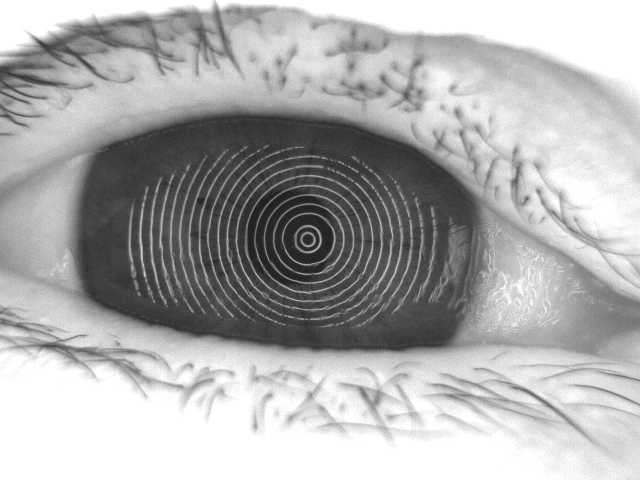

Supplement: Supplementary file 1 [file diagnostics-14-00052-s001.zip › Decreased wettability DE (DWDE)/Line break with rapid expansion/0049.jpeg]
