# Supplementary figures and images for: Dry Eye Subtype Classification Using Videokeratography and Deep Learning (part 1 of 2)
Source: Diagnostics (Basel). 2023 Dec 26;14(1):52. doi: 10.3390/diagnostics14010052 (PMC10802766; doi:10.3390/diagnostics14010052)

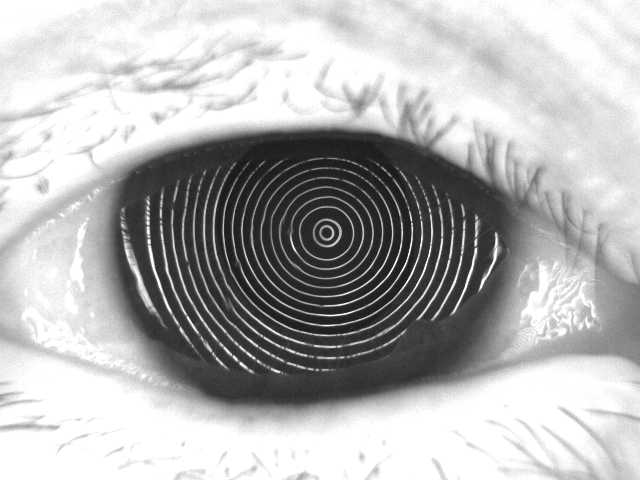

Supplement: Supplementary file 1 [file diagnostics-14-00052-s001.zip › Decreased wettability DE (DWDE)/Dimple break/0000.jpeg]

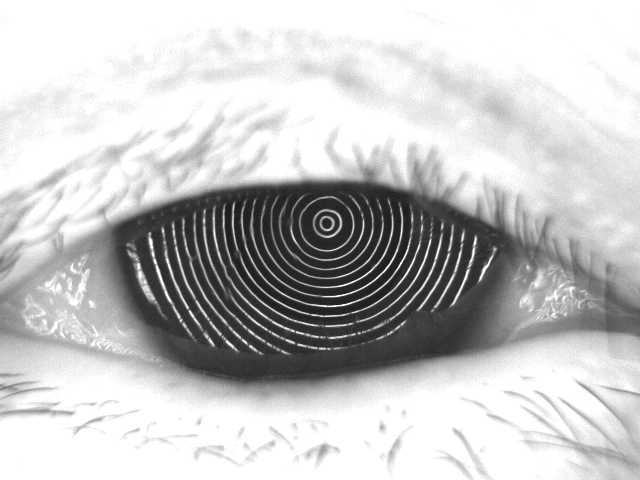

Supplement: Supplementary file 1 [file diagnostics-14-00052-s001.zip › Decreased wettability DE (DWDE)/Dimple break/0001.jpeg]

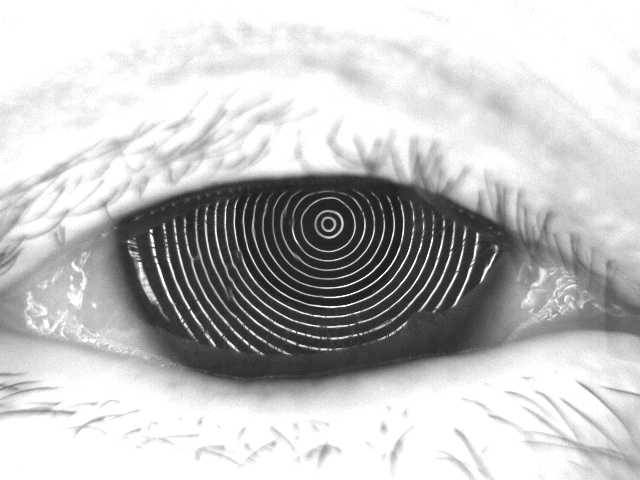

Supplement: Supplementary file 1 [file diagnostics-14-00052-s001.zip › Decreased wettability DE (DWDE)/Dimple break/0002.jpeg]

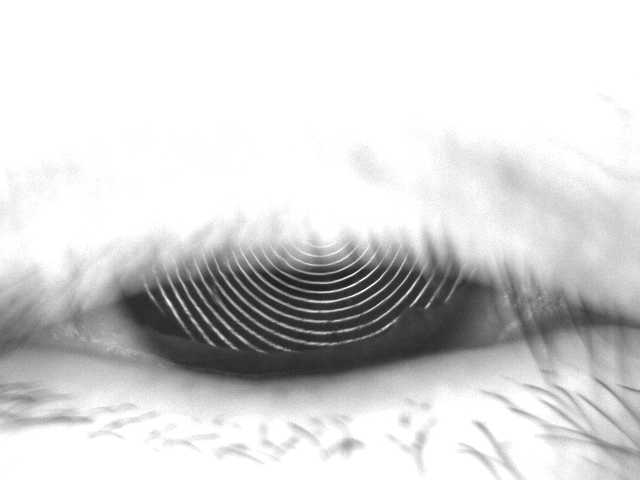

Supplement: Supplementary file 1 [file diagnostics-14-00052-s001.zip › Decreased wettability DE (DWDE)/Dimple break/0003.jpeg]

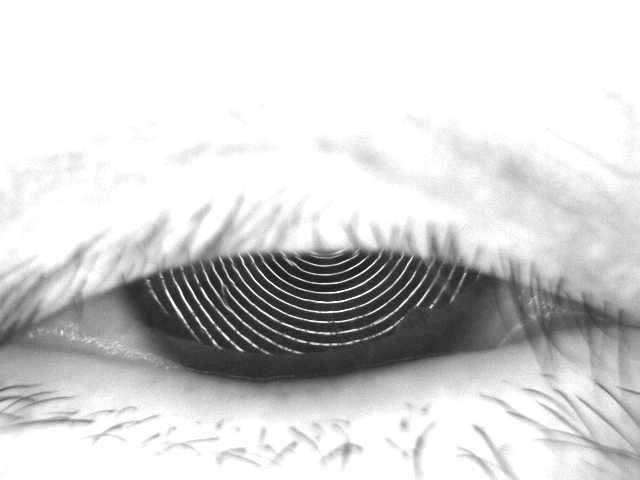

Supplement: Supplementary file 1 [file diagnostics-14-00052-s001.zip › Decreased wettability DE (DWDE)/Dimple break/0004.jpeg]

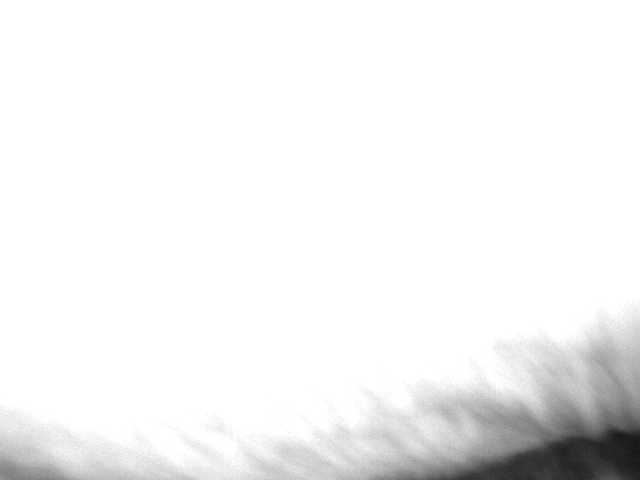

Supplement: Supplementary file 1 [file diagnostics-14-00052-s001.zip › Decreased wettability DE (DWDE)/Dimple break/0005.jpeg]

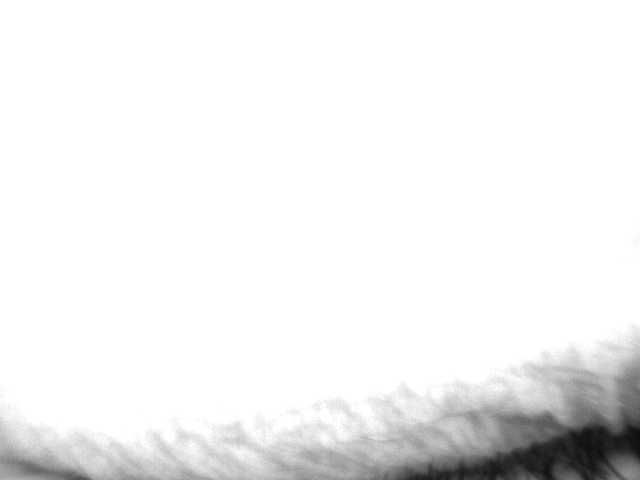

Supplement: Supplementary file 1 [file diagnostics-14-00052-s001.zip › Decreased wettability DE (DWDE)/Dimple break/0006.jpeg]

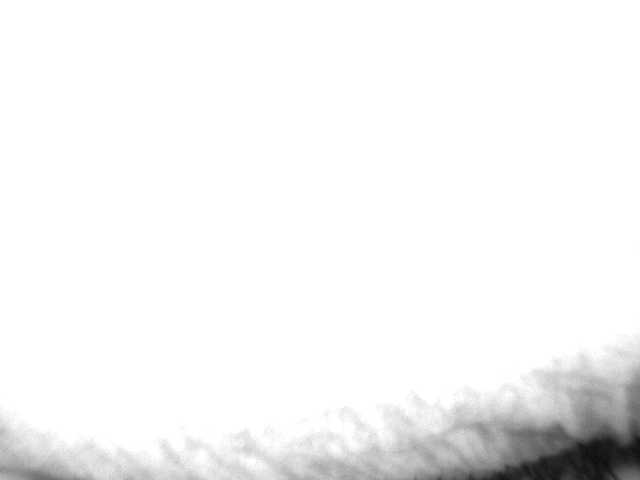

Supplement: Supplementary file 1 [file diagnostics-14-00052-s001.zip › Decreased wettability DE (DWDE)/Dimple break/0007.jpeg]

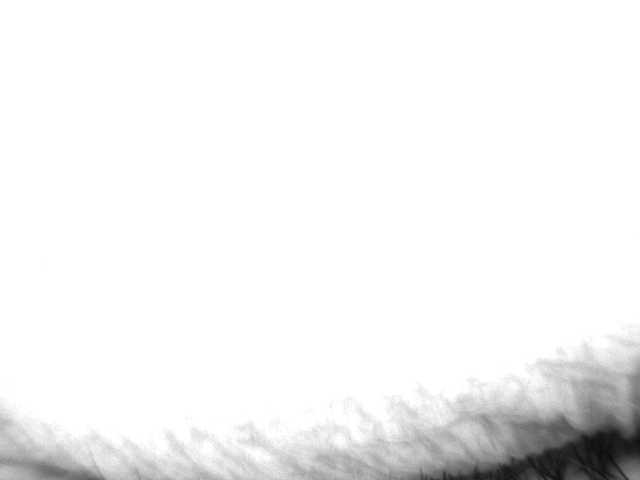

Supplement: Supplementary file 1 [file diagnostics-14-00052-s001.zip › Decreased wettability DE (DWDE)/Dimple break/0008.jpeg]

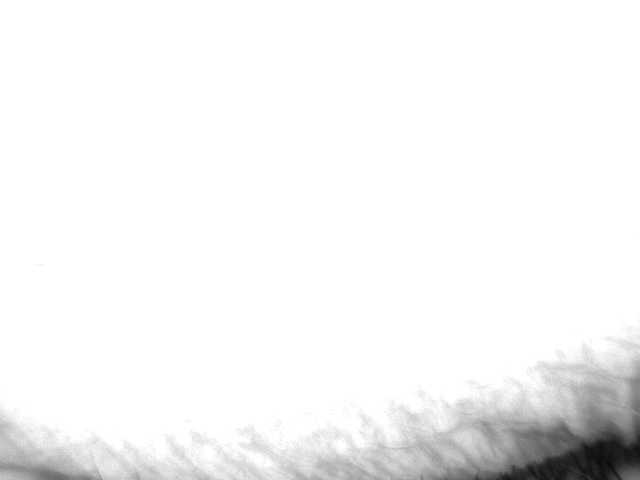

Supplement: Supplementary file 1 [file diagnostics-14-00052-s001.zip › Decreased wettability DE (DWDE)/Dimple break/0009.jpeg]

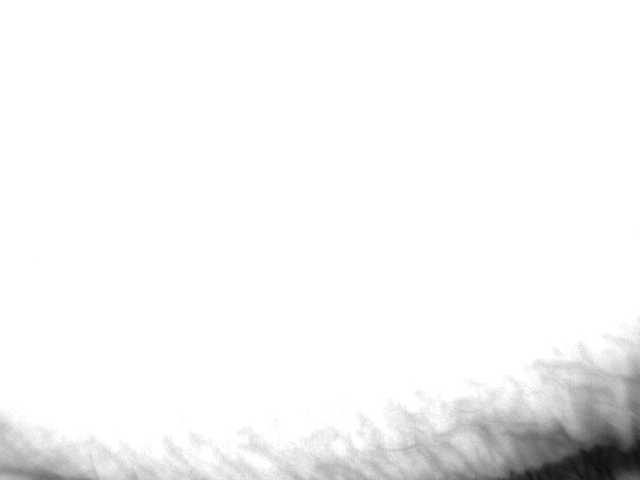

Supplement: Supplementary file 1 [file diagnostics-14-00052-s001.zip › Decreased wettability DE (DWDE)/Dimple break/0010.jpeg]

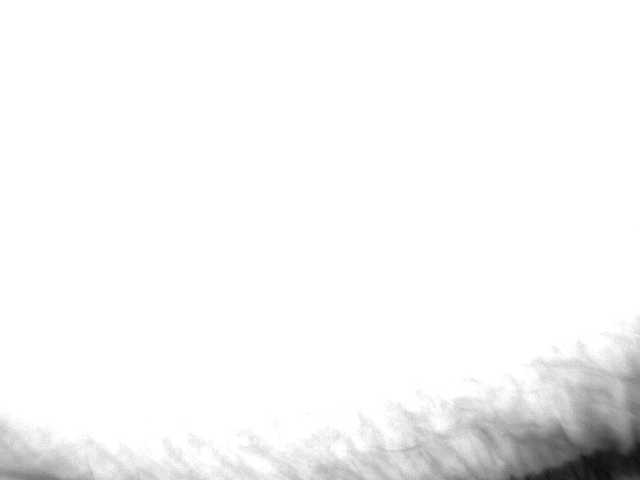

Supplement: Supplementary file 1 [file diagnostics-14-00052-s001.zip › Decreased wettability DE (DWDE)/Dimple break/0011.jpeg]

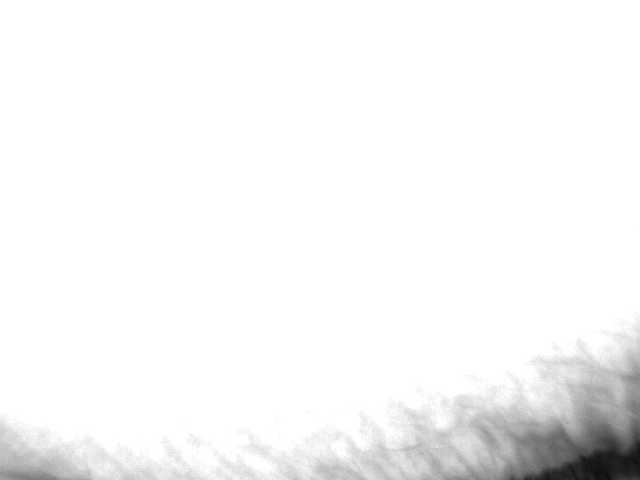

Supplement: Supplementary file 1 [file diagnostics-14-00052-s001.zip › Decreased wettability DE (DWDE)/Dimple break/0012.jpeg]

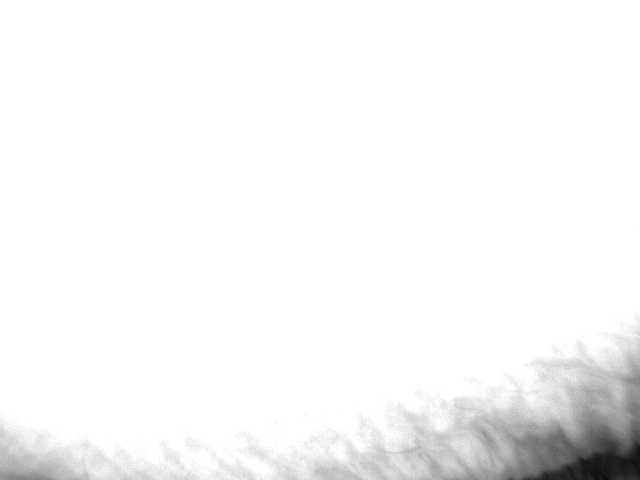

Supplement: Supplementary file 1 [file diagnostics-14-00052-s001.zip › Decreased wettability DE (DWDE)/Dimple break/0013.jpeg]

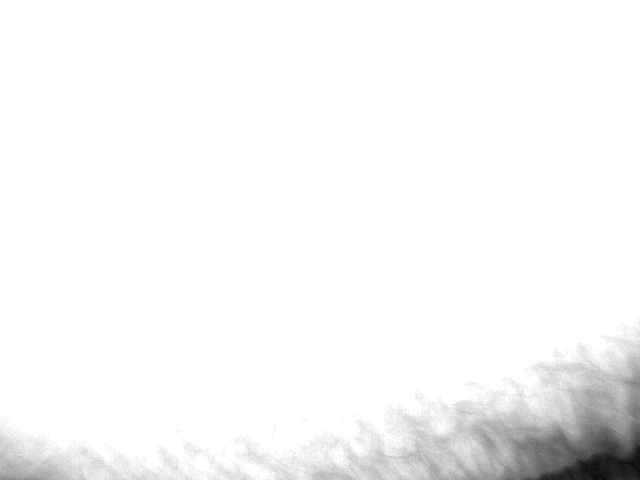

Supplement: Supplementary file 1 [file diagnostics-14-00052-s001.zip › Decreased wettability DE (DWDE)/Dimple break/0014.jpeg]

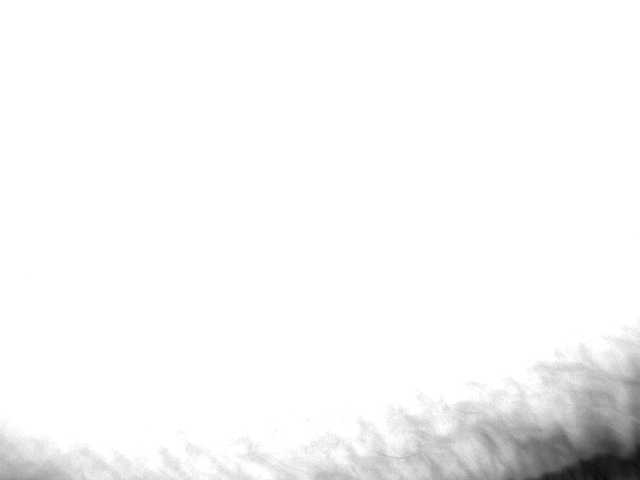

Supplement: Supplementary file 1 [file diagnostics-14-00052-s001.zip › Decreased wettability DE (DWDE)/Dimple break/0015.jpeg]

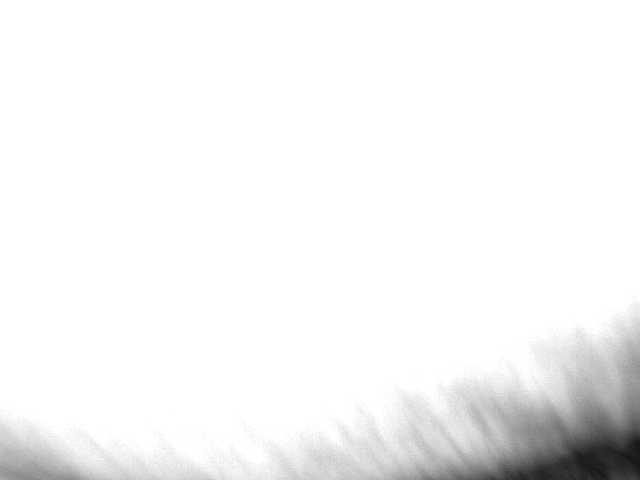

Supplement: Supplementary file 1 [file diagnostics-14-00052-s001.zip › Decreased wettability DE (DWDE)/Dimple break/0016.jpeg]

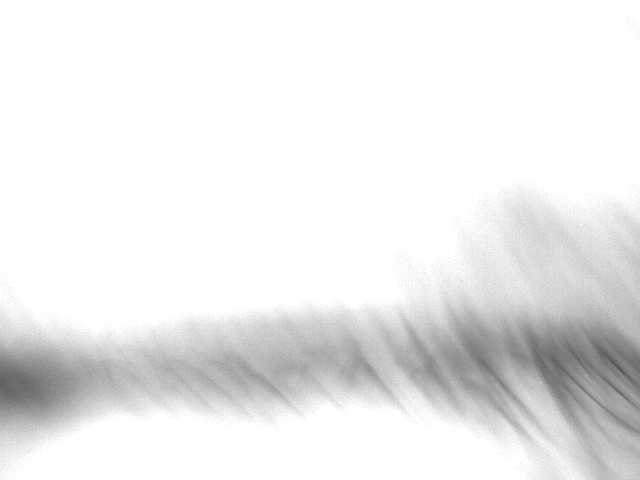

Supplement: Supplementary file 1 [file diagnostics-14-00052-s001.zip › Decreased wettability DE (DWDE)/Dimple break/0017.jpeg]

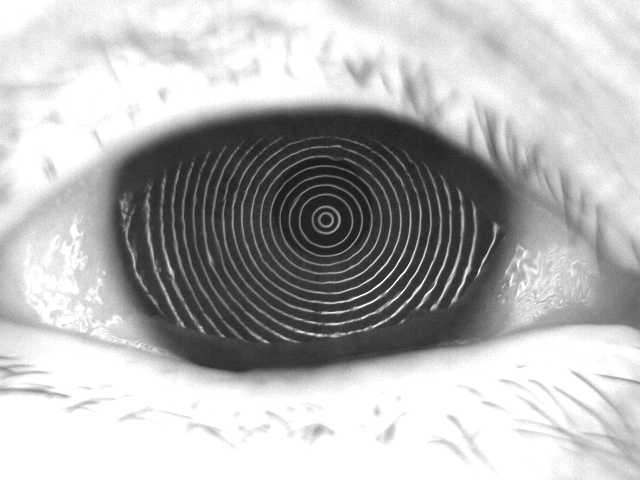

Supplement: Supplementary file 1 [file diagnostics-14-00052-s001.zip › Decreased wettability DE (DWDE)/Dimple break/0018.jpeg]

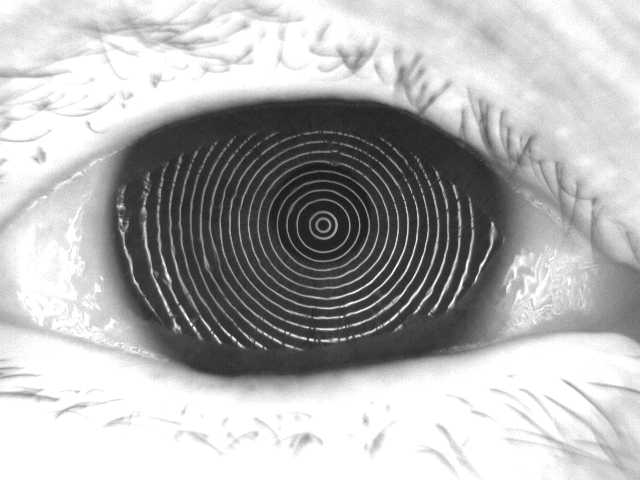

Supplement: Supplementary file 1 [file diagnostics-14-00052-s001.zip › Decreased wettability DE (DWDE)/Dimple break/0019.jpeg]

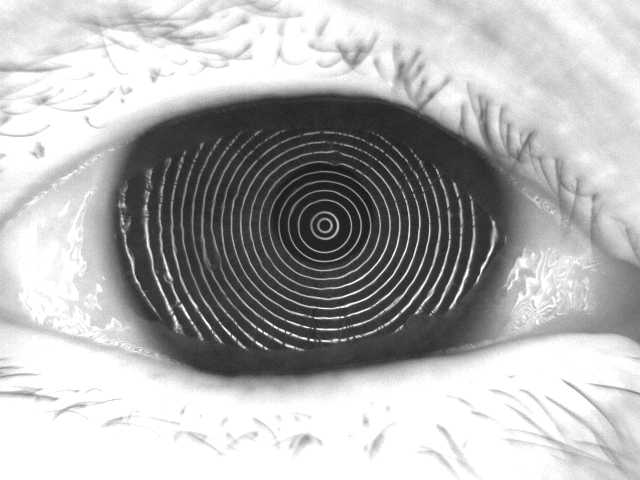

Supplement: Supplementary file 1 [file diagnostics-14-00052-s001.zip › Decreased wettability DE (DWDE)/Dimple break/0020.jpeg]

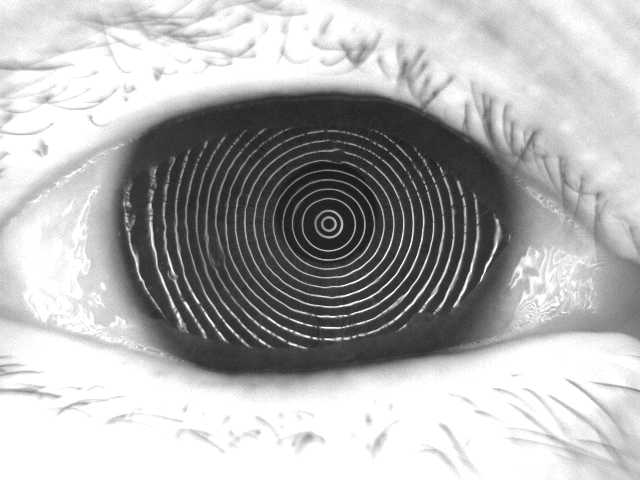

Supplement: Supplementary file 1 [file diagnostics-14-00052-s001.zip › Decreased wettability DE (DWDE)/Dimple break/0021.jpeg]

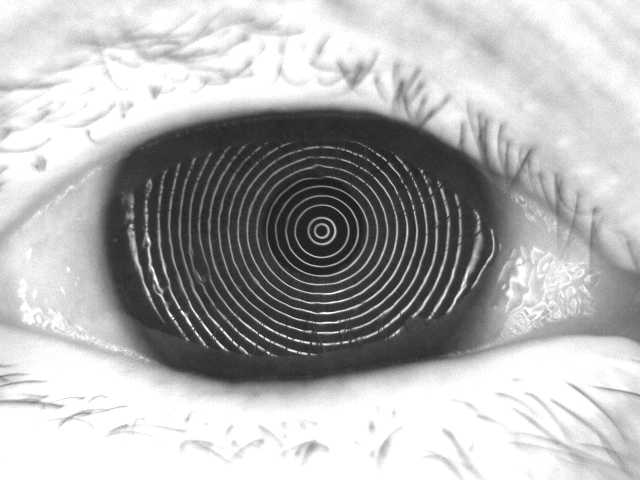

Supplement: Supplementary file 1 [file diagnostics-14-00052-s001.zip › Decreased wettability DE (DWDE)/Dimple break/0022.jpeg]

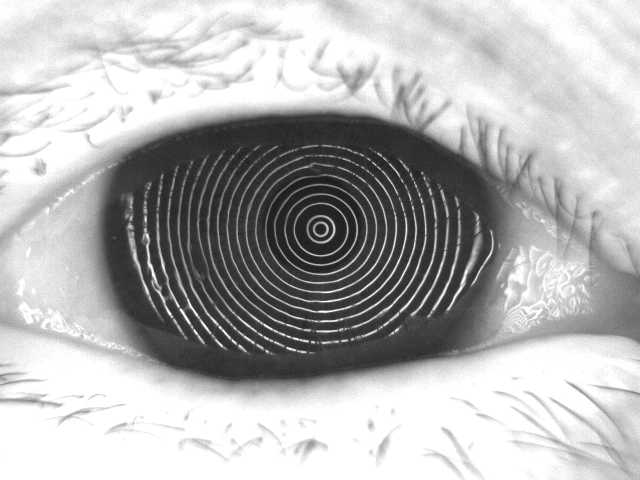

Supplement: Supplementary file 1 [file diagnostics-14-00052-s001.zip › Decreased wettability DE (DWDE)/Dimple break/0023.jpeg]

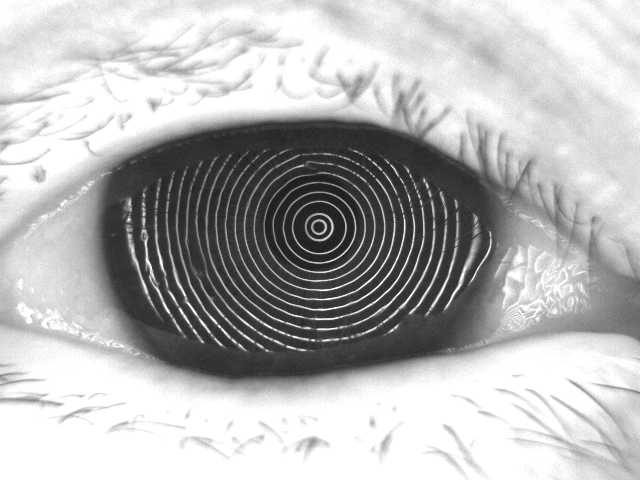

Supplement: Supplementary file 1 [file diagnostics-14-00052-s001.zip › Decreased wettability DE (DWDE)/Dimple break/0024.jpeg]

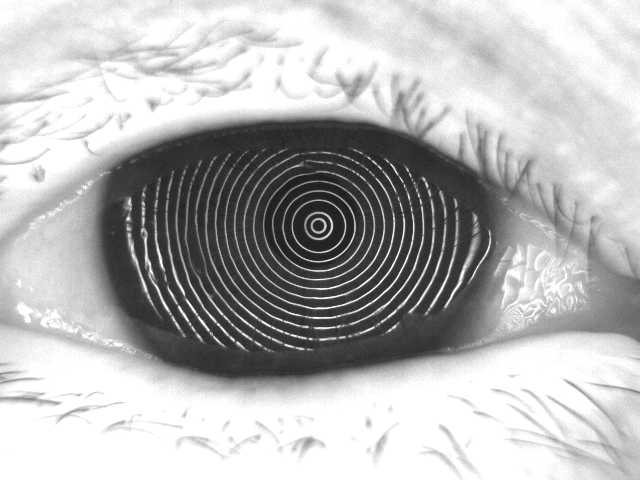

Supplement: Supplementary file 1 [file diagnostics-14-00052-s001.zip › Decreased wettability DE (DWDE)/Dimple break/0025.jpeg]

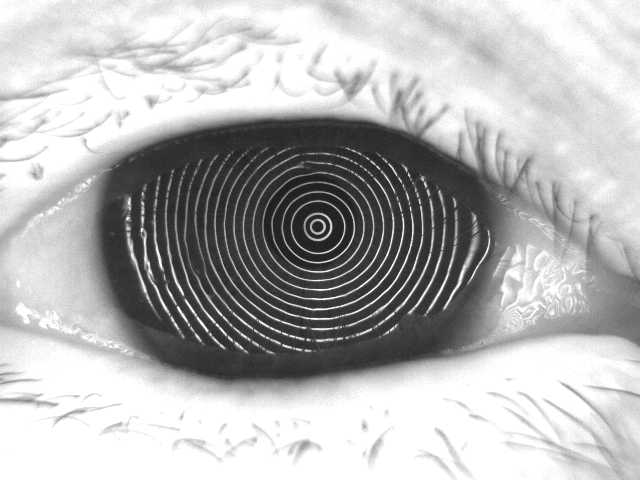

Supplement: Supplementary file 1 [file diagnostics-14-00052-s001.zip › Decreased wettability DE (DWDE)/Dimple break/0026.jpeg]

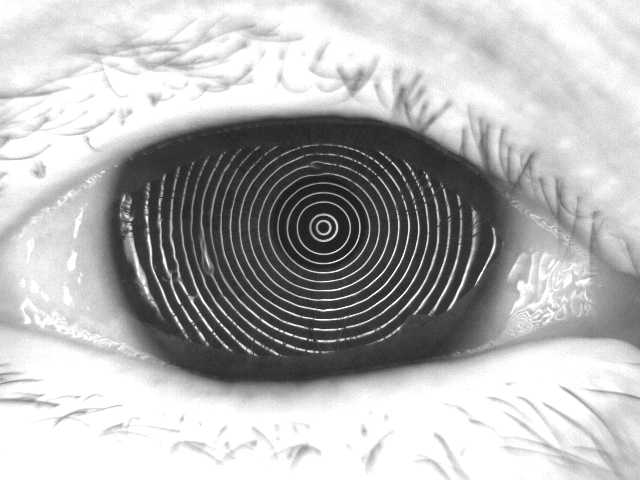

Supplement: Supplementary file 1 [file diagnostics-14-00052-s001.zip › Decreased wettability DE (DWDE)/Dimple break/0027.jpeg]

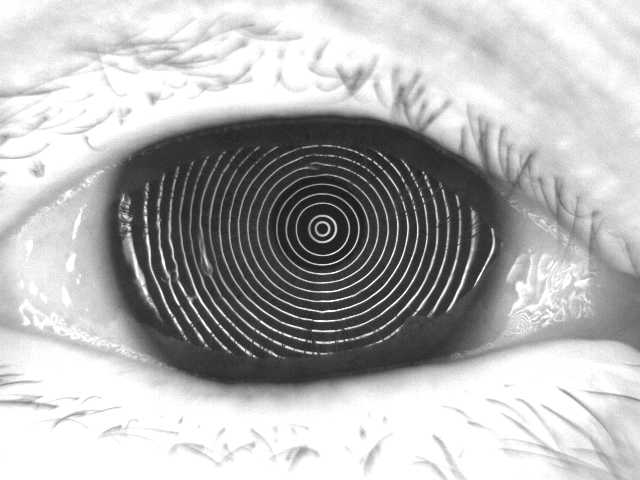

Supplement: Supplementary file 1 [file diagnostics-14-00052-s001.zip › Decreased wettability DE (DWDE)/Dimple break/0028.jpeg]

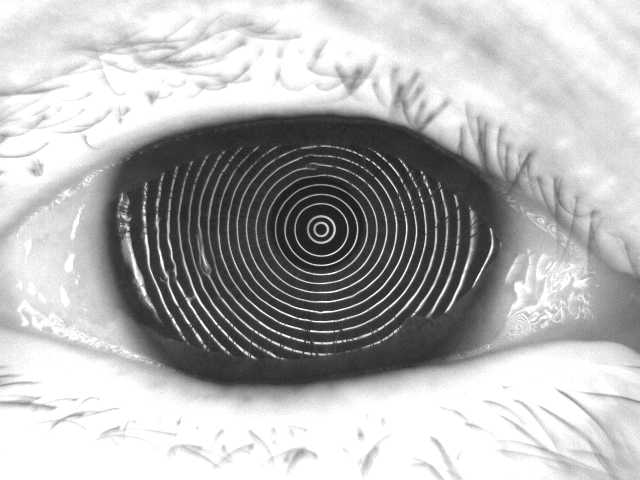

Supplement: Supplementary file 1 [file diagnostics-14-00052-s001.zip › Decreased wettability DE (DWDE)/Dimple break/0029.jpeg]

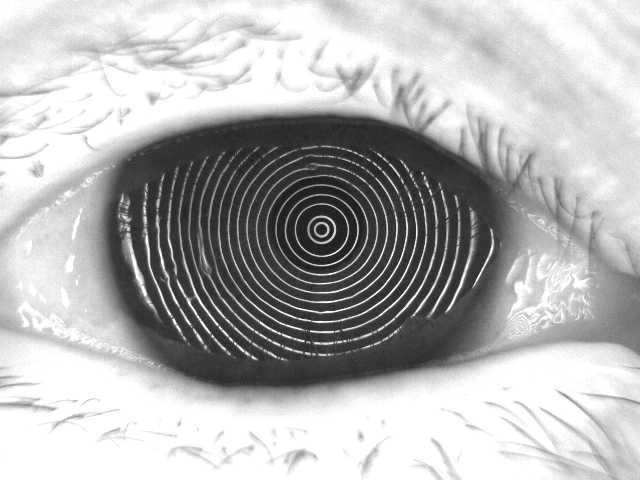

Supplement: Supplementary file 1 [file diagnostics-14-00052-s001.zip › Decreased wettability DE (DWDE)/Dimple break/0030.jpeg]

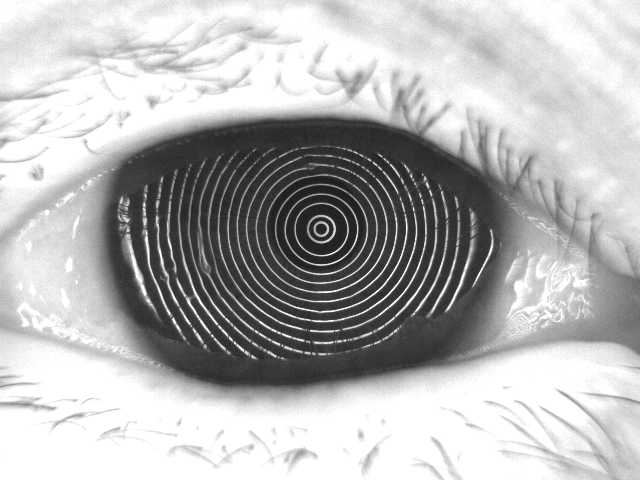

Supplement: Supplementary file 1 [file diagnostics-14-00052-s001.zip › Decreased wettability DE (DWDE)/Dimple break/0031.jpeg]

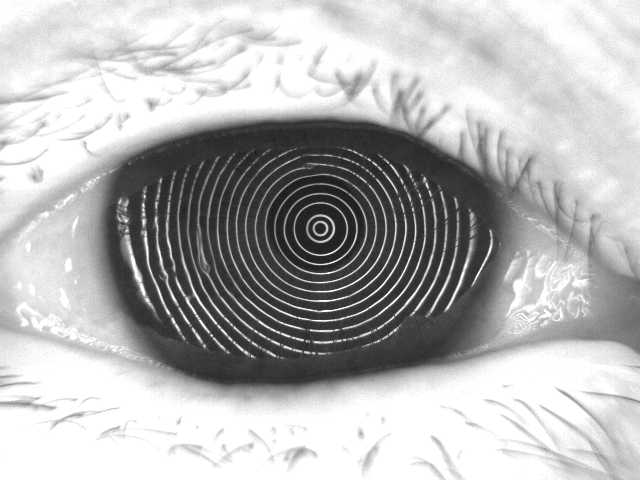

Supplement: Supplementary file 1 [file diagnostics-14-00052-s001.zip › Decreased wettability DE (DWDE)/Dimple break/0032.jpeg]

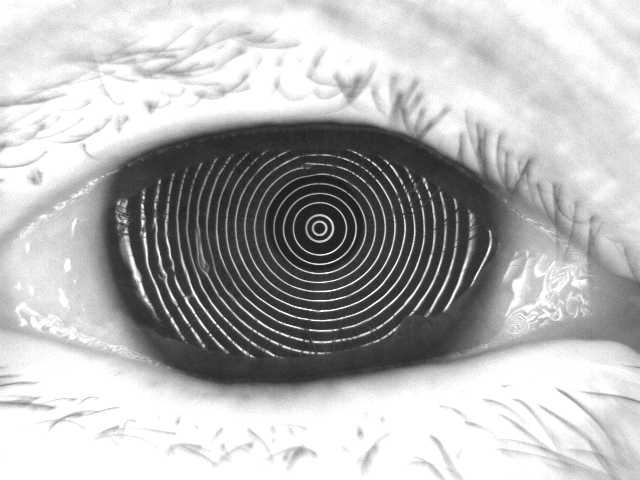

Supplement: Supplementary file 1 [file diagnostics-14-00052-s001.zip › Decreased wettability DE (DWDE)/Dimple break/0033.jpeg]

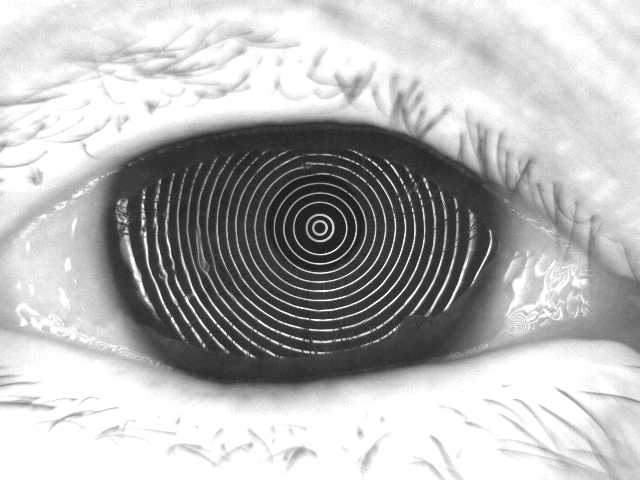

Supplement: Supplementary file 1 [file diagnostics-14-00052-s001.zip › Decreased wettability DE (DWDE)/Dimple break/0034.jpeg]

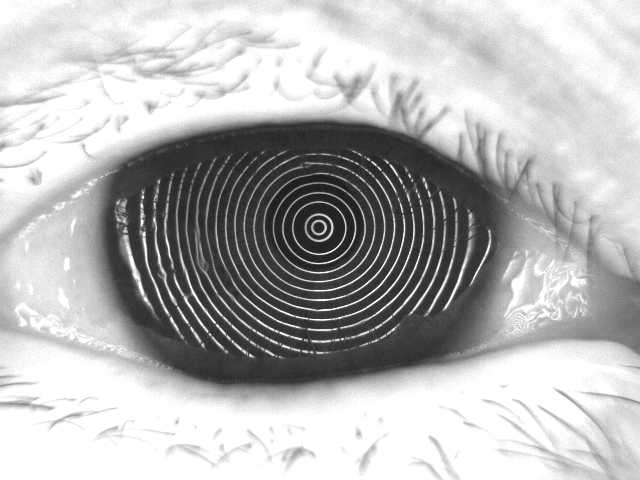

Supplement: Supplementary file 1 [file diagnostics-14-00052-s001.zip › Decreased wettability DE (DWDE)/Dimple break/0035.jpeg]

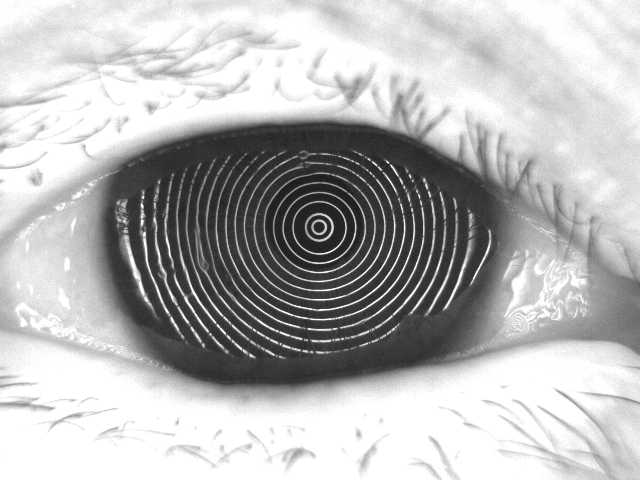

Supplement: Supplementary file 1 [file diagnostics-14-00052-s001.zip › Decreased wettability DE (DWDE)/Dimple break/0036.jpeg]

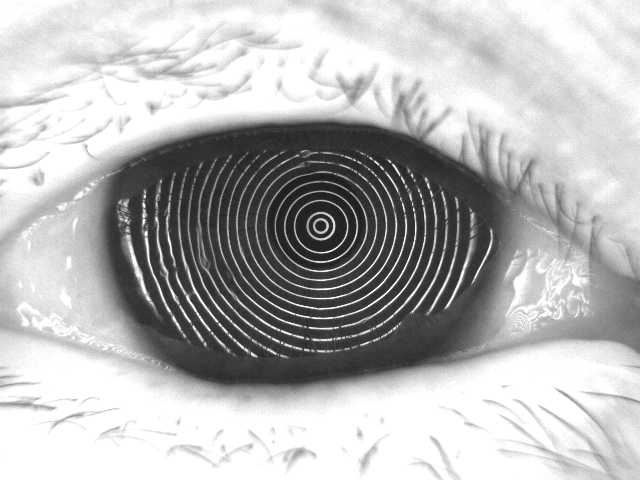

Supplement: Supplementary file 1 [file diagnostics-14-00052-s001.zip › Decreased wettability DE (DWDE)/Dimple break/0037.jpeg]

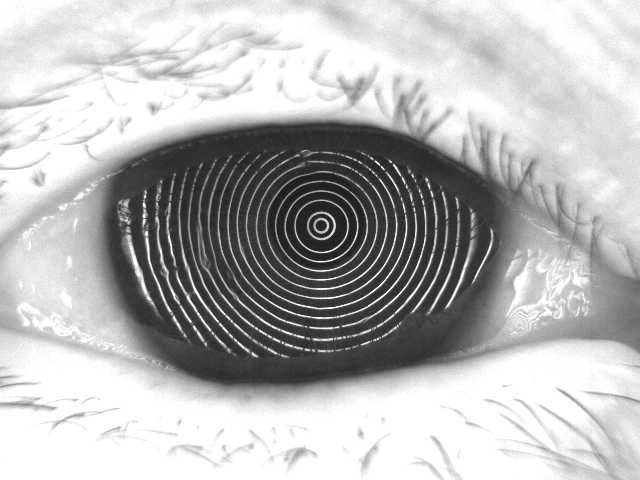

Supplement: Supplementary file 1 [file diagnostics-14-00052-s001.zip › Decreased wettability DE (DWDE)/Dimple break/0038.jpeg]

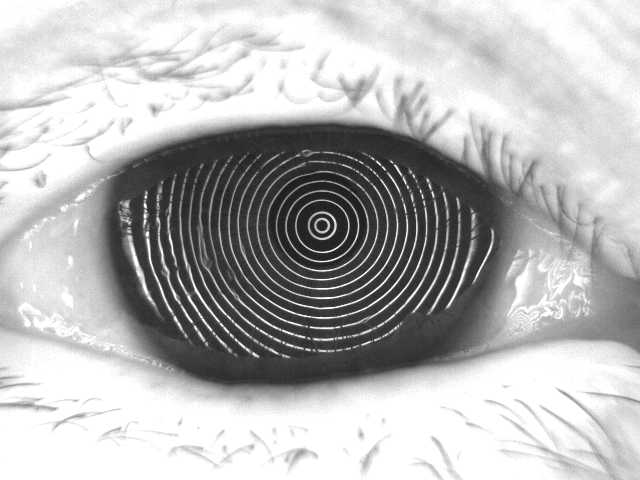

Supplement: Supplementary file 1 [file diagnostics-14-00052-s001.zip › Decreased wettability DE (DWDE)/Dimple break/0039.jpeg]

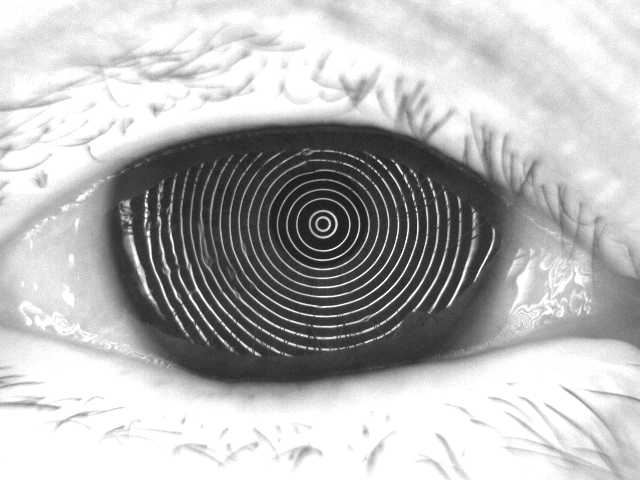

Supplement: Supplementary file 1 [file diagnostics-14-00052-s001.zip › Decreased wettability DE (DWDE)/Dimple break/0040.jpeg]

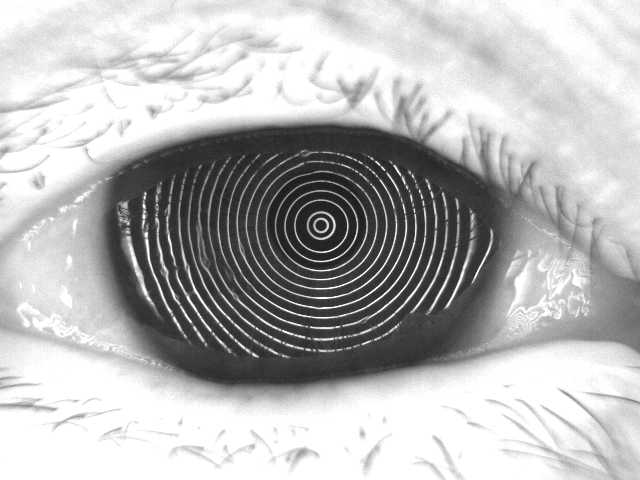

Supplement: Supplementary file 1 [file diagnostics-14-00052-s001.zip › Decreased wettability DE (DWDE)/Dimple break/0041.jpeg]

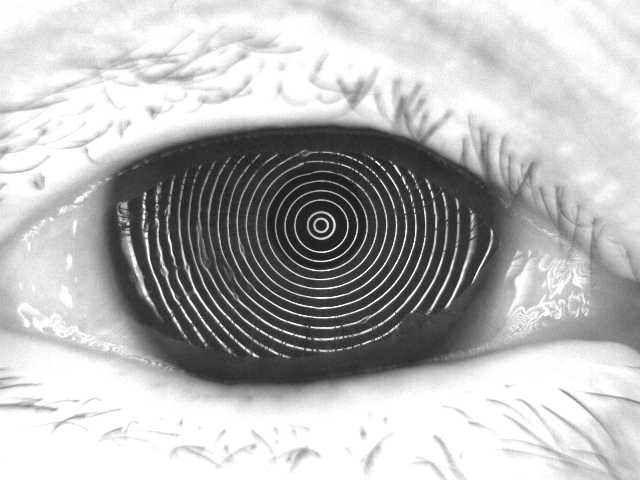

Supplement: Supplementary file 1 [file diagnostics-14-00052-s001.zip › Decreased wettability DE (DWDE)/Dimple break/0042.jpeg]

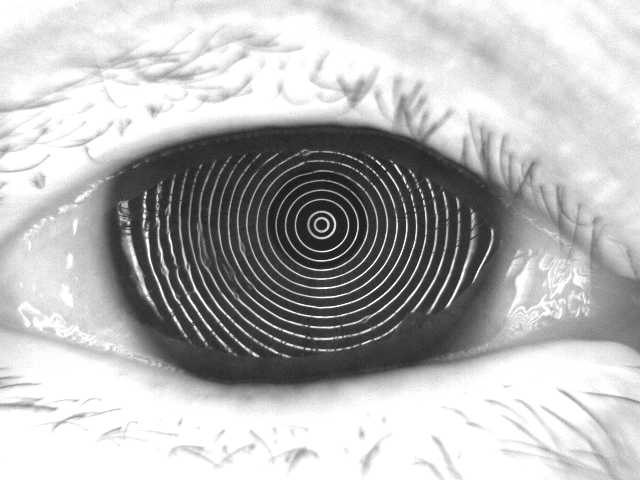

Supplement: Supplementary file 1 [file diagnostics-14-00052-s001.zip › Decreased wettability DE (DWDE)/Dimple break/0043.jpeg]

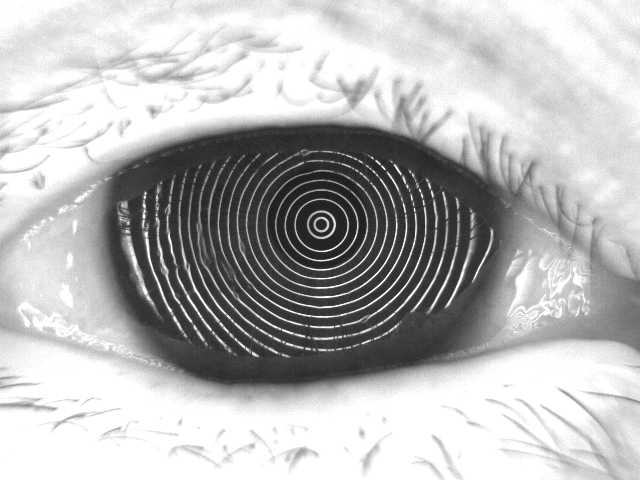

Supplement: Supplementary file 1 [file diagnostics-14-00052-s001.zip › Decreased wettability DE (DWDE)/Dimple break/0044.jpeg]

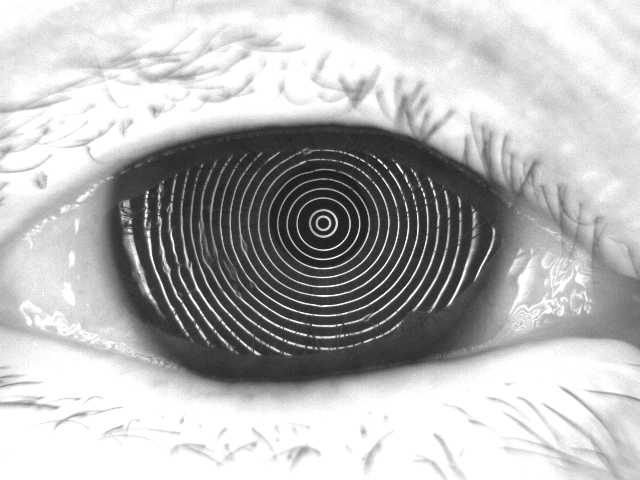

Supplement: Supplementary file 1 [file diagnostics-14-00052-s001.zip › Decreased wettability DE (DWDE)/Dimple break/0045.jpeg]

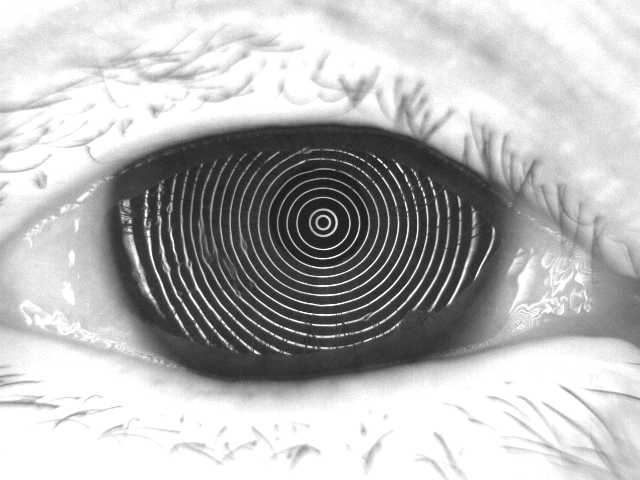

Supplement: Supplementary file 1 [file diagnostics-14-00052-s001.zip › Decreased wettability DE (DWDE)/Dimple break/0046.jpeg]

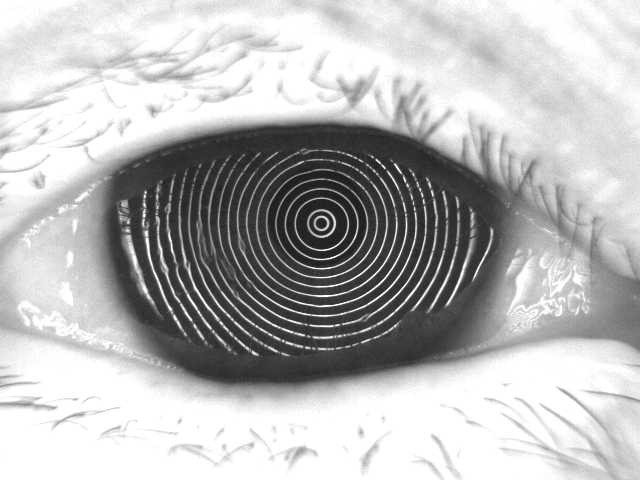

Supplement: Supplementary file 1 [file diagnostics-14-00052-s001.zip › Decreased wettability DE (DWDE)/Dimple break/0047.jpeg]

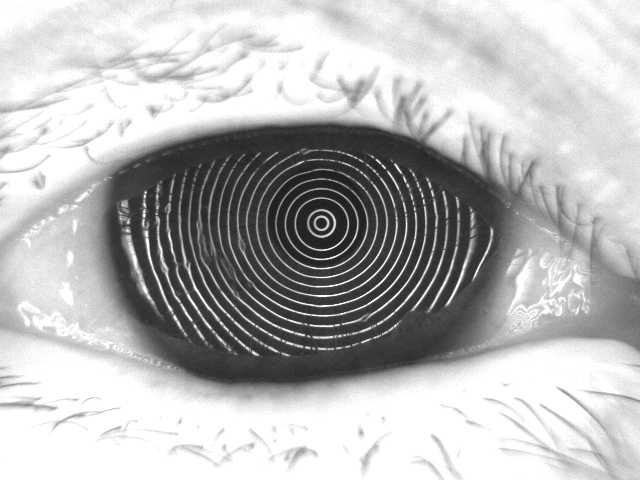

Supplement: Supplementary file 1 [file diagnostics-14-00052-s001.zip › Decreased wettability DE (DWDE)/Dimple break/0048.jpeg]

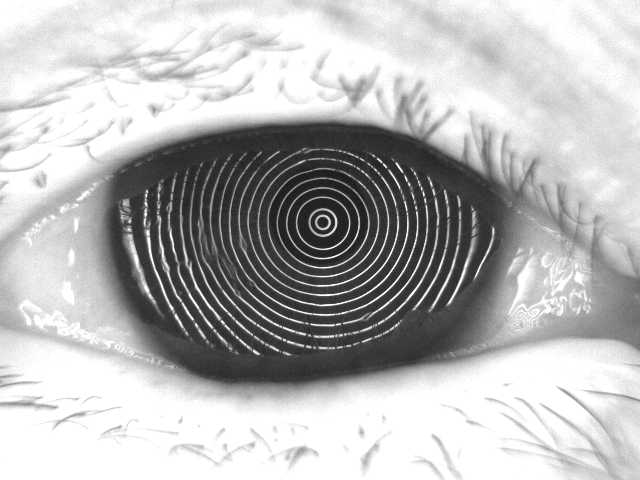

Supplement: Supplementary file 1 [file diagnostics-14-00052-s001.zip › Decreased wettability DE (DWDE)/Dimple break/0049.jpeg]

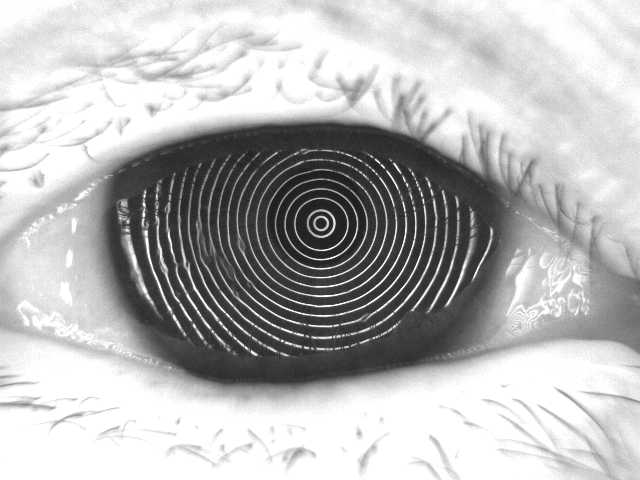

Supplement: Supplementary file 1 [file diagnostics-14-00052-s001.zip › Decreased wettability DE (DWDE)/Dimple break/0050.jpeg]

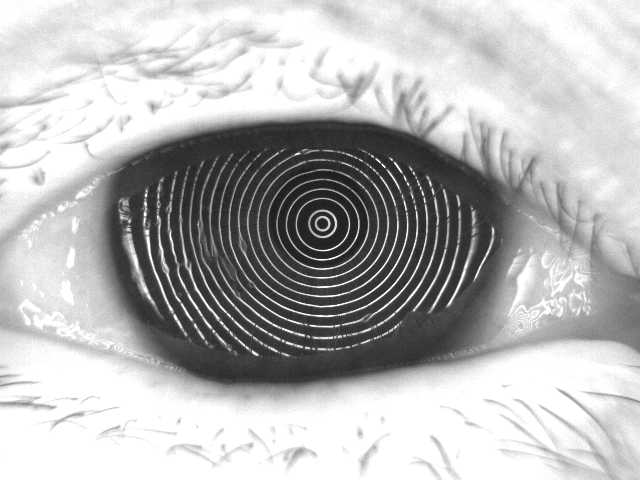

Supplement: Supplementary file 1 [file diagnostics-14-00052-s001.zip › Decreased wettability DE (DWDE)/Dimple break/0051.jpeg]

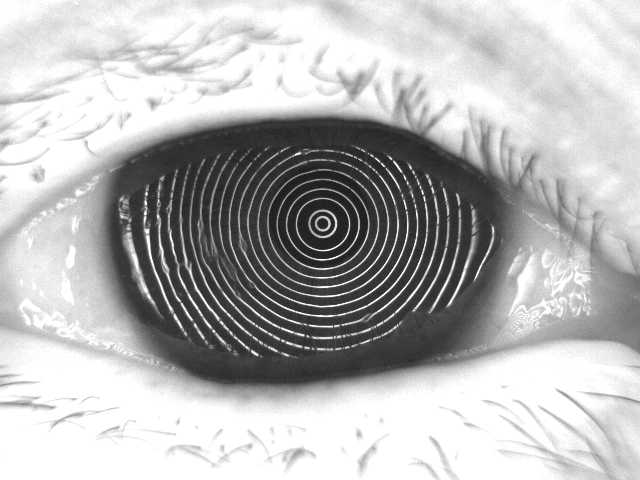

Supplement: Supplementary file 1 [file diagnostics-14-00052-s001.zip › Decreased wettability DE (DWDE)/Dimple break/0052.jpeg]

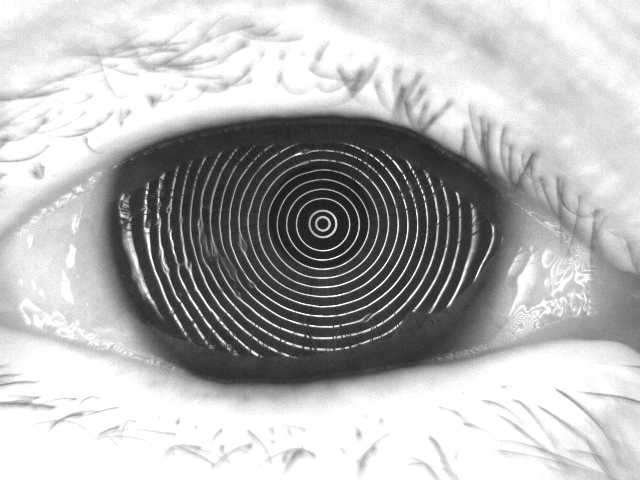

Supplement: Supplementary file 1 [file diagnostics-14-00052-s001.zip › Decreased wettability DE (DWDE)/Dimple break/0053.jpeg]

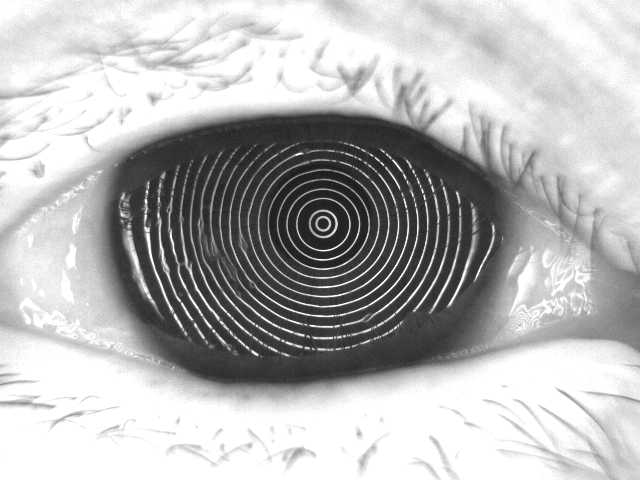

Supplement: Supplementary file 1 [file diagnostics-14-00052-s001.zip › Decreased wettability DE (DWDE)/Dimple break/0054.jpeg]

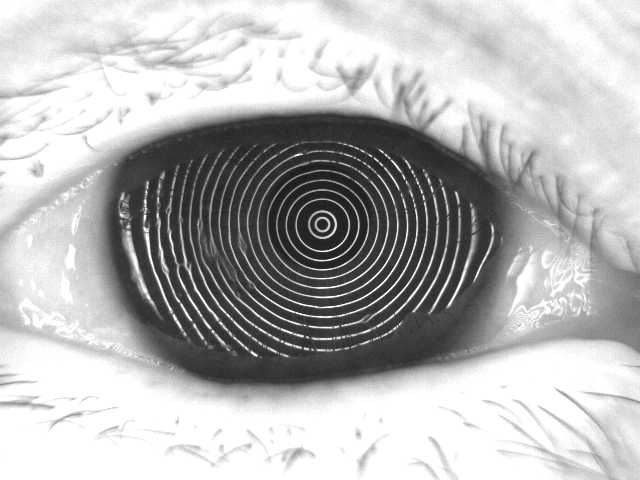

Supplement: Supplementary file 1 [file diagnostics-14-00052-s001.zip › Decreased wettability DE (DWDE)/Dimple break/0055.jpeg]

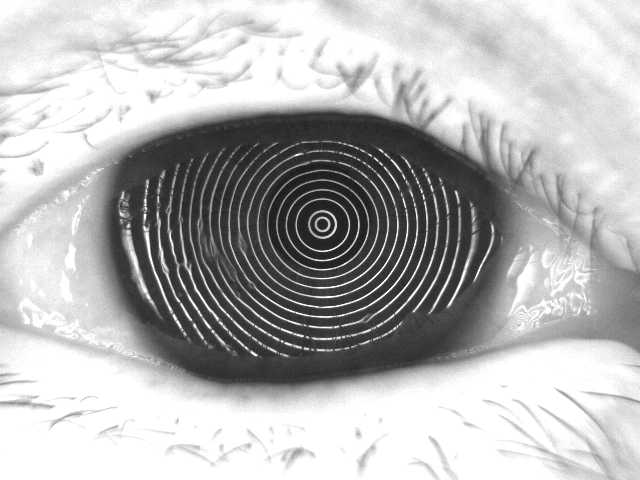

Supplement: Supplementary file 1 [file diagnostics-14-00052-s001.zip › Decreased wettability DE (DWDE)/Dimple break/0056.jpeg]

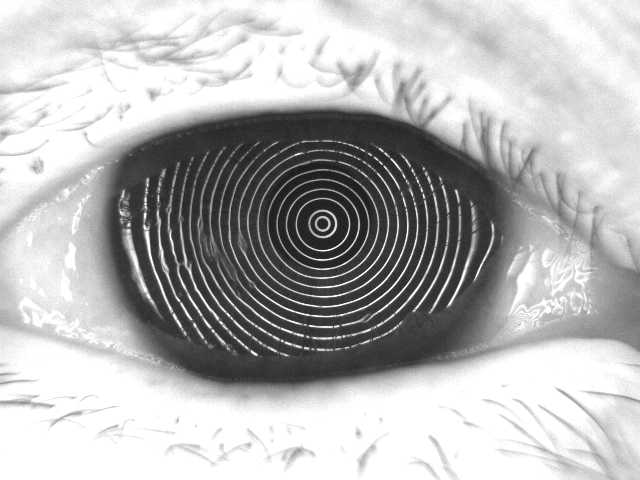

Supplement: Supplementary file 1 [file diagnostics-14-00052-s001.zip › Decreased wettability DE (DWDE)/Dimple break/0057.jpeg]

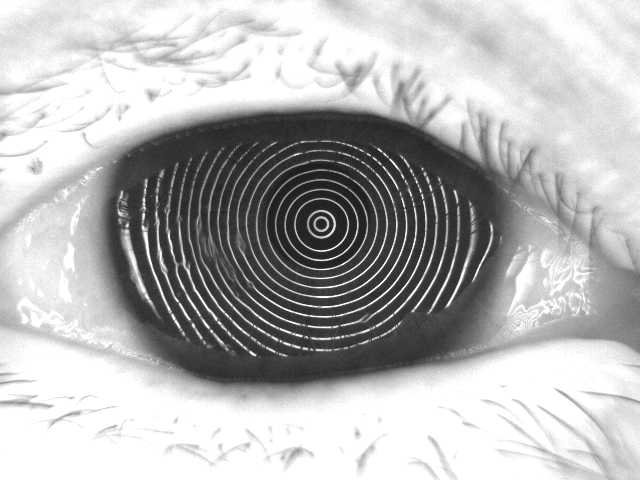

Supplement: Supplementary file 1 [file diagnostics-14-00052-s001.zip › Decreased wettability DE (DWDE)/Dimple break/0058.jpeg]

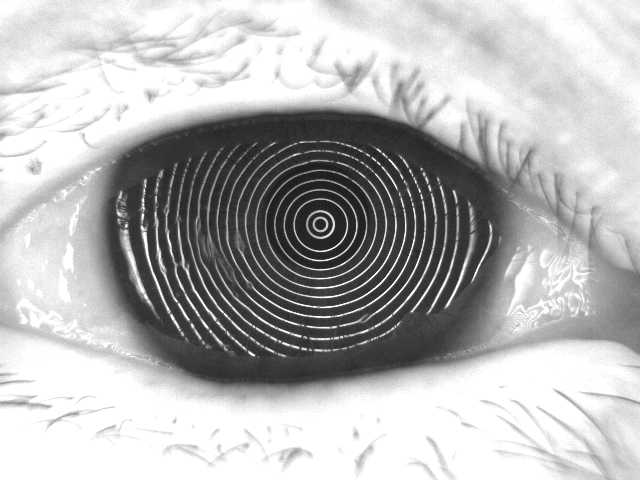

Supplement: Supplementary file 1 [file diagnostics-14-00052-s001.zip › Decreased wettability DE (DWDE)/Dimple break/0059.jpeg]

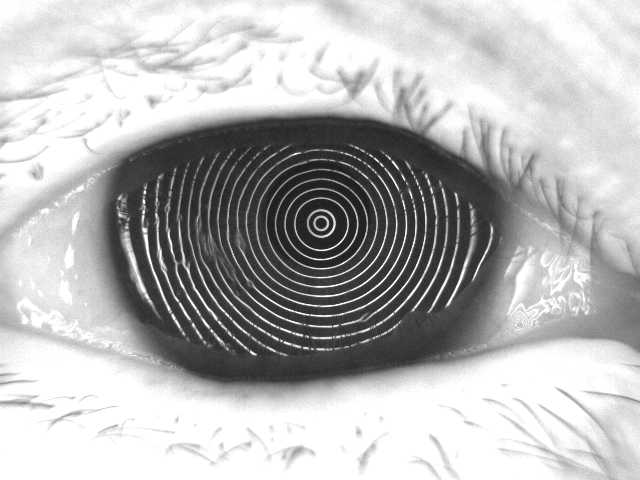

Supplement: Supplementary file 1 [file diagnostics-14-00052-s001.zip › Decreased wettability DE (DWDE)/Dimple break/0060.jpeg]

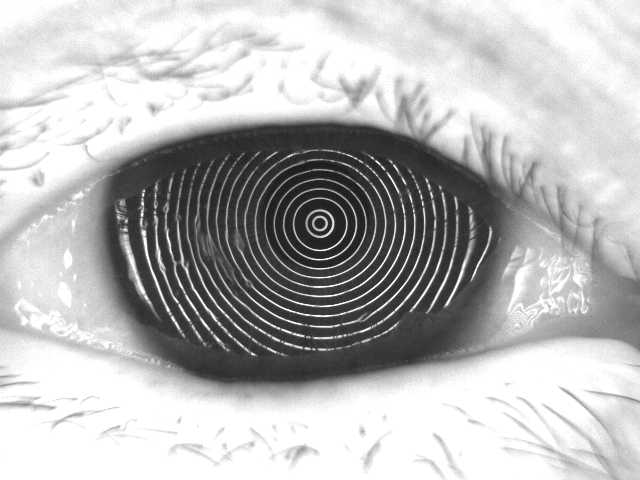

Supplement: Supplementary file 1 [file diagnostics-14-00052-s001.zip › Decreased wettability DE (DWDE)/Dimple break/0061.jpeg]

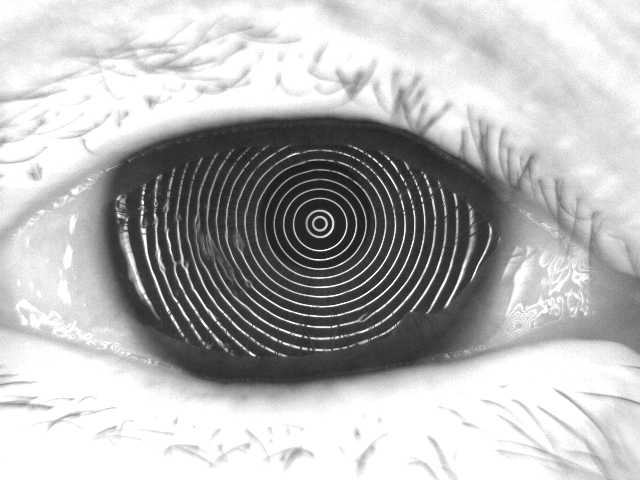

Supplement: Supplementary file 1 [file diagnostics-14-00052-s001.zip › Decreased wettability DE (DWDE)/Dimple break/0062.jpeg]

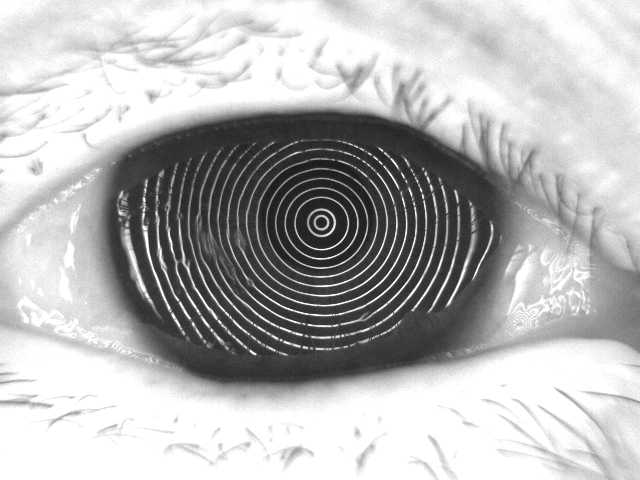

Supplement: Supplementary file 1 [file diagnostics-14-00052-s001.zip › Decreased wettability DE (DWDE)/Dimple break/0063.jpeg]

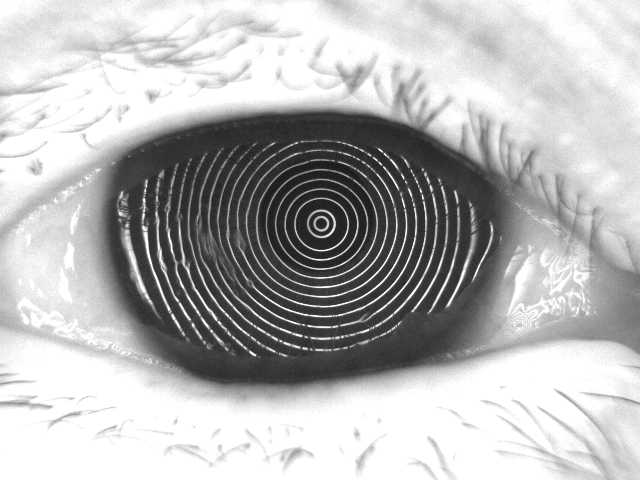

Supplement: Supplementary file 1 [file diagnostics-14-00052-s001.zip › Decreased wettability DE (DWDE)/Dimple break/0064.jpeg]

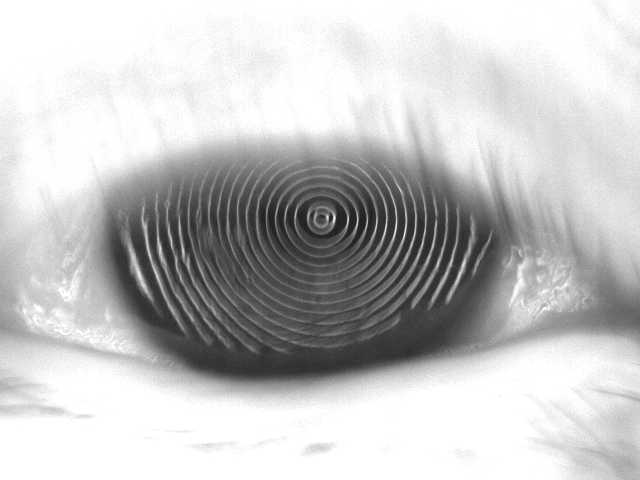

Supplement: Supplementary file 1 [file diagnostics-14-00052-s001.zip › Decreased wettability DE (DWDE)/Dimple break/0065.jpeg]

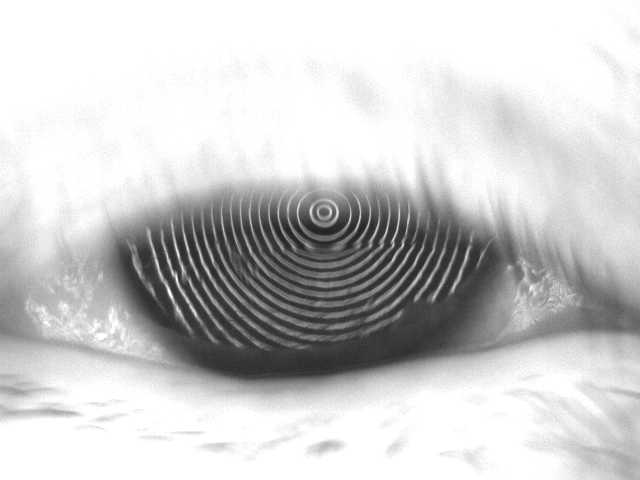

Supplement: Supplementary file 1 [file diagnostics-14-00052-s001.zip › Decreased wettability DE (DWDE)/Dimple break/0066.jpeg]

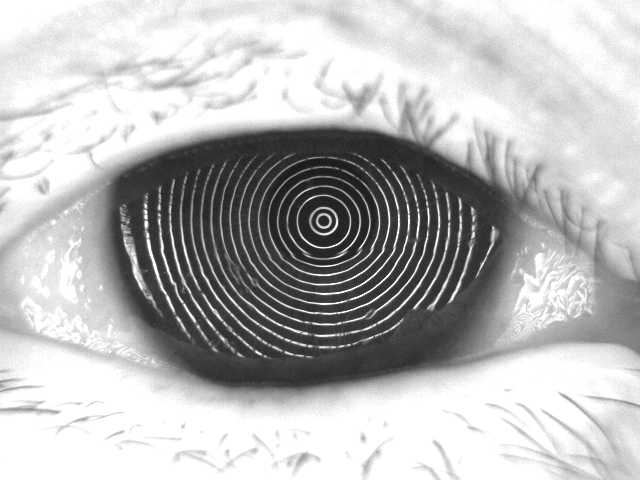

Supplement: Supplementary file 1 [file diagnostics-14-00052-s001.zip › Decreased wettability DE (DWDE)/Dimple break/0067.jpeg]

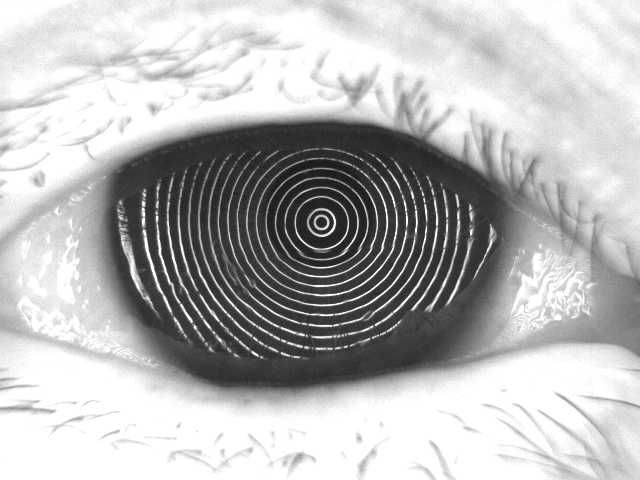

Supplement: Supplementary file 1 [file diagnostics-14-00052-s001.zip › Decreased wettability DE (DWDE)/Dimple break/0068.jpeg]

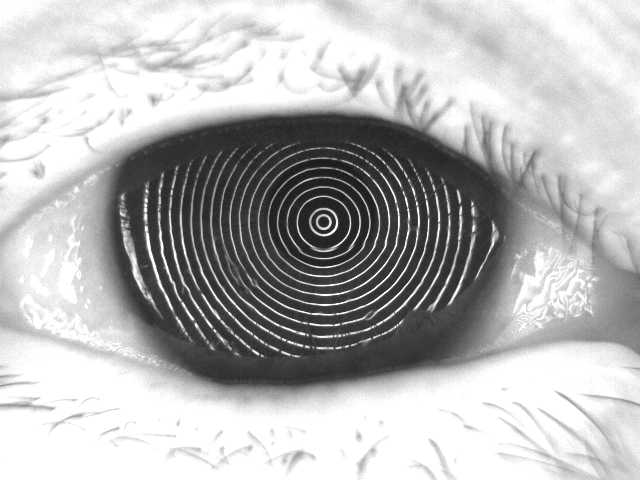

Supplement: Supplementary file 1 [file diagnostics-14-00052-s001.zip › Decreased wettability DE (DWDE)/Dimple break/0069.jpeg]

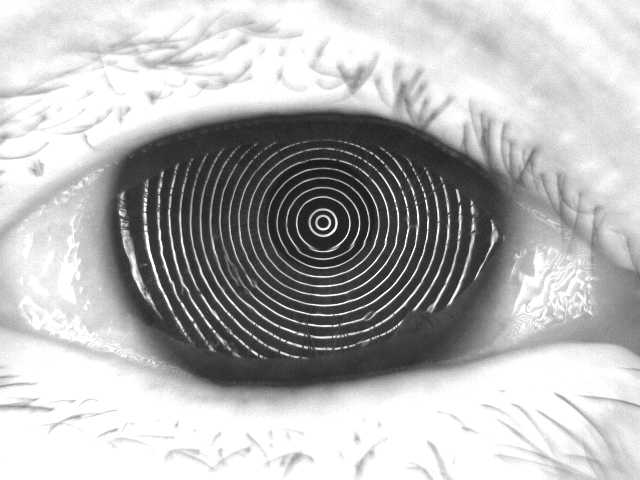

Supplement: Supplementary file 1 [file diagnostics-14-00052-s001.zip › Decreased wettability DE (DWDE)/Dimple break/0070.jpeg]

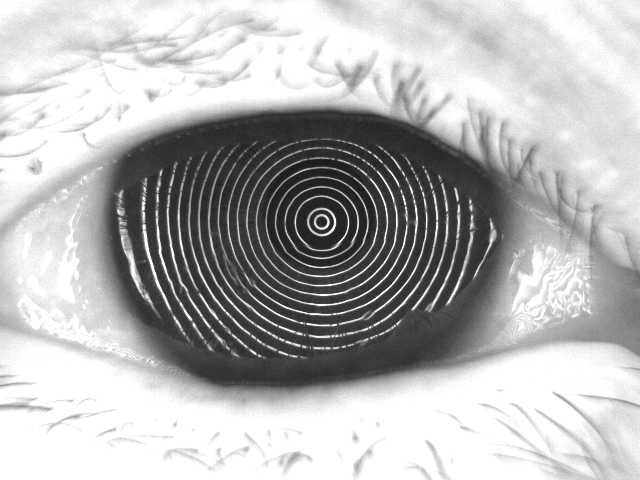

Supplement: Supplementary file 1 [file diagnostics-14-00052-s001.zip › Decreased wettability DE (DWDE)/Dimple break/0071.jpeg]

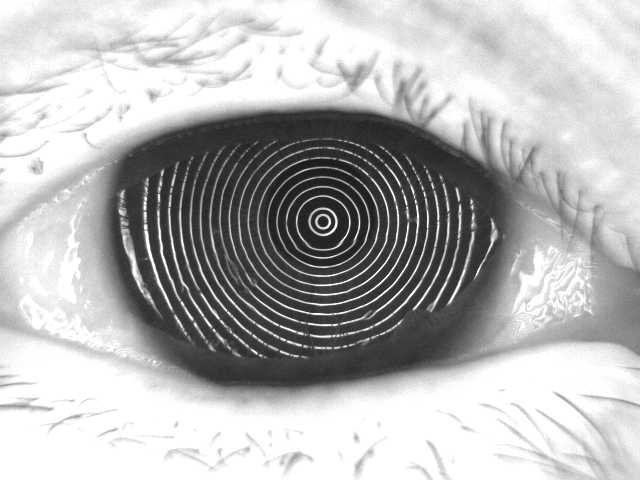

Supplement: Supplementary file 1 [file diagnostics-14-00052-s001.zip › Decreased wettability DE (DWDE)/Dimple break/0072.jpeg]

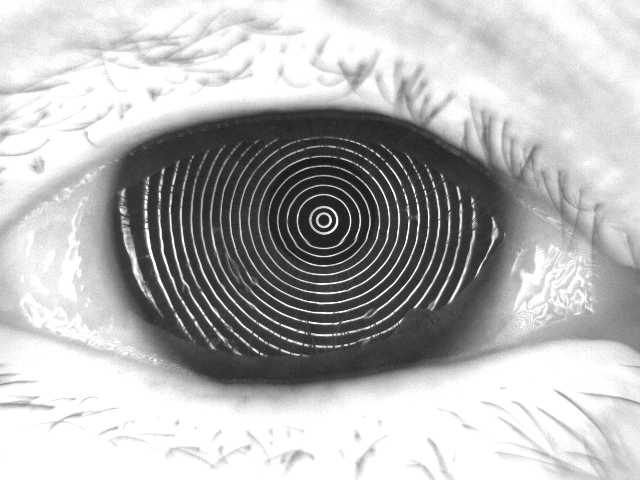

Supplement: Supplementary file 1 [file diagnostics-14-00052-s001.zip › Decreased wettability DE (DWDE)/Dimple break/0073.jpeg]

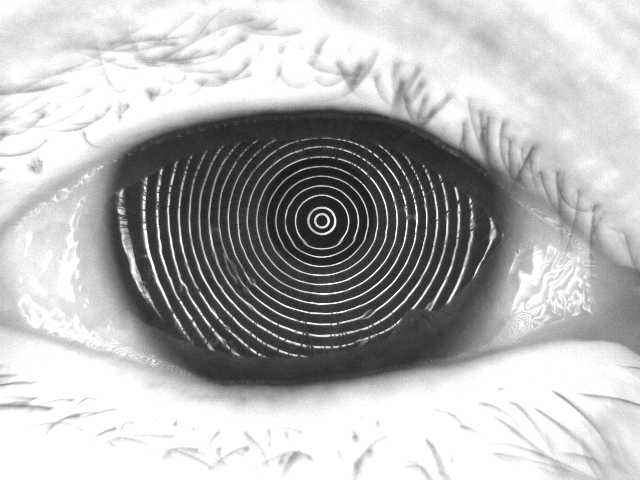

Supplement: Supplementary file 1 [file diagnostics-14-00052-s001.zip › Decreased wettability DE (DWDE)/Dimple break/0074.jpeg]

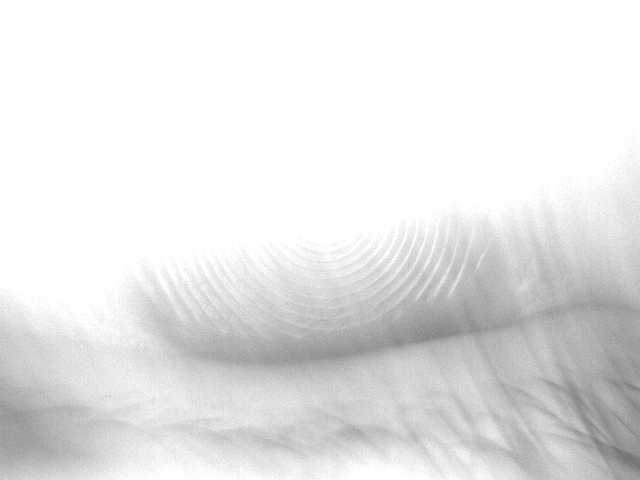

Supplement: Supplementary file 1 [file diagnostics-14-00052-s001.zip › Decreased wettability DE (DWDE)/Dimple break/0075.jpeg]

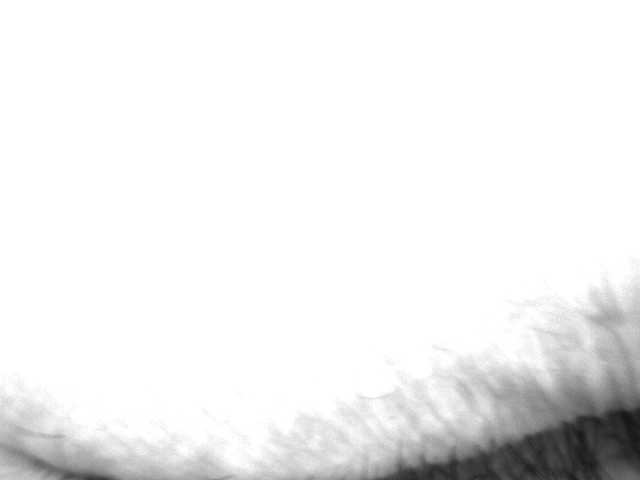

Supplement: Supplementary file 1 [file diagnostics-14-00052-s001.zip › Decreased wettability DE (DWDE)/Dimple break/0076.jpeg]

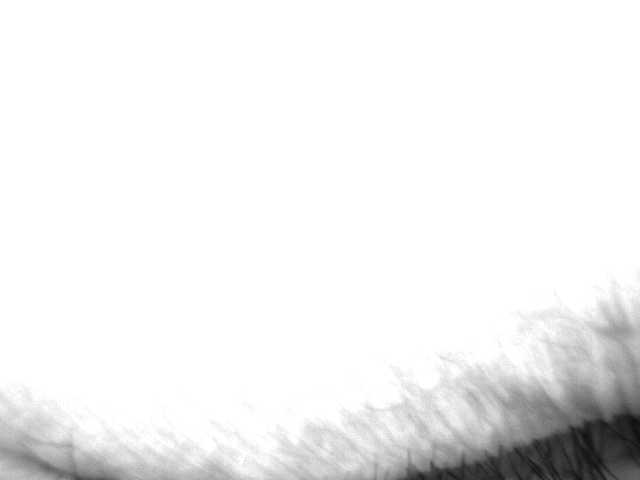

Supplement: Supplementary file 1 [file diagnostics-14-00052-s001.zip › Decreased wettability DE (DWDE)/Dimple break/0077.jpeg]

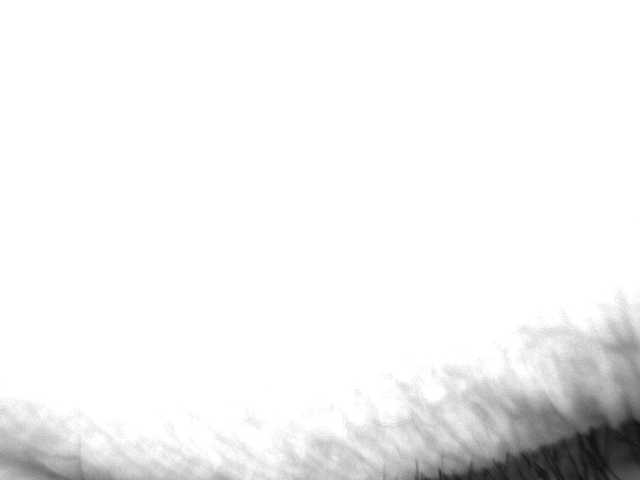

Supplement: Supplementary file 1 [file diagnostics-14-00052-s001.zip › Decreased wettability DE (DWDE)/Dimple break/0078.jpeg]

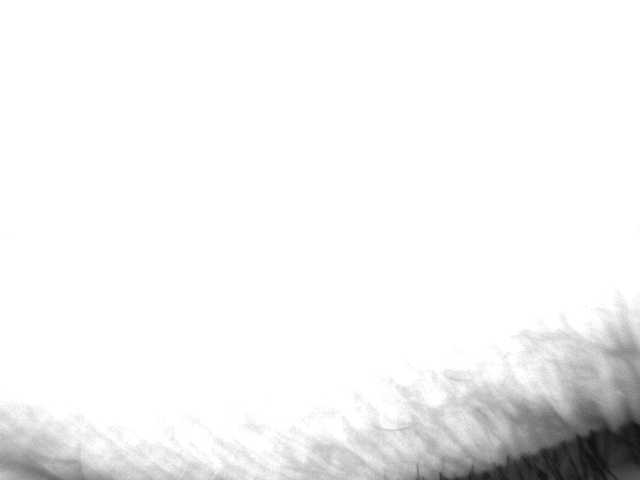

Supplement: Supplementary file 1 [file diagnostics-14-00052-s001.zip › Decreased wettability DE (DWDE)/Dimple break/0079.jpeg]

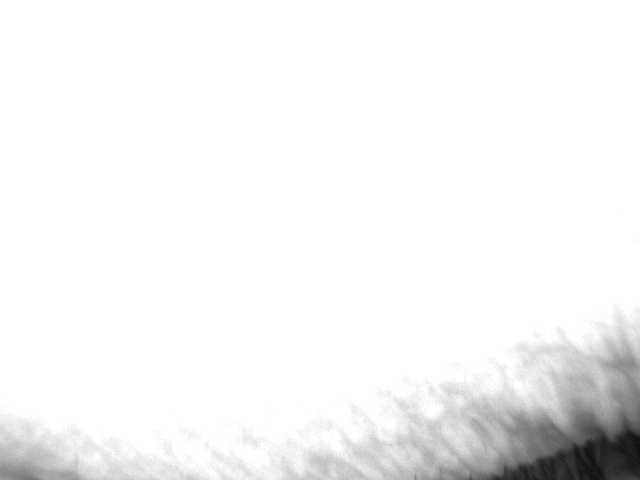

Supplement: Supplementary file 1 [file diagnostics-14-00052-s001.zip › Decreased wettability DE (DWDE)/Dimple break/0080.jpeg]

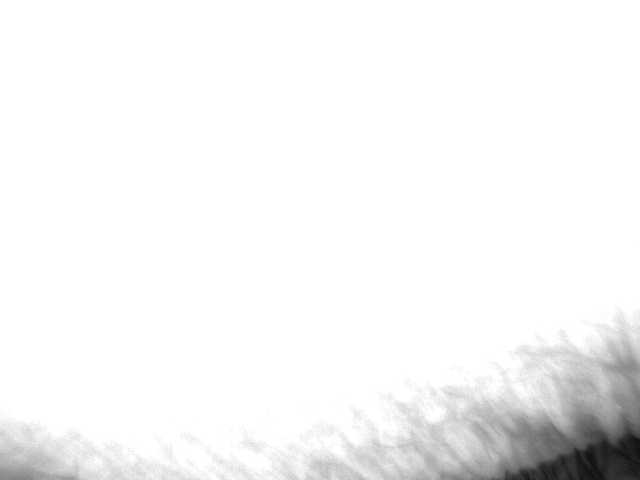

Supplement: Supplementary file 1 [file diagnostics-14-00052-s001.zip › Decreased wettability DE (DWDE)/Dimple break/0081.jpeg]

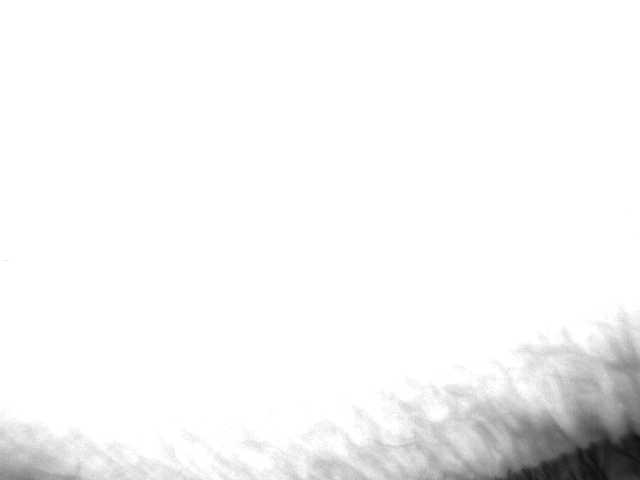

Supplement: Supplementary file 1 [file diagnostics-14-00052-s001.zip › Decreased wettability DE (DWDE)/Dimple break/0082.jpeg]

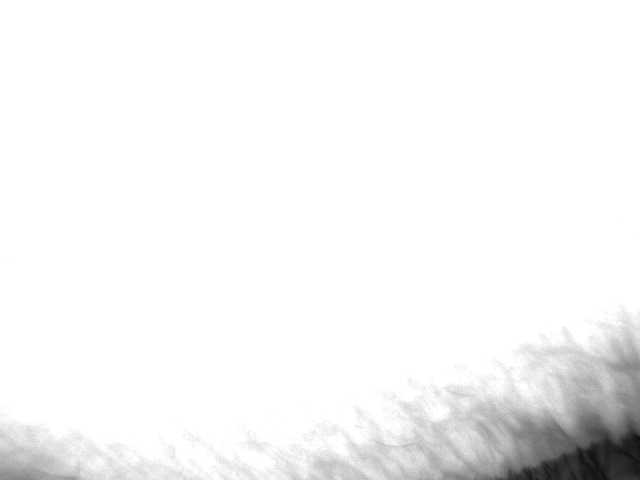

Supplement: Supplementary file 1 [file diagnostics-14-00052-s001.zip › Decreased wettability DE (DWDE)/Dimple break/0083.jpeg]

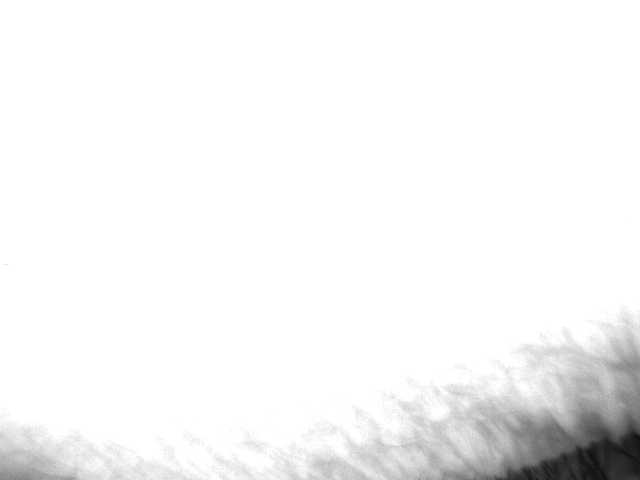

Supplement: Supplementary file 1 [file diagnostics-14-00052-s001.zip › Decreased wettability DE (DWDE)/Dimple break/0084.jpeg]

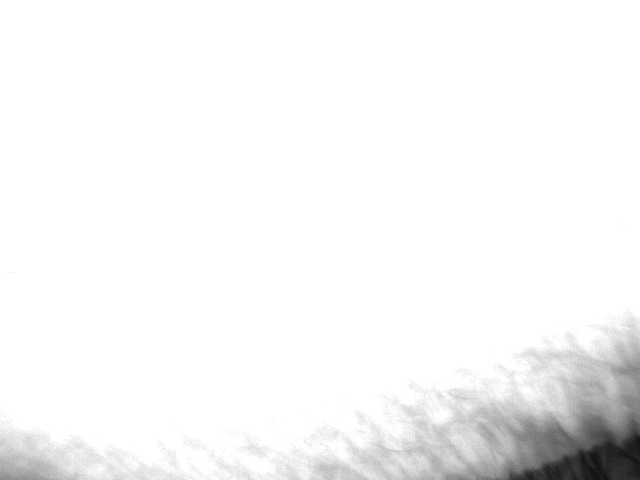

Supplement: Supplementary file 1 [file diagnostics-14-00052-s001.zip › Decreased wettability DE (DWDE)/Dimple break/0085.jpeg]

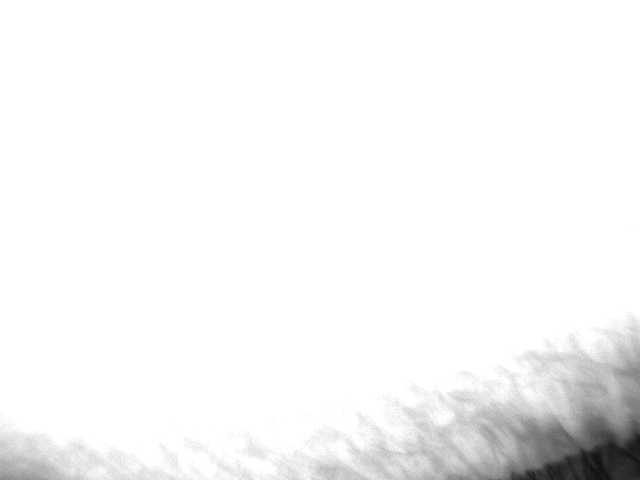

Supplement: Supplementary file 1 [file diagnostics-14-00052-s001.zip › Decreased wettability DE (DWDE)/Dimple break/0086.jpeg]

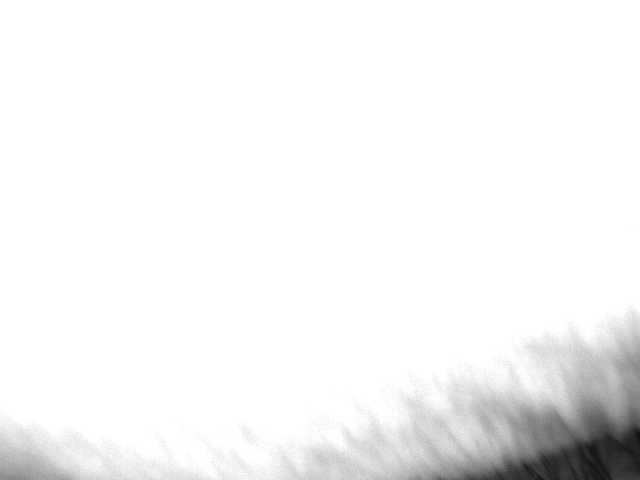

Supplement: Supplementary file 1 [file diagnostics-14-00052-s001.zip › Decreased wettability DE (DWDE)/Dimple break/0087.jpeg]

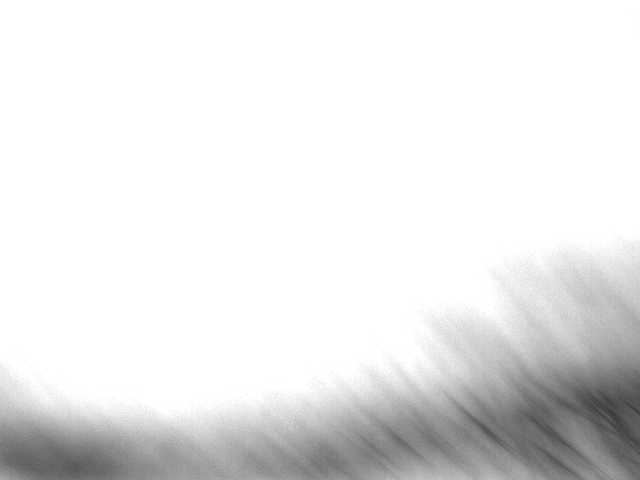

Supplement: Supplementary file 1 [file diagnostics-14-00052-s001.zip › Decreased wettability DE (DWDE)/Dimple break/0088.jpeg]

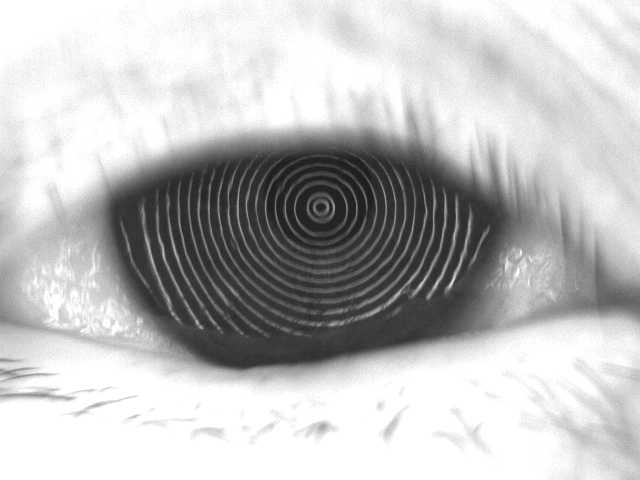

Supplement: Supplementary file 1 [file diagnostics-14-00052-s001.zip › Decreased wettability DE (DWDE)/Dimple break/0089.jpeg]

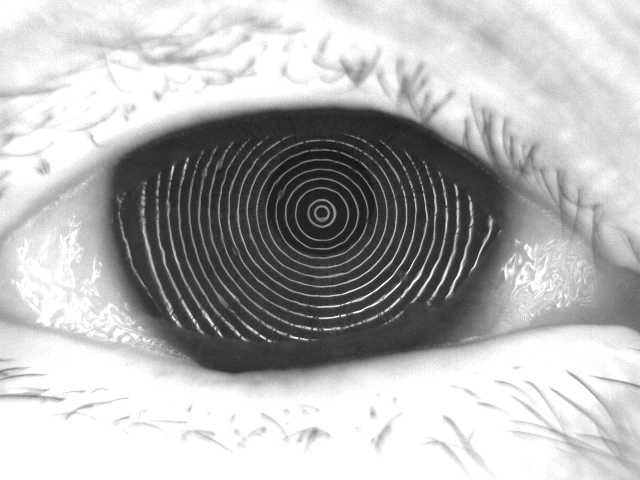

Supplement: Supplementary file 1 [file diagnostics-14-00052-s001.zip › Decreased wettability DE (DWDE)/Dimple break/0090.jpeg]

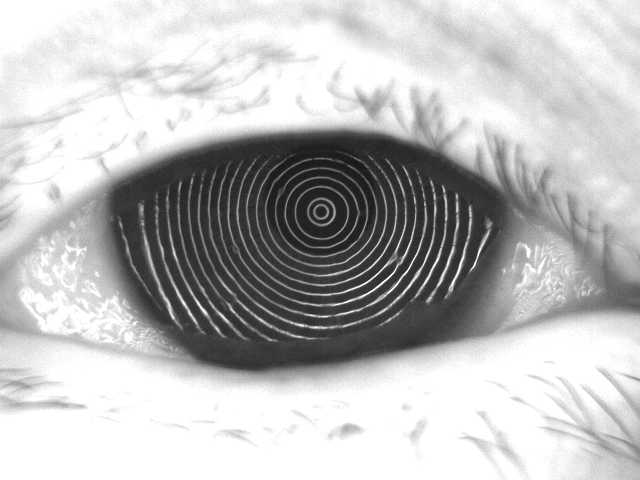

Supplement: Supplementary file 1 [file diagnostics-14-00052-s001.zip › Decreased wettability DE (DWDE)/Dimple break/0091.jpeg]

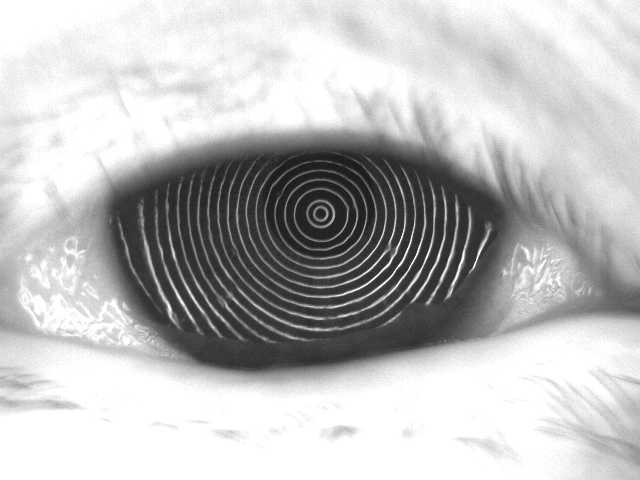

Supplement: Supplementary file 1 [file diagnostics-14-00052-s001.zip › Decreased wettability DE (DWDE)/Dimple break/0092.jpeg]

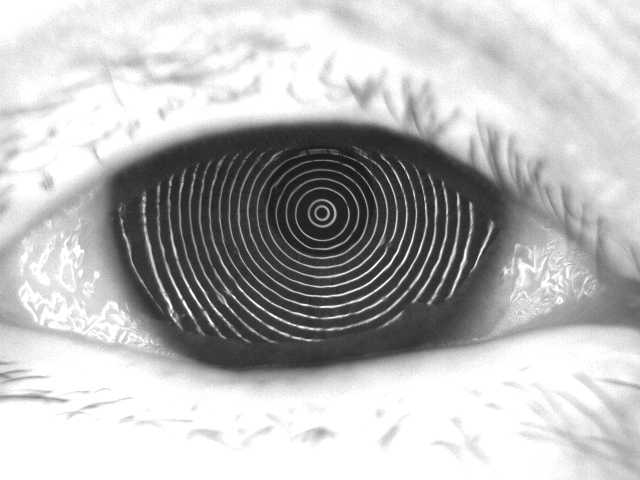

Supplement: Supplementary file 1 [file diagnostics-14-00052-s001.zip › Decreased wettability DE (DWDE)/Dimple break/0093.jpeg]

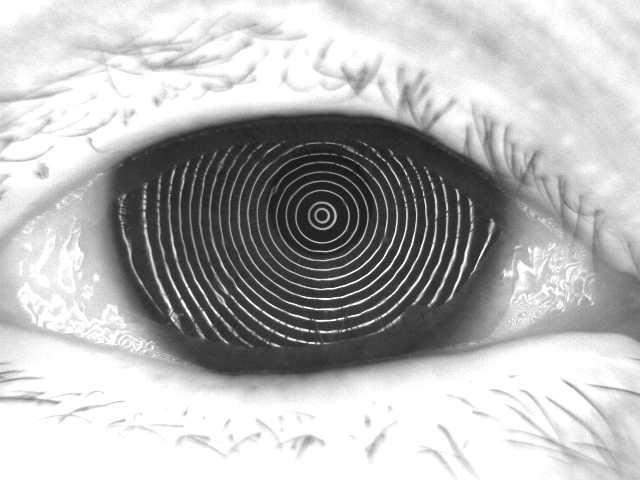

Supplement: Supplementary file 1 [file diagnostics-14-00052-s001.zip › Decreased wettability DE (DWDE)/Dimple break/0094.jpeg]

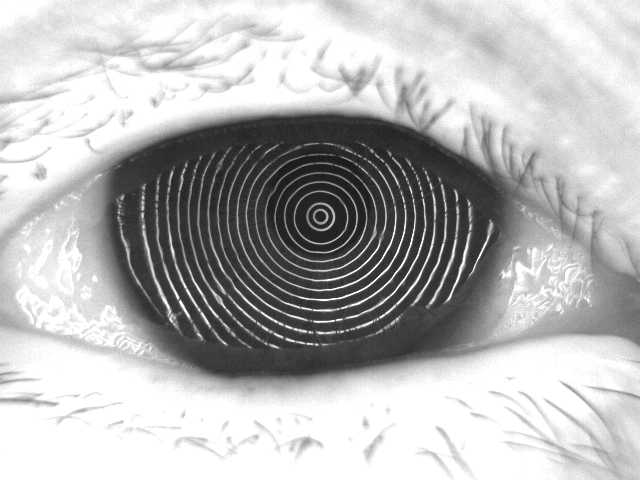

Supplement: Supplementary file 1 [file diagnostics-14-00052-s001.zip › Decreased wettability DE (DWDE)/Dimple break/0095.jpeg]

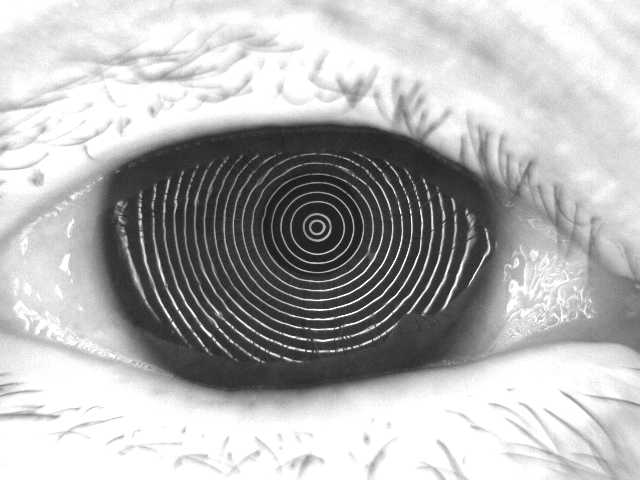

Supplement: Supplementary file 1 [file diagnostics-14-00052-s001.zip › Decreased wettability DE (DWDE)/Dimple break/0096.jpeg]

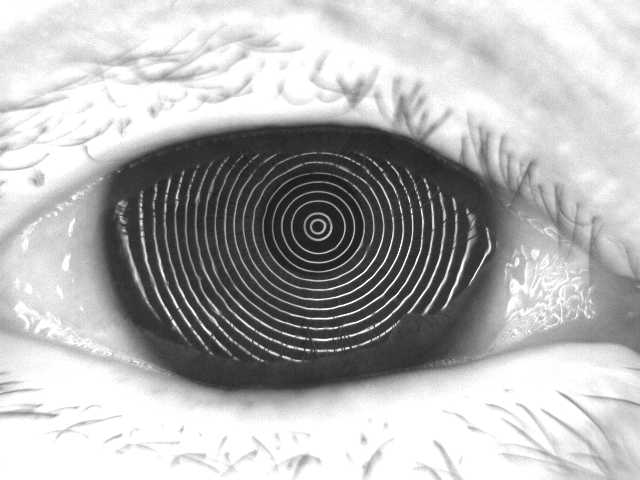

Supplement: Supplementary file 1 [file diagnostics-14-00052-s001.zip › Decreased wettability DE (DWDE)/Dimple break/0097.jpeg]

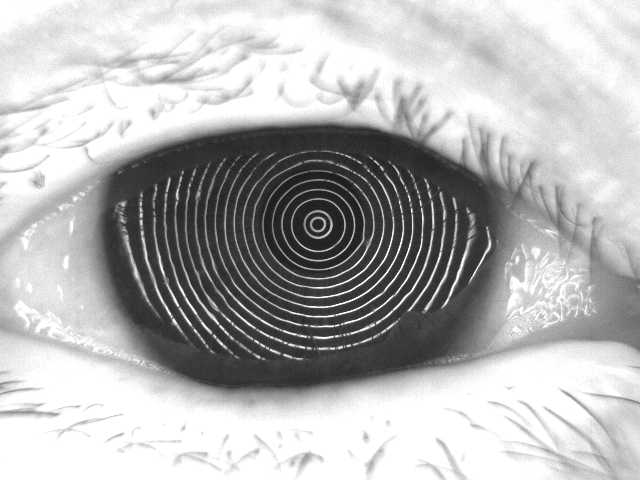

Supplement: Supplementary file 1 [file diagnostics-14-00052-s001.zip › Decreased wettability DE (DWDE)/Dimple break/0098.jpeg]

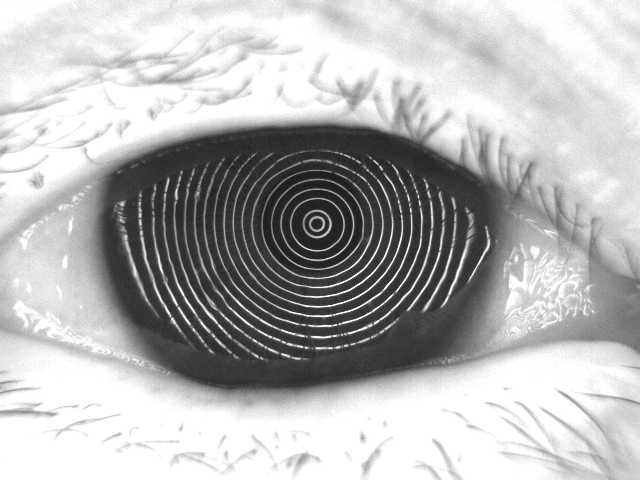

Supplement: Supplementary file 1 [file diagnostics-14-00052-s001.zip › Decreased wettability DE (DWDE)/Dimple break/0099.jpeg]
